# Specific inhibition of an anticancer target, polo-like kinase 1, by allosterically dismantling its mechanism of substrate recognition

**DOI:** 10.1073/pnas.2305037120

**Published:** 2023-08-21

**Authors:** Jung-Eun Park, Klara Kirsch, Hobin Lee, Paola Oliva, Jong Il Ahn, Harsha Ravishankar, Yan Zeng, Stephen D. Fox, Samuel A. Kirby, Pooja Badhwar, Thorkell Andresson, Kenneth A. Jacobson, Kyung S. Lee

**Affiliations:** ^a^Cancer Innovation Laboratory, Center for Cancer Research, National Cancer Institute, NIH, Bethesda, MD 20892; ^b^Laboratory of Bioorganic Chemistry, National Institute of Diabetes and Digestive and Kidney Diseases, NIH, Bethesda, MD 20892; ^c^Protein Characterization Laboratory, Frederick National Laboratory for Cancer Research, Leidos Biomedical Research, Inc., Frederick, MD 21702

**Keywords:** polo-like kinase 1, polo-box domain, mitotic kinase, allosteric inhibitor, X-ray crystallography

## Abstract

Polo-like kinase 1 (Plk1) is considered an attractive target for anticancer therapy. Over the years, studies on the noncatalytic polo-box domain (PBD) of Plk1 have raised the expectation of generating highly specific protein–protein interaction inhibitors. However, the molecular nature of the canonical PBD-dependent interaction, which requires extensive water network–mediated interactions with its phospholigands, has hampered efforts to identify small molecules suitable for Plk1 PBD drug discovery. Here, we report the identification of the first allosteric inhibitor of Plk1 PBD, called Allopole, a prodrug that can disrupt intracellular interactions between PBD and its cognate phospholigands, delocalize Plk1 from centrosomes and kinetochores, and induce mitotic block and cancer cell killing. At the structural level, its unmasked active form, Allopole-A, bound to a deep Trp-Phe-lined pocket occluded by a latch-like loop, whose adjoining region was required for securely retaining a ligand anchored to the phospho-binding cleft. Allopole-A binding completely dislodged the L2 loop, an event that appeared sufficient to trigger the dissociation of a phospholigand and inhibit PBD-dependent Plk1 function during mitosis. Given Allopole’s high specificity and antiproliferative potency, this study is expected to open an unexplored avenue for developing Plk1 PBD-specific anticancer therapeutic agents.

## Significance

The polo-box domain (PBD), which serves as the substrate-recognition domain, is an attractive target for Plk1-specific anticancer drug discovery. Over the years, more than two dozen PBD inhibitors have been reported but with limited cellular efficacy. All these inhibitors target the canonical phospholigand-binding site. Here, we report the discovery of an allosteric prodrug inhibitor against the PBD, called Allopole, whose unmasked active form, Allopole-A, binds to a previously undescribed pocket with high specificity. Allopole-A binding to the allosteric pocket dislodges a latch-like loop and consequently strips multiple water-mediated interactions required for stabilizing a phospholigand bound to the canonical site. This event disrupts PBD-dependent interactions, delocalizes Plk1, and induces mitotic arrest. This work could offer a new direction for anti-Plk1 drug discovery.

Polo-like kinase 1 (Plk1) is a mitotic serine/threonine (S/T) kinase, whose overexpression is closely associated with aggressiveness and poor prognosis for a wide spectrum of human cancers (1, 2). Not surprisingly, studies show that various cancer cells, but not their isogenic normal cells, are addicted to Plk1 overexpression for their viability (3–5). Since the reversal of an addicted state is considered an effective strategy to selectively suppress cancer cell proliferation (6–8), antagonizing Plk1 function has been considered an attractive approach to combat Plk1-addicted cancers.

Over the years, extensive efforts have been undertaken to generate Plk1 inhibitors yielding at least ten ATP-competitive inhibitors examined in clinical trials (5, 9). However, largely due to their nonspecific activity and/or more-than-acceptable dose-limiting toxicity, further advancement of these inhibitors is stalled at various stages of development. Since dose-limiting toxicity generally arises from an inhibitor’s nonspecific cross-reactivities (10), improving Plk1 specificity is likely one of the main hurdles that needs to be overcome for better therapeutic outcomes.

Plk1 contains the C-terminal noncatalytic, but functionally essential, polo-box domain (PBD) (11), which mediates interactions with its binding partners or substrates localized abundantly at centrosomes, kinetochores, and midbodies (12, 13). Subsequent studies demonstrated that PBD serves as a structurally distinct protein–protein interaction (PPI) module for binding a short phospho-S/T (i.e., p-S/T)-containing motif (14, 15). Owing to this finding, along with the anticipated promise of the superb binding specificity that PPIs can offer, PBD has drawn substantial attention as an alternative target for anti-Plk1 drug discovery.

As of today, a flurry of research has generated over two dozen inhibitors reported to target Plk1 PBD (referred to hereafter as PBD1 for simplicity) (9, 16). However, while peptide-based inhibitors largely show a high degree of anti-PBD1 activity and specificity in vitro, whether they can be readily adapted to the development of anti-Plk1 therapeutics remains unclear because of their intrinsically poor membrane permeability and bioavailability (5, 17). On the other hand, small molecule inhibitors [e.g., Poloxin (18), Poloxipan (19), MCC1019 (20), Polotyrin (21), Compound 9 (22), KBJK557 (23), and Abbapolins (24, 25)] exhibit only a limited potency and/or specificity against PBD1, often requiring a high dose (IC_50_ of 25 to approximately 500 μM) to inhibit proliferation at the cultured cell level. Furthermore, the alkylating activity of Poloxin-type inhibitors onto nucleophilic residues (26, 27) and a somewhat less discrete binding mode of two cocrystallized compounds, Thymoquinone and Polotyrin (PDB 4HCO and 5NEI, respectively), make further development difficult.

Here, we report the generation of Allopole, a prodrug PBD1-specific inhibitor whose active species (i.e., Allopole-A; referring to the active form of Allopole) bound to a previously unidentified pocket. Interestingly, this allosteric pocket is occluded by the L2 loop, which extends across the canonical phosphoepitope-binding cleft (14). Analysis of the cocrystal structure of PBD1 in complex with Allopole-A showed that Allopole binding to the pocket dislodges the L2 loop of the PBD1, consequently stripping multiple water-mediated interactions between the L2 and a phospholigand to cause the ligand to dissociate from the PBD1. In line with these observations, Allopole disrupted PBD1-dependent subcellular localization of Plk1 within 2 h, imposed mitotic arrest, and inhibited cell proliferation in cultured cells. With the unique binding mode of Allopole-A and the deep pocket to which it binds, this work may offer an unexplored direction that could lead to the development of a new class of anti-Plk1 therapeutics.

## Results

### Discovery of Allopole, Exhibiting an Improved Anti-PBD1 Activity In Vitro and in Cells.

**Fig. 1.**
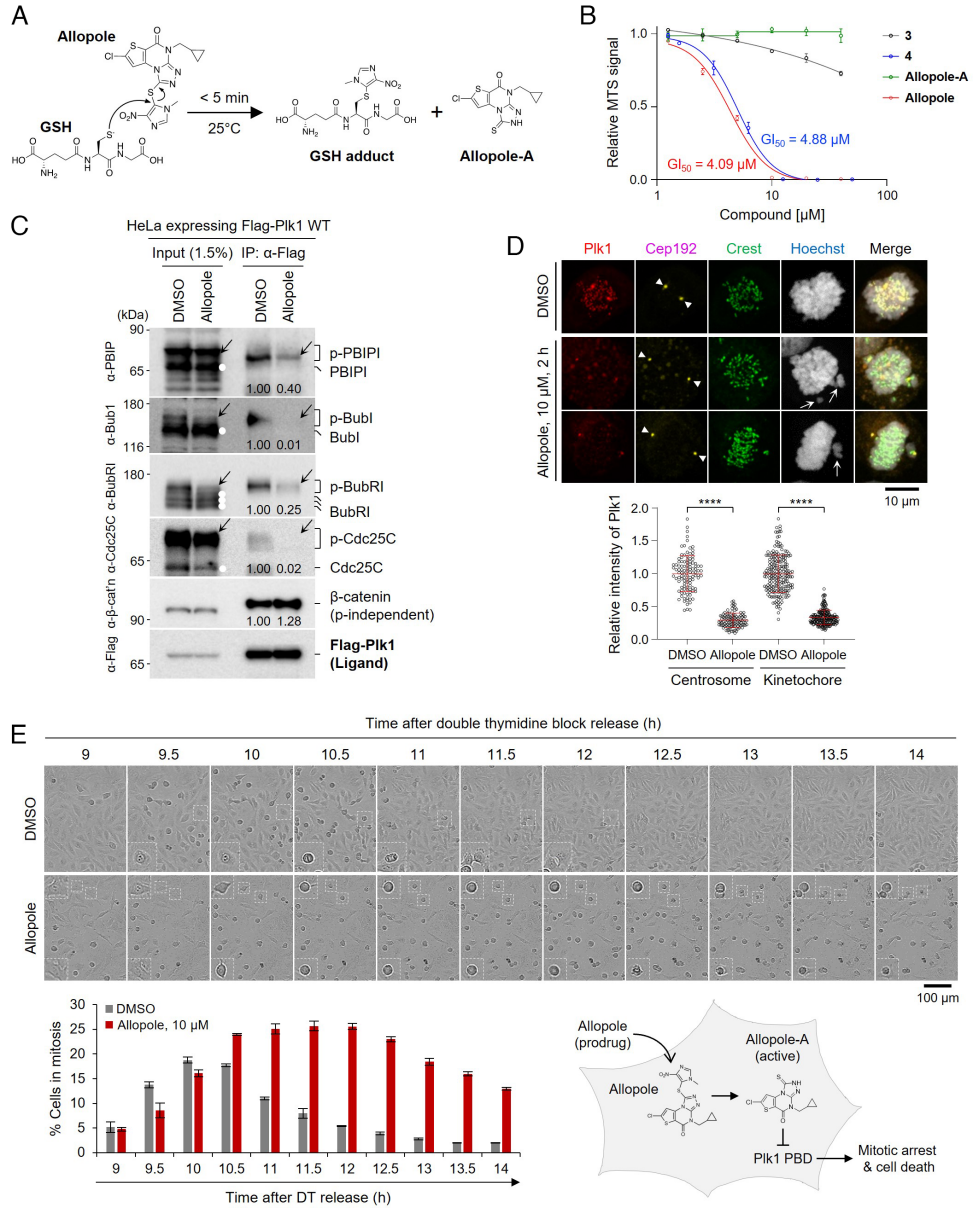
Allopole inhibits PBD1-dependent interactions and subcellular localizations, consequently inducing mitotic block in HeLa cells. (*A*) Schematic diagram depicting how the prodrug moiety of Allopole is released by GSH. (*B*) MTS assays carried out to determine the potency of two selected prodrugs (Allopole and **4**) and their respective active species (Allopole-A and **3**) on the proliferation of multiple myeloma-derived L363 cells. The resulting data were quantified from three independent experiments performed two days after treating the cells with different concentrations of the compounds. Bars, mean of three experiments ± SD. Quantified data are provided in *SI Appendix,* Table S1, along with other related compounds. (*C*) Coimmunoprecipitation (IP) and immunoblotting analyses were performed using HeLa cells expressing Flag-Plk1 and treated with control DMSO or 20 μM of Allopole for 2 h before harvest. To enrich the level of phosphorylated proteins, the cells were additionally treated with 600 nM of nocodazole and 300 nM of okadaic acid (OA) for 16 h and 1.5 h, respectively, before harvest. Both phosphorylated (arrows) and un/underphosphorylated (white dots) species for each protein are marked. β-catenin, which binds to PBD1 in a phosphorylation-independent manner (33), serves as a control. Numbers, relative signal intensities. (*D*) Confocal imaging and quantification of Plk1 signal intensities at centrosomes and kinetochores after treating HeLa cells with DMSO or 10 μM of Allopole for 2 h. Arrowheads, centrosomes; arrows, misaligned chromosomes. Signal intensities were quantified from both centrosome signals and randomly chosen three to four kinetochore signals per cell for a total of greater than or equal to 18 cells/experiment (n = three independent experiments). ****P < 0.0001 (unpaired two-tailed *t* test). Bars, mean ± SD. (*E*) Time-lapse analyses for HeLa cells released from a double-thymidine (DT) block (G1/S) and treated with DMSO or Allopole 7 h after release. Dotted boxes, enlarged cells exhibiting normal mitotic progression (DMSO) or cell death following mitotic block (Allopole). Cells in mitosis were quantified from three independent experiments (for control DMSO, 938 ≤ n ≤ 1,685 cells/time point/experiment; for Allopole, 806 ≤ n ≤ 1,397 cells/time point/experiment). Bars, mean ± SD. The schematic diagram (right, bottom) depicts conversion of Allopole to the active Allopole-A species after entering into the cell (gray).

We previously reported the identification of triazoloquinazolinone-based small molecular ligands that exhibit anti-PBD1 activity in vitro (28). Subsequent efforts to improve the potency of these inhibitors yielded a monoheterocyclic aromatic ring-containing compound **3** and its prodrug **4** conjugated with the 5-thio-1-methyl-4-nitroimidazolyl moiety (28, 29) (*SI Appendix,* Table S1). However, the potential application of these compounds has been severely hampered because of their undefined mode of action and lower-than-acceptable solubility (*SI Appendix,* Fig. S1 *A* and *B*). Therefore, through an extensive optimization campaign, we generated Allopole (Fig. 1*A*) with greatly increased solubility (*SI Appendix,* Fig. S1 *A* and *B*). Allopole is expected to be rapidly converted to its unmasked nonprodrug form, Allopole-A, under reducing conditions. Indeed, an hour exposure of Allopole to 5 mM glutathione (GSH), the concentration found in most intracellular environments (30), was sufficient to completely unmask Allopole under several physiologically relevant buffer conditions at room temperature (*SI Appendix,* Table S2). Additional analyses showed that GSH efficiently generated an adduct with the 5-thio-1-methyl-4-nitroimidazolyl prodrug-masking moiety (Fig. 1*A* and *SI Appendix,* Fig. S1*C*) and converted a prodrug form into an active species within 5 min at room temperature in vitro (*SI Appendix,* Fig. S1*D*). Taken together, these observations suggest that a nucleophilic attack at the C5 position of the masking group by GSH is likely the primary mechanism of releasing the active Allopole-A species, as demonstrated previously for an analogous prodrug, azathioprine (31, 32).

Additional characterization with Allopole showed that when compared to **4**, it exhibited an improved anti-cell-proliferation activity with a 50% cell growth inhibition (GI_50_) value of 4.1 μM in multiple myeloma-derived L363 cells (34) (Fig. 1*B*). Their respective nonprodrugs (i.e., **3** and Allopole-A, respectively) failed to induce any significant antiproliferation effect (Fig. 1*B*), suggesting that they are not cell membrane-permeable unless they are masked with the prodrug moiety. In an in vitro ELISA (15), Allopole-A, the nonprodrug form, showed an improved anti-PBD1 activity (with a half maximal inhibitory concentration [IC_50_] value of 1.4 μM), outperforming **3** (IC_50_ value of 2.15 μM) by approximately 50% (*SI Appendix,* Fig. S1*E* and Table S1). Quantified data for all Allopole-related compounds are listed in *SI Appendix,* Table S1. Collectively, while all prodrugs failed to show any significant level of anti-PBD1 activity in in vitro ELISAs, they imposed a potent antiproliferation effect in cell-based assays. These observations suggest that prodrug masking for **3** and Allopole-A is critical for overcoming their low cell membrane permeability.

In a set of fluorescence polarization (FP)-based assays designed to determine the binding affinity to each of three structurally related PBDs from Plk1−3 (35, 36) (in short, PBD1−PBD3), Allopole-A exhibited a superb specificity against PBD1 (*SI Appendix,* Fig. S1*F*) with a calculated IC_50_ value of 2.5 nM, a significant improvement from 10.2 nM observed with **3** (*SI Appendix,* Table S3). When compared to a PBIP1 p-T78 motif-derived ligand, PLHSpT (IC_50_ of 35.0 nM), which binds to PBD1 with a K_d_ of 450 nM (15), Allople-A had higher affinity by at least an order of magnitude (*SI Appendix,* Table S3). In addition, the specificity of Allopole-A was confirmed by the observation that biotin-conjugated **22** precipitated the full-length Plk1, but not Plk2 and Plk3, from the total lysates expressing all three kinases at similar levels (*SI Appendix,* Fig. S1*G*). Given that the two closely related polo-like kinases, Plk2 and Plk3, are proposed to play tumor suppressor roles (37, 38), the Plk1-specific inhibitory activity is an important property of Allopole-A. The chemical synthesis of these inhibitors is described in detail in *SI Appendix*.

### Allopole Disrupts Various PBD1-Dependent Interactions and Inhibits Plk1 Localization and Function.

To investigate the capacity of Allopole to inhibit PBD-dependent Plk1 function, we examined whether Allopole can disrupt the interactions between PBD1 and its cognate binding targets. To increase the level of phosphorylated epitopes required for p-S/T-dependent PBD1 binding (14), cells were treated with nocodazole, a microtubule destabilizer (39), and okadaic acid, an inhibitor of protein phosphatases PP2A and PP1 (40). Under these conditions, Flag-tagged Plk1 effectively coimmunoprecipitated phosphorylated (p-) PBIP1/CENP-U (41), Bub1 (42), BubR1 (43), and Cdc25C (44) (phosphorylated forms are marked with arrows) but not their respective unphosphorylated and underphosphorylated forms (Fig. 1*C*). Treatment of cells with 20 μM of Allopole for 2 h greatly diminished the amounts of these proteins coprecipitating with Plk1. As expected, the level of coprecipitating β-catenin, which interacts with PBD1 in a phosphorylation-independent manner (33), remained unchanged (Fig. 1*C*, fifth panel).

We then examined whether Allopole can induce delocalization of Plk1 from centrosomes and kinetochores, the two prominent locations to which Plk1 is abundantly recruited in a PBD-dependent manner during early mitosis (13, 45, 46). Following 2-h treatment with 10 μM of Allopole, Plk1 signals localized at centrosomes and kinetochores were greatly diminished (Fig. 1*D*). This finding suggests that considering the time required for Allopole to cross the membrane and release its prodrug moiety, Allopole converted rapidly into the active Allopole-A form (presumably by intracellular GSH) and antagonized the PBD1 function. In line with this finding, time-lapse analysis performed with double thymidine-released cells showed that Allopole effectively blocked mitotic progression for several hours and induced cell death (Fig. 1*E*), the effect that would be expected if Allopole inhibits the interactions between PBD1 and its cognate substrates (as shown in Fig. 1*C*). Thus, Allopole can effectively enter cells and interfere with PBD1 function (Fig. 1*E*, schematic diagram).

### Allopole-A Dislodges the L2 Loop of PBD1 Critical for Water-Mediated Interactions with a Phospholigand.

**Fig. 2.**
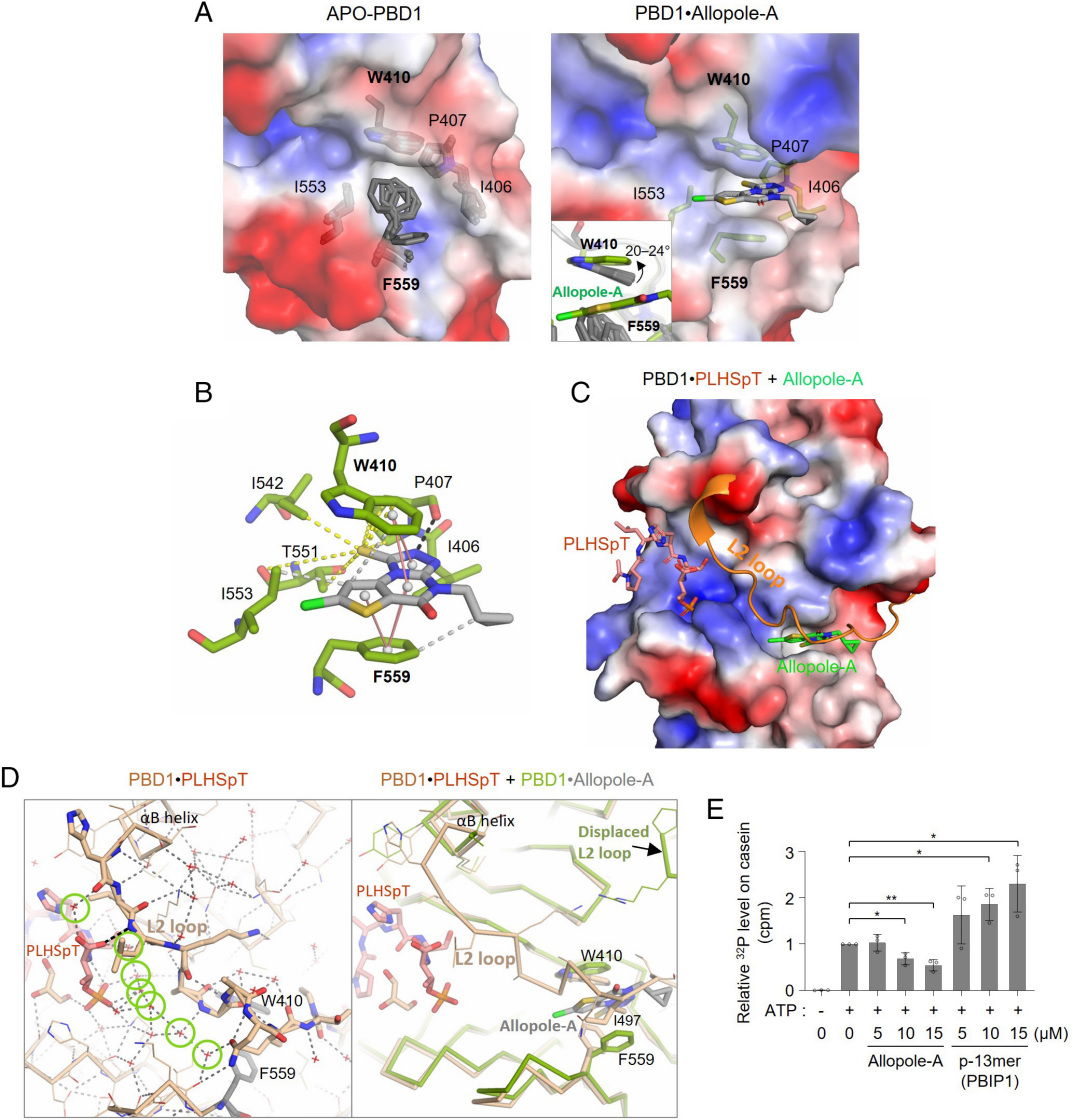
Allopole dislodges the L2 loop from the canonical phosphoepitope-binding cleft of PBD1. (*A*) Electrostatic surface representation of an ensemble of apo-PBDs (*Left*, PDBs 1Q4O, 2OGQ, 3P2W, 4H5X, 5NN1, and 3HIH) and the PBD1•Allopole-A complex (*Right*; PDB 8CRC). The flexible F559 becomes rigid upon Allopole-A binding. The W410 tilted approximately 20 to 24° to establish the π–π interaction with Allopole-A (*Inset* in the *Right* panel). (*B*) Schematic representation of the interactions between Allopole-A and the residues in the allosteric W-F pocket. π–π interactions (solid salmon lines), an H-bond (the black dashed line with P407), contacts within 4 Å around the thioxo group (yellow dashed lines), and hydrophobic interactions (<4 Å, gray dashes) are indicated. (*C*) Overlay of the PBD1•PLHSpT complex (PDB 3HIK) with Allopole-A bound to the W-F pocket. The Allopole-A binding pocket is directly under the L2 loop that secures the PLHSpT binding through its upstream adjoining region. (*D*) Model showing that the L2 loop, which traverses the periphery of the canonical phosphoepitope-binding cleft, interacts with a phospholigand (PLHSpT) through a network of multiple water molecules (green circles) and an H-bond (black dashed line) (*Left*). Binding of Allopole-A to the W-F pocket displaces the L2 loop (green) and disrupts these interactions with PLHSpT (*Right*). (*E*) Quantification of Plk1 kinase activity determined by in vitro kinase assays performed in the presence of the indicated concentration of Allopole-A or the p-13mer peptide derived from the p-T78 motif of PBIP1 (49). Representative autoradiography and Coomassie Brilliant Blue–stained gel are provided in *SI Appendix,* Fig. S2*F*. ^32^P signals from the phosphorylated casein were quantified from three independent experiments. *P < 0.05, **P < 0.01 (unpaired two-tailed *t* test). Bars, mean of three experiments ± SD.

To characterize the binding mode of Allopole-A to PBD1, we determined the cocrystal structure of the PBD1•Allopole-A complex at 1.65 Å resolution (*SI Appendix,* Table S4). After molecular replacement using the PBD domain from 3HIK, we found that the L2 loop from 3HIK showed negative difference electron density on the *Fo–Fc* map, except at an occluded pocket where a clear, planar-shaped, positive difference density appeared. After deletion of the L2 loop, this density was successfully modeled with Allopole-A bound (Fig. 2*A* and *SI Appendix,* Fig. S2*A*). Analysis of the obtained structure showed that the two aromatic residues, W410 and F559, located 12.5 Å (Cα-Cα distance) away from each other, were critically required for Allopole-A binding (Fig. 2*A*). Interestingly, upon Allopole-A binding, the F559 phenyl ring present in multiple conformational isomers (Fig. 2 *A*, *Left*) oriented itself parallel to the W410 indole ring to establish a π–π interaction with the heterocyclic aromatic ring of Allopole-A (Fig. 2 *A*, *Right*). The indole ring of the W410 also tilted more than 20° to form another layer of the π–π interaction with the heterocyclic aromatic ring of Allopole-A (Fig. 2 *A*, *Inset*). This induced-fit type of interaction, in which the binding event is accompanied by target protein’s conformational changes, is considered a widespread mechanism of molecular recognition in biology (47). The W410 and F559 residues were surrounded by multiple hydrophobic residues (namely, I406, P407, I542, and I553), thus forming a water-shunning deep pocket (referred to hereafter as the W-F pocket). Moreover, P407 carbonyl established an H-bond with the Allopole-A triazole ring (Fig. 2*B* and *SI Appendix,* Fig. S2*B*). Analyses of conformational ensembles generated by the RDKit rdk_confgen.py script (http://www.rdkit.org) (48) showed that because of the van der Waals (VDW) clashes incurred by the prodrug moiety, Allopole cannot fit into the W-F pocket (*SI* *Appendix,* Fig. S2*C*).

Although the presence of bound Allopole-A was clear with a distinct electron density (*Fo–Fc* omit map; an average B factor of 27.6), the chlorine atom of Allopole-A was less apparent with the B factor of 86.0. Recrystallizing with Allopole-A containing an undetectable level of the dechlorinated form (*SI Appendix*, *Chemical Synthesis* and Table S2) also yielded essentially the same cocrystal structure. Examination of potential X-ray-induced global and local radiation damages within the diffraction dataset did not yield any clear sign of radiation damage during data collection. Molecular dynamics simulation of the obtained structure revealed that the chlorine atom exhibits constant movement away from the horizontal and vertical axes of the compound (Movie S1), thus in part explaining the poor detection of this atom in the diffraction data. Notably, 5-chloro-thiophene derivatives showed a significantly increased solubility and a higher antiproliferation activity than their respective dechlorinated form or other fused aromatic rings [compare **14** to **12** (*SI Appendix,* Fig. S1*B* and Table S1)]. Therefore, the chlorine-containing Allopole and its active form, Allopole-A, were used for further biochemical and cell biology analyses.

Comparative structural analyses revealed that the L2 loop, which runs near the canonical phospho-binding cleft, is highly flexible in the absence of a bound phospholigand (*SI Appendix,* Fig. S2*D*) but is attracted to the cleft through multiple water-mediated interactions with the ligand anchoring to the phospho-binding pocket (Fig. 2 *C* and *D*, *Left*). Analysis with overlaid crystal structures showed that the L2 loop recruited to a phospholigand-bound PBD1 traverses over the W-F pocket and is not compatible with the Allopole binding to the pocket (Fig. 2*C*). Unsurprisingly, binding of Allopole-A to the W-F pocket displaced the L2 loop by sterically inhibiting the I497 binding to the W-F pocket and altering the hydrogen (H)- bonding capability of W410 and P407 with L2 loop backbone atoms (*SI Appendix,* Fig. S2*B*). As a consequence, the intermolecular H-bond network of the L2 loop (*SI Appendix,* Fig. S2*B*) was not able to form and organize the ordered water-mediated interactions between the loop and a phospholigand (Fig. 2 *D*, *Right* and *SI Appendix,* Fig. S2*E*). This led to destabilizing the phospholigand bound to PBD1. Thus, Allopole-A antagonizes the PBD1-dependent interaction with its phospholigands by “unstrapping” the L2 loop critical for securing a bound target.

In a related experiment, we examined whether Allopole-A binding alters His-Plk1 kinase activity in vitro. Remarkably, the provision of Allopole-A significantly inhibited Plk1 activity, whereas the provision of a phospho-13mer (i.e., p-13mer; CETFDPPLHSpTAI) ligand derived from the PBIP1 p-T78 motif (49) increased it (Fig. 2*E* and *SI* *Appendix,* Fig. S2*F*). This suggests that the displacement of the L2 loop by Allopole-A renders the Plk1 kinase domain (KD) susceptible to PBD-dependent inhibition. This is consistent with the earlier observation that apo-PBD with the displaced L2 loop (*SI Appendix,* Fig. S2*D*) inhibits the Plk1’s catalytic activity through the KD–PBD interaction (50, 51). Activation of Plk1’s catalytic activity by phospholigand binding (14), which induces the strapped L2 loop state (Fig. 2*C*) and disrupts the KD–PBD1 interaction (51), is well documented.

### Both F559 and W410 Appear to Be Critical for Allopole-A’s Plk1-Binding Specificity.

**Fig. 3.**
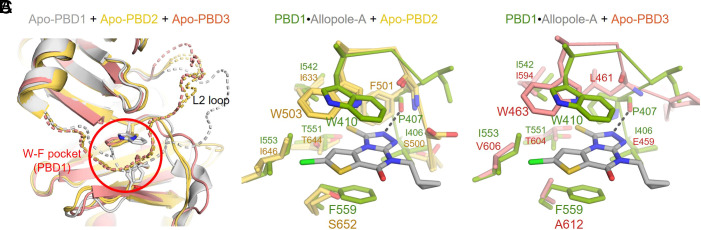
Determinants of the specificity of Allopole-A-binding to PBD1. (*A*) Overlays of Apo-PBD1 (PDB 3HIH), Apo-PBD2 (PDBs 4XB0 and ARS6), and Apo-PBD3 (AlphaFold Protein Structure Database) structures. Broken lines indicate flexible L2 loops for PBD1–3. (*B* and *C*) Overlays of the PBD2 (PDB 4XB0) (*B*) or a predicted PBD3 (AlphaFold) (*C*) structure with the PBD1•Allopole-A structure to show the oppositely oriented indole ring of W503 or W463, respectively, and the presence of S652 or A612, respectively, at positions analogous to the W410 and F559 of PBD1. Note that Allopole-A is sandwiched between the W410 and F559 residues of PBD1 (PDB 8CRC). In addition, the P407 residue, which is uniquely found in PBD1, forms an H-bond with Allopole-A (black dashed lines in *B* and *C*).

Overlaid structures of apo-PBD1 with that of apo-PBD2 (52, 53) and apo-PBD3 (AlphaFold Protein Structure database, https://alphafold.ebi.ac.uk) revealed that their overall folds are similar, with all showing a flexible L2 loop around the Allopole-A-binding pocket (Fig. 3*A*). To offer an insight into how Allopole-A achieves its superb specificity against PBD1 (*SI Appendix,* Fig. S1*F* and Table S3), we closely inspected the structure of the PBD1•Allopole-A complex in comparison to that of apo-PBD2 and apo-PBD3. One prominent difference was that at the position analogous to the Plk1 F559 residue, Plk2 and Plk3 possessed a Ser and an Ala residue (S652 and A612, respectively) (*SI Appendix,* Fig. S3*A*), neither of which would support the π–π interactions with Allopole-A (Fig. 3). In addition, although Plk1 W410 is an evolutionarily conserved residue, with analogous residues found in PBD2 and PBD3 (W503 and W463, respectively), the indole ring of W410 in Plk1 was oriented in a direction opposite to that of PBD2 and PBD3, thus ensuring another layer of the π–π interaction with Allopole-A (Fig. 3). These findings suggest that both W410 and F559 residues are critical not only for establishing π–π interactions with Allopole-A (Fig. 2 *A* and *B*) but also for achieving their binding specificity.

The vital role of the PBD1 F559 residue in binding Allopole-A hints that the introduction of a Phe residue in place of the PBD2 S652 or PBD3 A612 residue could render it sensitive to Allopole-A treatment. However, both PBD2 S652F and PBD3 A612F mutants were not inhibitable by Allopole-A (*SI Appendix,* Fig. S3 *B*–*D*). This suggests that in addition to W410 and F559 residues playing a pivotal role in Allopole-A binding, other residues constituting the W-F pocket (shown in Fig. 2*B*) are required for this event.

### Mutation of Plk1 F559 Confers Resistance to Allopole Treatment.

**Fig. 4.**
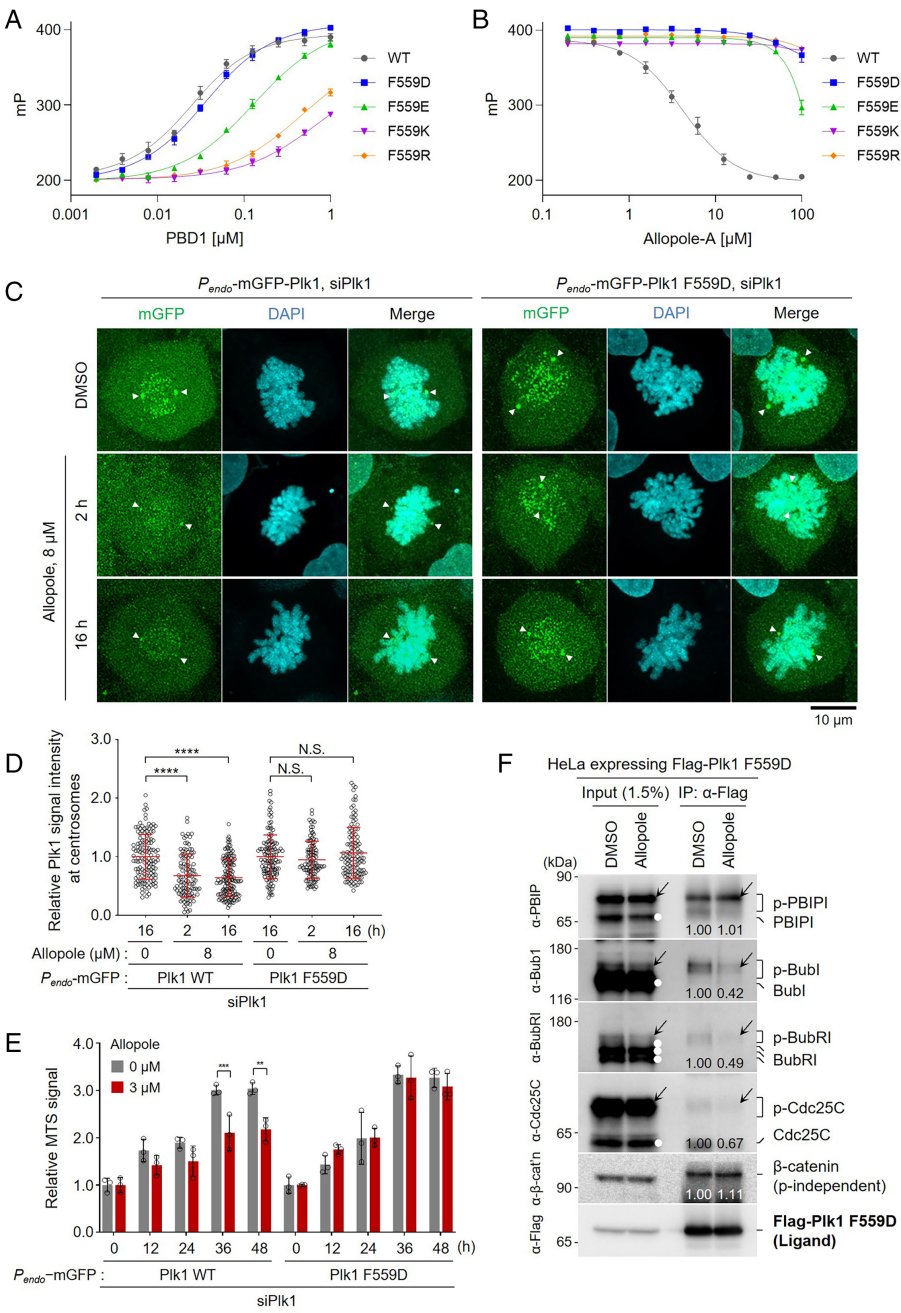
Mutation of F559 to a charged residue renders Plk1 insensitive to Allopole treatment in vitro and in cells. (*A*) FP-based assays showing the binding of FITC-Ahx-DPPLHSpTAI-NH_2_ ((36)) to the different concentrations of WT or mutant forms of PBD1. Bars, mean ± SD. K_d_ values extrapolated from the binding curves are shown in *SI Appendix,* Fig. S4*B*. (*B*) FP-based inhibition assays for the interaction between FITC-Ahx-DPPLHSpTAINH_2_ (15 nM) and the EC_85_ concentration of the indicated PBD1 WT or mutants (*SI Appendix,* Fig. S4*B*) in the presence of various concentrations of Allopole-A. Bars, mean ± SD. (*C* and *D*) Confocal imaging (*C*) and quantification (*D*) of Plk1 endogenous promoter (*P_endo_*)-fused mGFP-Plk1 signals in U2OS cells silenced for endogenous Plk1 (siPlk1) and treated with 8 µM of Allopole for the indicated length of time. An immunoblot showing the levels of Plk1 WT and the F559D mutant and an experimental schedule for (*C* and *D*) are provided in *SI Appendix,* Fig. S4 *C* and *D*, respectively. Arrowheads, centrosomes. Quantification of *P_endo_*-mGFP-Plk1 signals at centrosomes was performed with the images acquired from three independent experiments [n = a total of 93, 53, and 79 cells for Plk1 WT samples (in order) and n = a total of 92, 64, and 76 cells for the F559D mutant samples (in order)]. Bars, mean ± SD. ****P < 0.0001; N.S., not significant (*P* > 0.05) (unpaired two-tailed *t* test). (*E*) MTS-based cell proliferation assays were carried out using the cells in *C* and *D* seeded at approximately 5,000 cells/well in a 96-well plate. An experimental schedule for *E* is provided in *SI Appendix,* Fig. S4*E*. At the 48-h time point, cells reached a plateau phase in growth because of their saturated density. **P < 0.01, ***P < 0.001 (unpaired two-tailed *t* test). Bars, mean of three experiments ± SD. (*F*) Coimmunoprecipitation (IP) and immunoblotting analyses were carried out as shown in Fig. 1*C* except that HeLa cells stably expressing the Flag-Plk1 F559D mutant were used. Both phosphorylated (arrows) and un/underphosphorylated (white dots) species for each protein are indicated. β-catenin is reported to bind to PBD1 in a phosphorylation-independent manner (33), thus serving as a control. Numbers, relative signal intensities.

Since Allopole-A binding to PBD1 is primarily mediated by the F559 and W410 residues, we first mutated F559 to either positively or negatively charged residues to disrupt the highly hydrophobic W-F pocket (Fig. 2 *A* and *B*). In FP-based assays carried out with recombinant PBD1 proteins (*SI Appendix,* Fig. S4*A*), the F559D mutant protein bound to an FITC-conjugated phosphopeptide (i.e., FITC-Ahx-DPPLHSpTAI-NH_2_) (15, 49) with a slightly diminished (1.7-fold) affinity when compared to WT, while the F559E mutant showed an approximately sevenfold decreased affinity (Fig. 4*A* and *SI Appendix,* Fig. S4*B*). Under the same conditions, both F559K and F559R mutants showed more than 27-fold and 42-fold, respectively, lower affinities than that of WT. The PBD1 binding inhibition assays were performed at an effective concentration equal to 85% of the maximal concentration (i.e., EC_85_) determined for WT or each mutant protein (*SI Appendix,* Fig. S4*B*). Under these conditions, while Allopole-A effectively inhibited PBD1 WT, it failed to inhibit any of the four mutants (Fig. 4*B*), suggesting that these mutants were deprived of the Allopole-A-binding capacity. Mutation of the evolutionarily conserved W410 to Ala or Tyr residue appeared to impair the capacity of PBD1 to bind the FITC-conjugated ligand, preventing us from further analyzing the consequence of this mutation. Thus, we revealed that W410 could be important for the structural integrity of the protein.

To corroborate the importance of the F559 residue for Allopole-A binding, U2OS cells expressing endogenous promoter (i.e., *P_endo_*)-controlled mGFP-Plk1 WT or F559D mutant were depleted of endogenous Plk1 by RNAi for 48 h (*SI Appendix,* Fig. S4*C*), treated with control DMSO or 8 μM of Allopole for 2 or 16 h, and fixed (*SI Appendix,* Fig. S4*D*). Afterward, the level of mGFP fluorescence was quantified using confocal microscopy (Fig. 4 *C* and *D*). As expected from the results shown in Fig. 1 *C* and *D*, Allopole delocalized mGFP-Plk1 WT from centrosomes within 2 h after treatment, and this effect was sustained for up to 16 h. In contrast, Allopole failed to alter the level of the centrosome-localized mGFP-Plk1 containing the F559D mutation under the same conditions (Fig. 4 *C* and *D*). In a related experiment, Plk1 RNAi cells expressing WT Plk1 showed a significant reduction in their proliferation rate at 36 h when treated with 3 to 6 μM of Allopole (Data obtained with 3 μM of Allopole is shown in Fig. 4*E* and *SI Appendix,* Fig. S4*E*). Expectedly, the cells expressing the Plk1 F559D mutant exhibited uninhibited proliferation under the same conditions. Furthermore, Unlike Plk1 WT in Fig. 1*C*, the F559D mutant coimmunoprecipitated p-PBIP1, p-Bub1, p-BubR1, and p-Cdc25C effectively even in the presence of 20 μM of Allopole (Fig. 4*F*), confirming that the F559 residue is required for Allopole-Abinding. Notably, while the interaction with p-PBIP1 remained unchanged, those with p-Bub1, p-BubR1, and p-Cdc25C were somewhat diminished (42 to 67% level) (Fig. 4*F*). This could be attributable to the fact that unlike PBIP1, which binds to PBD1 through the self-priming and binding mechanism (49), Bub1, BubR1, and likely Cdc25C require Cdc2-dependent priming phosphorylation to interact with PBD1 (42–44). Allopole treatment could have decreased Cdc2 activity through a positive-feedback loop (54), as evidenced by the reduced levels of slow-migrating forms (arrows in the Input for Bub1 and BubR1) (Fig. 4*F*).

## Discussion

**Fig. 5.**
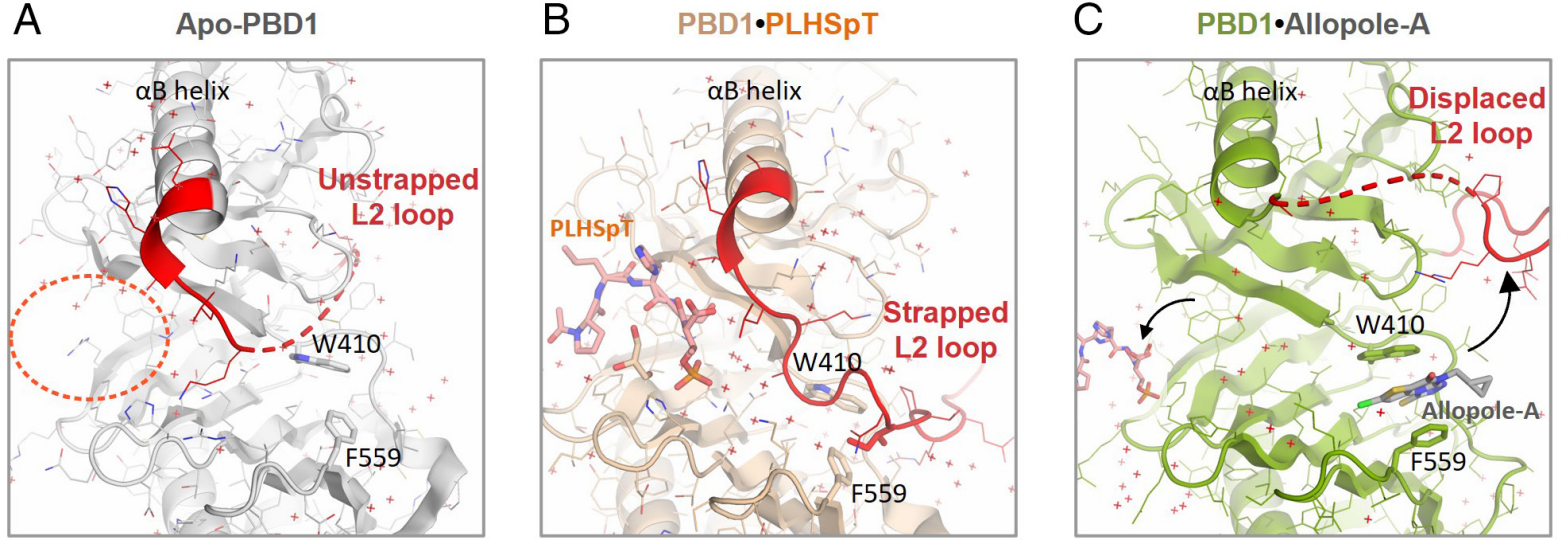
Model illustrating how Allopole-A inhibits the PBD1–phospholigand interaction. In a ligand-unbound state, the L2 loop is loosely placed (depicted by the broken line) near the canonical phosphoepitope-binding cleft (*A*, dotted circle). As a phospholigand (i.e., PLHSpT) anchors to the binding cleft, the L2 loop secures the phospholigand binding through a layer of water network–mediated interactions and additional H-bonds (*B*, *SI Appendix,* Fig. S2*E* for details). This event, which brings the L2 loop to strap around the binding cleft, occludes the allosteric W-F pocket. Allopole-A’s binding to the W-F pocket displaces the L2 loop to the magnitude that all the L2-mediated interactions are stripped from the phospholigand (*C*). The unstrapping of the L2 loop by Allopole-A triggers the dissociation of the phospholigand from the PBD1. Apo-PBD1, PBD1•PLHSpT, and PBD1•Allopole-A structures were generated from PDBs 1Q4O, 3HIK, and 8CRC, respectively. Broken lines indicate the flexible region undetectable in the crystal structures.

As of today, efforts to target the canonical phospholigand-binding cleft (14) have yielded only limited success. This could be due to multiple reasons, including 1) the shallow phosphoepitope-binding cleft, 2) the requirement of a negatively charged moiety mimicking the p-S/T high-affinity anchor, and 3) the difficulty of satisfying the water network–mediated interactions required for securing the phosphoepitope–PBD1 interactions. In this regard, the generation of Allopole and the discovery of its unique binding mode to a deep allosteric pocket (Fig. 2) provide unprecedented opportunities for anti-Plk1 drug discovery. How Allopole-A inhibits the interaction between PBD1 and its cognate binding targets is elucidated at the molecular level. Interestingly, the binding of a phospholigand to the canonical binding site attracts the highly flexible L2 loop (*SI Appendix,* Fig. S2*D*) to a shallow groove and establishes multiple water-mediated interactions (e.g., seven water molecules in the case of PLHSpT) with the L2 loop (Fig. 5, *Left* and *Middle* and Movie S2). Allopole-A binding to the allosteric W-F pocket occluded by the L2 loop dislodges the loop, consequently stripping several water-mediated interactions critical for phosphoepitope binding (Fig. 5, *Right* and Movie S2). These findings suggest that the L2 loop functions to secure the phospholigand anchored to the phosphoepitope-binding cleft and that unstrapping of the L2 loop by Allopole-A is sufficient to trigger the dissociation of a phospholigand and to inhibit PBD1-dependent downstream events.

The high level of specificity that Allopole-A offers is not surprising, given that allosteric modulators are expected to offer greater degree of selectivity over orthosteric inhibitors, as demonstrated with allosteric ligands targeting GPCRs (55, 56). Remarkably, the W-F pocket is surrounded by several hydrophobic residues (I406, P407, W410, I542, I553, and F559) forming a uniquely definable space (Fig. 2), a feature that makes it distinguishable from the somewhat nondescript phospho-binding cleft evolved to interact with numerous PBD1-binding targets. In line with this observation, potential off-target interactions for Allopole, Allopole-A, and related compounds showed less than 50% inhibition at 10 μM for nearly all screened 45 receptors and channels by the National Institute of Mental Health Psychoactive Drug Screening Program (https://pdsp.unc.edu/pdspweb/) (*SI Appendix,* Table S5). (Compare with Allopole-A exhibiting an IC_50_ value of 2.53 nM against Plk1 PBD in vitro, *SI Appendix,* Table S3.) Allopole showed only two weak µM interactions, and Allopole-A showed no interactions. This is in sharp contrast to the previously characterized Poloxin (18) and its related inhibitors (i.e., Thymoquinone and Poloxime), which are proposed to bind to the canonical phosphoepitope-binding site (57) and are considered protein alkylators, causing nonspecific cytotoxic effects in vivo (5, 26). In addition, the more recently developed Polotyrin, which is reported to bind to the Tyr-enriched hydrophobic channel (35, 58) of the canonical site, exhibits poor potency (GI_50_ value of a few hundred μM) (21). Therefore, in light of current challenges in targeting the canonical phosphoepitope-binding site, the discovery of an allosteric pocket and the distinct mode of Allopole-A binding would significantly change the future direction of developing anti-PBD1 inhibitors.

Prodrugs have been used effectively to overcome deficiencies in the physicochemical properties of biologically active agents (59). In this study, we used the 5-thio-1-methyl-4-nitroimidazolyl moiety, which has been used for the orally active anti-inflammatory drug azathioprine (60), to render Allopole-A to cross the cell-membrane barrier. Upon entering into the cell, the prodrug moiety appears to be liberated rapidly, judging from the complete conversion of a prodrug into an active species in less than 5 min in the presence of the intracellular concentration of GSH (*SI Appendix,* Fig. S1*H* and Table S2). However, considering a low micromolar range of cellular potency (GI_50_ value of 4.1 μM), further optimization of Allopole appears necessary not only to improve its cellular efficacy but also to provide physicochemical and metabolic properties suitable for preclinical and clinical studies. The cocrystal structure of the PBD1•Allopole-A complex would greatly facilitate the rational design and development of a new class of PBD1-specific anticancer therapeutic agents.

## Materials and Methods

### Chemical Synthesis.

Chemical synthesis of Allopole and its related compounds, biological analyses, and other experimental methods, including X-ray crystallography, dynamic light scattering, and liquid chromatography–mass spectrometry (LC-MS), are described in *SI Appendix*.

### ELISA-Based PBD1-Binding Inhibition Assay.

The assay, which is designed to determine the ability of a compound to inhibit the interaction between the full-length Plk1 and a biotinylated PBIP1 phospho-T78 peptide (i.e., Biotin-Ahx-CETFDPPLHSpTAI-NH_2_) (15, 49), was performed essentially as described previously (15). The source of the full-length Plk1 was the total lysates prepared from HEK293A cells infected with an adenovirus expressing human influenza hemagglutinin (HA) and EGFP-fused Plk1. Data are reported as IC_50_ to indicate the concentration producing 50% inhibition of the phosphopeptide binding. The reaction products were measured at 450 nm by using a Perkin-Elmer EnSpire Multimode Plate reader (PerkinElmer, Inc.). All the data were obtained from more than three independent experiments and were analyzed by GraphPad Prism software version 8.

### PBD1–3 FP Binding Assays for Plk1 Specificity.

FP assays were carried out essentially as described previously (35, 36). FITC-conjugated ligands (15 nM) were mixed with PBDs at their EC_85_ concentrations. All the samples were analyzed approximately 30 min after reaction in a 384-well format using the SpectraMax Paradigm multimode microplate detection platform (Molecular Devices). Data were analyzed after carrying out at least three independent experiments using GraphPad Prism software version 8.

### Cell Culture, mGFP-Plk1-Expressing Cell Generation, siRNA Treatment, and Lentivirus Production.

HeLa and U2OS cells were cultured as recommended by the American Type Culture Collection. Multiple myeloma–derived L363 cells were cultured as reported previously (34). To generate U2OS cells expressing *P_endo_*-mGFP-Plk1 WT and the F559D mutant used for Fig. 4 *C*–*E*, the cells were infected with the lentivirus expressing the respective gene for 1 d and then selected with 2 μg/mL of puromycin (Sigma-Aldrich). To avoid unintended overexpression, cells were infected with the level of lentiviruses showing approximately 90% survival after puromycin selection. The resulting selected cells were then transfected with 20 nM of siRNA (AGATTGTGCCTAAGTCTCT; nt 245–263 of human Plk1) (61) to silence the expression of endogenous Plk1.

Lentiviruses expressing either mGFP-Plk1 WT or F559D under the control of endogenous Plk1 promoter (Fig. 4*C* and *SI Appendix,* Fig. S4*C*) were generated by cotransfecting pHR′.J-CMV-SV-puro-*P_endo_(−886 to −1)*-mGFP-Plk1 WT (pKM7889) or -Plk1 F559D (pKM7893) with pHR'-CMV∆R8.2∆vpr and pHR'-CMV-VSV-G as described previously (49). The pKM7889 and pKM7893 were generated by inserting a ClaI-AscI fragment containing *P_endo_*-mGFP and then inserting an AscI-PmeI fragment containing either Plk1 WT or F559D fragment. The lentiviral construct expressing a CMV promoter-controlled Flag-fused Plk1 (pKM3863) was generated by inserting an AscI (end-filled)-NotI fragment into the pHR′.J-CMV-SV-puro-based vector described previously (62). HeLa cells stably expressing Flag-Plk1 (Fig. 1*C*) or Flag-Plk1 F559D (Fig. 4*F*) were used for coimmunoprecipitation analysis.

### Time-Lapse Analysis.

To carry out time-lapse analysis, HeLa cells were arrested at the G1/S boundary by treating them with 2.5 mM of thymidine for 18 h, releasing them for 9 h, and then treating them again with the same dose of thymidine for another 18 h. The resulting cells were washed with fresh medium, released from the double-thymidine block for 7 h, and then treated with control DMSO or 10 μM of Allopole to examine their effect on cell cycle progression. At the indicated time points, cells were imaged every 30 min using a Keyence BZ-X710 all-in-one microscope.

### Immunostaining and Confocal Analysis.

HeLa cells treated with either control DMSO or on 10 μM of Allopole for 2 h were immunostained using anti-Plk1 (F-8, sc-17783, Santa Cruz Biotechnologies), anti-Cep192 (63), and anti-CREST (#15-234, Antibodies Incorporated) antibodies and appropriate secondary antibodies, essentially as described previously (62). Samples were additionally stained with 2 µg/mL of Hoechst 33342 to reveal chromosomal morphologies. Images were captured using a Zeiss LSM 780 confocal microscope (Carl Zeiss Microscopy, LLC) equipped with plan-apochromat 40× (N.A. 1.3) and 63× (N.A. 1.4) oil-immersion objective lenses; a 34-channel GaAsP spectral detector; and 12-bit, 0.5-μm z-steps.

### Quantification of Fluorescence Signal Intensities.

Confocal images of unsaturated fluorescence signals were acquired under the same laser intensity at the 12-bit resolution. Fluorescence intensities for identifiable centrosome and kinetochore signals were quantified after the maximum-intensity projection of z-stacks using the Zeiss ZEN v2.1 software (Carl Zeiss Microscopy, LLC). Data were quantified from three independent experiments.

### MTS Cell Viability Assay.

Cell viability was determined using the calorimetric CellTiter 96® AQ_ueous_ One Solution Cell Proliferation Assay (MTS) kit (Promega) containing a tetrazolium compound [3-(4,5-dimethylthiazol-2-yl)-5-(3-carboxymethoxy-phenyl)-2-(4-sulfophenyl)-2H-tetrazolium, inner salt (MTS)] and an electron-coupling phenazine ethosulfate. For the MTS assays in Fig. 1*B*, L363 cells cultured in a 96-well plate were treated with the indicated compounds for 48 h (the final volume of 100 μL). After the compound treatment, cells were additionally treated with 20 μL of the MTS solution and incubated at 37 °C for 2 to 4 h in a humidified 5% CO_2_ atmosphere. The absorbance of the resulting sample was measured at 490 nm using a PerkinElmer EnSpire Multimode 96-well plate reader. For the MTS assays in Fig. 4*E*, U2OS cells stably expressing either *P_endo_*-mGFP-Plk1 WT or F559D were seeded at approximately 5,000 cells/well in a 96-well plate, depleted of endogenous Plk1 by RNAi, and then treated with either control DMSO or Allopole as indicated in the schematic diagram in *SI Appendix,* Fig. S4*E*.

### Immunoprecipitation and Immunoblotting Analyses.

For coimmunoprecipitation analyses shown in Figs. 1*C* and 4*F*, HeLa cells stably expressing *P_CMV_*-Flag-Plk1 WT (pKM3863) (Fig. 1*C*) and its respective F559D mutant (pKM7979) (Fig. 4*F*) were generated using a lentiviral expression system. Prior to harvest, the cells were treated with 600 nM of nocodazole and 300 nM of okadaic acid (OA) for 16 h and 1.5 h, respectively, to enrich the level of phosphorylated proteins and additionally treated with either control DMSO or 20 μM of Allopole for 2 h. Immunoprecipitation was carried out essentially as described previously (49) in TBSN buffer [20 mM Tris–Cl (pH 8.0), 150 mM NaCl, 0.5% NP-40, 5 mM EGTA, 2 mM MgCl_2_, 1.5 mM EDTA, 2 mM DTT, 20 mM *p*-nitrophenyl phosphate (PNPP), and protease inhibitor cocktail (Roche)] using anti-Flag antibody (Sigma-Aldrich). Immunoprecipitates were separated by sodium dodecyl sulfate–polyacrylamide gel electrophoresis (SDS-PAGE) and then immunoblotted according to standard procedures using an enhanced chemiluminescence detection system (Thermo Fisher Scientific). The antibodies that were used to detect specific indicated proteins in Fig. 1*C* are listed in *SI Appendix,* Table S6.

### In Vitro Plk1 Kinase Assay.

Kinase reactions were carried out in two steps. First, Sf9-purified 0.2 µg of human His-Plk1 (Sino Biological) was preincubated in a kinase cocktail [50 mM Tris-Cl (pH 7.5), 10 mM MgCl_2_, 2 mM dithiothreitol, 2 mM EGTA, and 27 mM *p*-nitrophenylphosphate] in the presence of either Allopole-A or p-13mer PBIP peptide for 20 min at 25 °C. The samples were then added with 1 µg of dephosphorylated casein (Sigma) and 1.25 µM of ATP (1.25 µCi of [γ^32^P] ATP, 1Ci = 37 GBq) and reacted for additional 30 min at 30 °C. The reactions were terminated by the addition of 5× Laemmli SDS sample buffer, separated by 10% SDS-PAGE, and analyzed by autoradiography. To quantify the γ^32^P signals, appropriate bands were excised from dried SDS-PAGE gels and analyzed by a liquid scintillation counter (Beckman model LS 6500).

### Pulldown Analysis with Biotinylated Compound 22.

A mixture of total cell lysates expressing Flag-Plk1 K82M (pKL577), Flag-Plk2 K108M (pKL1416), or Flag-Plk3 K52R (pKM203) (a gift from Wei Dai, New York University School of Medicine, NY) was prepared in TBSN buffer and incubated with a biotinylated Allopole-A-derivative, **22**, or biotin alone. The resulting samples were then subjected to pulldown analyses with streptavidin agarose bead (Thermo Fisher Scientific), after which they were separated by SDS-PAGE and immunoblotted with anti-Flag antibody.

### Protein Expression and Purification.

For X-ray crystallography, the His-MBP-TEV-PBD1 (residues 371-594) (pKM5791) construct, which is generated by inserting an NdeI-XhoI fragment into a His-MBP-Tobacco Etch Virus (TEV) vector (pKM2624), was expressed in *Escherichia coli* Rosetta strain (Novagen). Proteins were purified essentially as described previously (15). In brief, cultured cells were lysed in ice cold Buffer A containing 20 mM Tris–HCl (pH 8.0), 600 mM NaCl, 5% v/v glycerol, and 0.5 mM *2*-*carboxyethyl phosphine* (TCEP). The resulting lysates were loaded onto a to His Trap HP column (GE Healthcare) and eluted in a stepwise manner using increasing concentrations of imidazole with Buffer A. The eluted fractions were then concentrated and subjected to further purification through size-exclusion chromatography using a HiLoad 16/600 Superdex 200 column (GE Healthcare). After His-trap purification, the 6xHis tag was cleaved by TEV protease at 4 °C overnight. To remove the TEV protease and 6xHis tag from the solution, the sample was subjected to reverse His-trap purification, followed by size-exclusion chromatography. The final purified protein was stored in the buffer containing 20 mM Tris–HCl (pH 8.0), 500 mM NaCl, 5% v/v glycerol, and 1 mM EDTA at −70 °C until further use.

For FP assays, constructs expressing PBD1 (residues 345–603) WT (pKM7650), F559D (pKM7878), F559E (pKM7879), F559K (pKM7880), or F559R (pKM7881) were generated by inserting a respective EcoRI-XhoI fragment of TEV-PBD1 into the pET-Duet-1 vector (Addgene) digested with the same enzymes. Proteins were purified as described above. His-affinity-purified proteins were used for analysis. Purification of PBD2-His (355–685) (pKM3358) and its S652F mutant (pKM7980) and MBP-PBD3-His (335–646) (pKM3359) and its A612F (pKM7981) was performed as for PBD1 purification. PBD3 could be purified only in an MBP-fused form.

### Crystallization Conditions.

Crystals were grown using the hanging drop vapor diffusion method. PBD1 protein at 12 mg/mL in 20 mM Tris (pH 8.0), 0.5 M NaCl, 1 mM EDTA, 2% DMSO, and 2 mM Allopole-A was mixed with an equal volume of a precipitant solution consisted of 11 to 15% (w/v) PEG 3350 and 0.1 M MES (pH 6.0). Reservoir solution contained 1.25 M NaCl. Crystals appeared overnight at room temperature and reached maximum size over several days. Crystals were cryoprotected in the precipitant solution supplemented with 20% glycerol for further processing.

### Data Collection and Structure Determination and Refinement.

Diffraction datasets were collected at 100 K on the 22-ID beamline of the Advanced Photon Source, Argonne National Laboratory, Lemont, IL. The datasets were indexed, integrated with the XDS software package (64), and scaled with Aimless from CCP4 software suite (65). The structure was solved by the molecular replacement method using PHASER (66). The crystal structure of CPB (PDB 3HIK chain A) was used as a search model. Structure refinement (1.65 Å resolution) was carried out with phenix.refine in the Phenix suite (67) and manual fitting in Coot (68). Allopole-A structure and restraints file were generated by phenix.elbow (69). Data collection and refinement statistics are summarized in *SI Appendix,* Table S4. The figures were created using PyMOL (http://www.pymol.org/).

### Molecular Dynamics Simulation.

The PBD-NCK189 simulation system was built with CHARMM-GUI (70) by using the crystal structure of the PBD-NCK189, including an adjacent protein symmetry mate that lies within 4 Å. Force field parameters were generated by the CHARMM36m additive force field for both the protein and the NCK189 compound. The simulation system was first energy-minimized by the GROMACS 2022.3 simulation package (https://doi.org/10.5281/zenodo.7037337) using the 5,000-step steepest descent method, followed by a 25 ps NVT equilibration with position restraints on the protein heavy atoms. In the equilibration simulation, a 1 fs timestep was used, and the temperature was maintained at 303.15 K by means of a Nosé–Hoover thermostat (71, 72).

The protein-compound system was produced in a 200 ns simulation with the restraints still placed on protein heavy atoms. In production simulation, a 2 fs timestep was used, the temperature was kept at 303.15 K with a Nosé–Hoover thermostat, and the pressure was regulated by using a stochastic cell-rescaling barostat (73). The simulation trajectory was visualized and stored in a video format using the Visual Molecular Dynamics software package (VMD 1.9.4) (74).

### Allopole Conformer Generation and VDW Clash Studies.

Conformers for Allopole prodrug were generated by the RDKit package with rdk_confgen.py script using the MMFF94s force field. Mol file of Allopole was provided as input and the output.sdf file contained 50 conformers with the lowest energies. The conformers were manually clustered based on the rotameric state (producing 8 different clusters), and a representative molecule from each cluster was tested for VDW clashes in Pymol using the show_bump.py Python module.

### Dynamic Light Scattering.

All samples were analyzed after adjusting the concentration to 100 μM solution in the PBS buffer (pH 7.4) containing 1% DMSO. The measurements were carried out at 25 °C using the DynaPro NanoStar system (Wyatt Technology). The intensity of the scattered light was measured as a function of time, and the resulting data were used to calculate the size and distribution of the particles in the sample. Collected data were analyzed using the DYNAMICS software to calculate the mean size and polydispersity index of the particles in the sample. At least three repeat measurements were performed to ensure that the results were accurate and reproducible.

### LC-MS and Data Processing.

High-performance liquid chromatography (HPLC)/high-resolution mass spectrometry was used to determine the products derived from investigational drugs incubated under various conditions by measuring their exact masses. Thereafter, 20 μL aliquots of several drug preparations in either DMSO or aqueous reaction solutions were diluted 2:1 in ethanol, followed by centrifugation at 15,000 × *g* for 5 min. The supernatant was then injected on a Thermo Vanquish liquid chromatograph coupled to a Thermo Q Exactive Orbitrap mass spectrometer (Thermo Fisher Scientific). The conditions for the analysis are listed as follows.

#### Analytical column.

Phenomenex Kinetex C8, 5 μm, 100 A, 100 mm × 2.1 mm maintained at 40 °C.

#### Mobile phase.

Solvent A, 0.1% formic acid in water/solvent B, 0.1% formic acid in acetonitrile.

#### Gradient.

20% B for 3 min, then nonlinear programmed to 75% B in 3 min, then held for 4 min, for a total run time of 10 min.

#### Flow rate.

300 μL/min.

#### Injection volume.

3.0 μL.

#### Ionization source.

Positive electrospray optimized for 300 μL/min flow rate.

#### Acquisition mode.

Full Scan/SIM, mass resolution 70,000, max ion injection time 200 μs ACG target of 3E6, mass range of 50 to 750 m/z.

The analytes were identified using the Dmass between the predicted and measured exact monoisotopic mass for the protonated molecular ions applied to the empirical formula algorithm in the Qual Browser application of the Xcalibur software (Themo Scientific). Selected ions from the base peak chromatogram were extracted for integration of peak areas. The relative amounts of each analyte to the sum total were calculated to estimate conversion of and prodrug under the specific conditions to breakdown products.

### HPLC and Detection of Prodrug and Nonprodrug (Active) Species.

To a solution of **6** (1.0 mg, 2.4 µmol) in 0.800 mL of MeOH-d_4_ was added a solution of glutathione (1.1 mg, 3.6 µmol) and sodium carbonate (0.76 mg, 7.2 µmol) in 0.200 mL of D_2_O. The reaction was stirred at room temperature and sampled at 5, 10, 20, and 60 min. Aliquots were flash-frozen on dry ice and always stored at −80 °C. HPLC and mass spectrometry were used to analyze the reaction mixtures.

#### Allopole-GSH Detection.

HRMS m/z (M+H) for C_14_H_20_N_6_O_8_S calcd, 433.1142; exact mass found, 433.1136 (*SI Appendix,* Fig. S1*G*).

#### Conversion of Prodrug 6 to Active Species 5 by GSH.

HPLC analysis of both pure **6** (rt = 13.95 min) and **5** (rt = 11.16 min) using the method of acetonitrile 05 to 95% and 10 mM triethylammonium acetate 95 to 5% in 20 min gave standards to compare against the reaction mixture. The HPLC analysis of the reaction mixture, compared to standards, was performed using a Hewlett−Packard 1100 HPLC equipped with an Agilent Eclipse 5 µm XDB-C18 analytical column (50 mm × 4.6 mm; Agilent Technologies Inc.). The chromatogram of the reaction mixture after 5 min shows complete conversion of **6** to **5** (*SI Appendix,* Fig. S1*H*).

## Supplementary Material

Appendix 01 (PDF)

Movie S1. Molecular dynamics simulation showing the flexibility of the chloride moiety of Allopole-A cocrystallized with PBD1. To mimic the crystallized state of the PBD1•Allopole-A complex (PDB 8CRC), simulation was performed after restraining the positions of the PBD1 residues in the presence of a symmetry mate (see Methods for details). The movement of the chloride moiety (colored lines) was tracked during the entire length of time (200 ns).

Movie S2. The mechanism underlying how Allopole-A inhibits the PBD1•phospholigand interaction. See Fig. 5 for details. Apo-PBD1, PBD1•PLHSpT, and PBD1•Allopole-A structures were modeled using PDBs 1Q4O, 3HIK, and 8CRC, respectively. See Fig. 5 for details.

## Data Availability

The detailed experimental procedure for chemical synthesis, copies of nuclear magnetic resonance spectra, LC-MS analyses, HPLC traces, X-ray crystallography refinement data, computational analyses, and any other information relevant to this study are available in the *SI Appendix*.
