# Supplementary material for: Specific inhibition of an anticancer target, polo-like kinase 1, by allosterically dismantling its mechanism of substrate recognition
Source: Proc Natl Acad Sci U S A. 2023 Aug 21;120(35):e2305037120. doi: 10.1073/pnas.2305037120 (PMC10629583; doi:10.1073/pnas.2305037120)
Supplement: Supplementary file 1 — Appendix 01 (PDF) [file pnas.2305037120.sapp.pdf]

Supporting Information for

Specific inhibition of an anticancer target, polo-like kinase 1, by allosterically dismantling its mechanism of substrate recognition

Jung-Eun Park[1+], Klara Kirsch[1+], Hobin Lee[1,2+], Paola Oliva[2], Jong Il Ahn[1], Harsha Ravishankar[1], Yan Zeng[1], Stephen D. Fox[3], Samuel A. Kirby[1,2], Pooja Badhwar[1], Thorkell Andresson[3], Kenneth A. Jacobson[2]*, and Kyung S. Lee[1]*

Kyung S. Lee
Email: kyunglee@mail.nih.gov

This PDF file includes:

Supporting text
Figures S1 to S4
Tables S1 to S6
Legends for Movies S1 to S2
SI References

Other supporting materials for this manuscript include the following:

Movies S1 to S2

Supporting Information Text

Materials and Methods

Chemical synthesis

Scheme S1. Synthesis of diverse heterocyclic active drugs.[a]

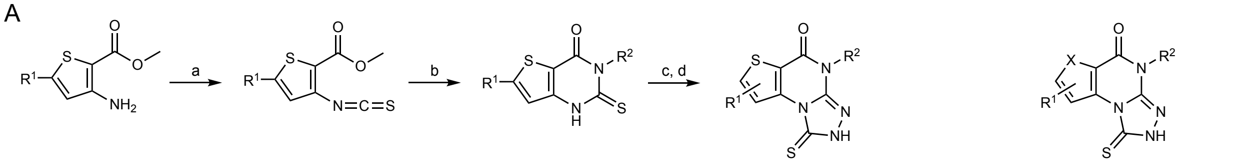

23, R = H
24, R = CH_3
25, R = Cl
26, R = t-Bu

27, R = H
28, R = CH_3
29, R = Cl
30, R = t-Bu

31, R = H
32, R = CH_3
33, R = Cl
34, R = t-Bu

3, R^1 = 5-CH_3; R^2 = $(CH_2)_2CH_3$
5, R^1 = 5-CH_3; R^2 = CH_2-cPr
11, R^1 = H; R^2 = $(CH_2)_2CH_3$
13, R^1 = 5-Cl; R^2 = $(CH_2)_2CH_3$
15, R^1 = 5-$C(CH_3)_3$; R^2 = $(CH_2)_2CH_3$
19, R^1 = 5-Cl; R^2 = CH_2-cPr

7, X = O, R^1 = 4,5-fused Ph; R^2 = $(CH_2)_2CH_3$
9, X = O, R^1 = 4,5-fused 4-F-Ph; R^2 = $(CH_2)_2CH_3$
17, X = S, R^1 = 4,5-fused Ph; R^2 = $(CH_2)_2CH_3$

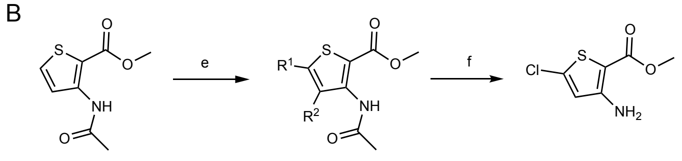

35

36, R^1 = Cl; R^2 = H
37, R^1 = Cl; R^2 = Cl

25

[a] Reagents and conditions: (a) thiophosgene, TEA, THF, room temperature, 1–18 h, crude; (b) (cyclopropyl)methyl amine or n-propylamine, THF, reflux, 18 h, 44–77%; (c) N_2H_4, EtOH, 80 ˚C, 18 h; (d) CS_2, KOH, EtOH, 80 ˚C, 18 h, 18–42%; (e) SO_2Cl_2, 50 °C, 2 h; (f) H_2SO_4, 100 °C, 6 h.

Scheme S1B shows the preparation of the starting material for Allopole-A **19** used in Scheme S1A. The chlorination of a commercially available *N*-acetylaminothiophene derivative **35** provided both monochloro, as the Allopole precursor **36**, and dichlorinated **37** products. Compound **36** was deacetylated as shown to provide intermediate **25**.

Scheme S2. Synthesis of *S*-aryl prodrugs of active drugs shown in Scheme S1.[a]

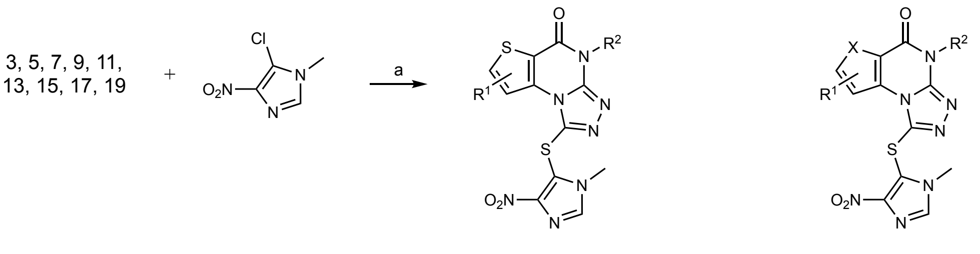

4, R^1 = 5-CH_3; R^2 = $(CH_2)_2CH_3$
6, R^1 = 5-CH_3; R^2 = CH_2-cPr
12, R^1 = H; R^2 = $(CH_2)_2CH_3$
14, R^1 = 5-Cl; R^2 = $(CH_2)_2CH_3$
16, R^1 = 5-$C(CH_3)_3$; R^2 = $(CH_2)_2CH_3$
20, R^1 = 5-Cl; R^2 = CH_2-cPr

8, X = O, R^1 = 4,5-fused Ph; R^2 = $(CH_2)_2CH_3$
10, X = O, R^1 = 4,5-fused 4-F-Ph; R^2 = $(CH_2)_2CH_3$
18, X = S, R^1 = 4,5-fused Ph; R^2 = $(CH_2)_2CH_3$

[a] Reagents and conditions: (a) thiophosgene, TEA, THF, room temperature, 1–18 h, crude; (b) (cyclopropyl)methyl amine or n-propylamine, THF, reflux, 18 h, 44–77%; (c) N_2H_4, EtOH, 80 ˚C, 18 h; (d) CS_2, KOH, EtOH, 80 ˚C, 18 h, 18–42%.

Scheme S3. Synthesis of biotin conjugate **22**.[a]

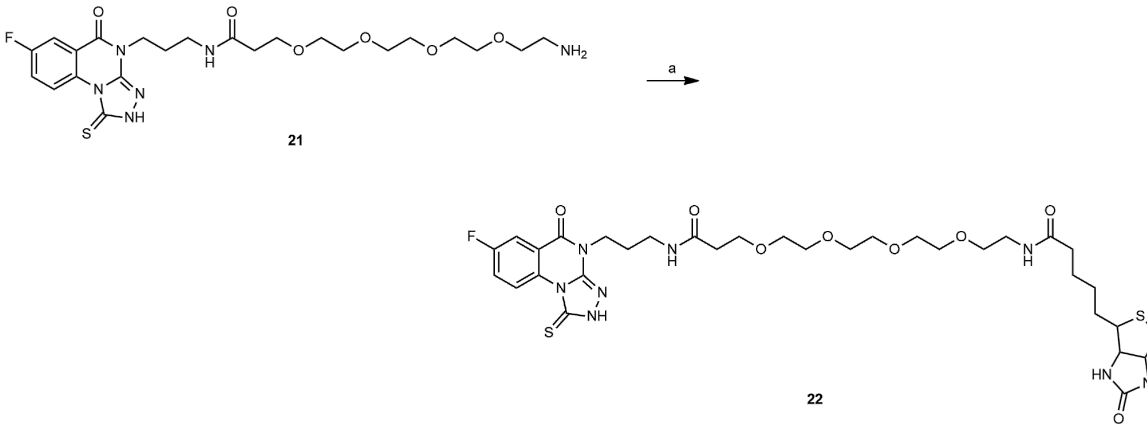

[a] Reagents and conditions: (a) biotin-Osu, TEA, DMF, overnight.

Reagents and instrumentation

The reactions were performed under anhydrous conditions under dry nitrogen. For particularly sensitive reactions, the glassware was oven-dried. Reactants used were reagent grade, and anhydrous solvents were purchased commercially. Compounds **3**, **4**, **7**, **9**, **11**, **13**, **15**, **17** and **21** were synthesized in a manner similar to that reported in (1) and (2). Room temperature is defined as 25 ± 2 °C. All of the final products for biological testing were purified using semi-preparative HPLC (Waters, Beverly, MA), using a Phenomenex Luna C18 (5 µm, 30 x 75 mm) column with a 45 mL/min flow rate. The mobile phase gradient (10% to 50% acetonitrile over 8 minutes) consisted of acetonitrile/water mixtures, with TFA (0.1%) present in all mixtures. The course of the HPLC purification was followed by UV at 220 nm. Selected compounds were initially purified following dry-loading on an ISCO CombiFlash System (Teledyne, Lincoln, NE) prior to HPLC. Fractions obtained during the synthesis were examined for expected product using an Agilent 1200 LC-MS (Agilent Technologies, Santa Clara, CA) with a rapid gradient consisting of 4% to 100% CH_3CN in water over 3 minutes, with a total separation time of 8 minutes (1 mL/min). The CH_3CN component contained 0.025% TFA, and the aqueous component contained 0.05% TFA. The purity of the final products (>95% at 254 nm) was shown using the Agilent 1200 LC-MS with a sequential 7-minute linear gradient of 4% to 100% acetonitrile (with TFA, column and flow rate, as above) and a subsequent 4.5-minute run at 50 °C. The purity of the commercial starting materials was confirmed to be >95% (254 nm) using an ZORBAX Eclipse XDB C18 column (5 mm, 4.6 x 250 mm, Agilent Technologies) with a linear gradient of 5% to 95% acetonitrile in water (containing 10 mM triethylammonium acetate) for 20 minute at a flow rate of 1.0 mL/min. ^1H and ^{13}C NMR spectra were measured using either a Varian 400 (100) MHz or a Bruker 400 MHz instrument. Chemical shifts (δ) in ppm were relative to the small proton signals from solvent. For ^{19}F NMR (BrukerTopspin/ MestReNova 10.0.2 or 14.1.0), chemical shifts were solvent-calibrated internally. High-resolution mass spectrometry (HRMS) was performed using either an Agilent 6210 Time-

of-Flight LCMS system or a Waters Micromass spectrometer equipped with a standard interface (electrospray ionization [ESI] and modular LockSpray™).

General Procedure A.

A solution of methyl ester **23**–**26** (1.0 mmol) in THF (5 mL) was treated with triethylamine (3.0 mmol) and cooled to 0 °C in an ice bath, followed by the addition of thiophosgene (1.1 mmol). The reaction mixture was slowly warmed to room temperature and stirred from 1 h to overnight. Water was added to quench the reaction, and the mixture was extracted with ether. The organic layer was dried with $MgSO_4$ and filtered, and the filtrate was concentrated. The reaction mixture containing isothiocyanate **27**–**30** was used for the next reaction without further purification.

General Procedure B

(Cyclopropyl)methylamine or *n*-propylamine (1.1 mmol) was added to crude isothiocyanate **27**–**30** (1.0 mmol) in THF (1.5 mL), and the mixture was refluxed for 18 h. After cooling and diluting with water, the mixture was extracted with ethyl acetate, the organic layer was dried with $MgSO_4$ and filtered, and the filtrate was concentrated. The residue (compound **31**–**34**) was purified by column chromatography.

General Procedure C

Compound **31**–**34** (1.0 mmol) was dissolved in ethanol (3.0 mL), and anhydrous hydrazine (7.0 mmol) was added. The reaction mixture was heated to 80 °C for 18 h. The reaction mixture was cooled to room temperature, and the volume was reduced under a stream of nitrogen. Ethanol (3.0 mL), potassium hydroxide (3.0 mmol), and carbon disulfide (3.0 mmol) were added, and the reaction mixture was heated to 80 °C for 18 h. The reaction mixture was cooled and treated dropwise with HCl (1 N) to pH 1 with vigorous stirring, and the mixture was extracted with ethyl acetate. The organic layer was dried with $MgSO_4$ and filtered. The filtrate was concentrated. The product (compounds **3**, **5**, **11**, **13**, **15**, and **19**) was isolated from the reaction mixture by column chromatography.

General Procedure D

A mixture of the active drug (compounds **3**, **5**, **7**, **9**, **11**, **13**, **15**, **17**, and **19**) (1.0 mmol), water (3.2 mL), and sodium hydroxide (1.1 mmol) was stirred until a solution formed. The corresponding 1-methyl-4-nitroimidazole (1.0 mmol) was added to the reaction mixture, and the mixture was stirred for 3 h at room temperature. After reaction completion, the suspension was neutralized with acetic acid, yielding a solid precipitate. The precipitate was isolated by filtration, and the solid was recrystallized from ethanol. The product was purified by column chromatography.

4-(Cyclopropylmethyl)-7-methyl-1-thioxo-2,4-dihydrothieno[2,3-e][1,2,4]triazolo[4,3-a]pyrimidin-5(1*H*)-one, **5**

Methyl 3-isothiocyanato-5-methylthiophene-2-carboxylate **5** was synthesized according to General Procedure A using methyl 3-amino-5-methylthiophene-2-carboxylate, **24**, as the starting material. Compound **28** was converted to 3-(cyclopropylmethyl)-6-methyl-2-thioxo-2,3-dihydrothieno[3,2-d]pyrimidin-4(1H)-one, **32**, according to General Procedure B. Compound **32** was converted to 4-(cyclopropylmethyl)-7-methyl-1-thioxo-2,4-

dihydrothieno[2,3-e][1,2,4]triazolo[4,3-a]pyrimidin-5(1H)-one, **5**, according to General Procedure C (42%).
^{1}H NMR (400 MHz, DMSO-*d*$_6$): δ 14.09 (s, 1H), 8.68 (s, 1H), 3.90 (t, J = 7.1 Hz, 2H), 2.67 (s, 3H), 1.27 (m, 1H), 0.41–0.49 (m, 4H); HRMS (m/z): [M]+ calcd. For $C_{12}H_{13}N_4OS_2$ 293.0531; found, 293.0527.

4-(Cyclopropylmethyl)-7-methyl-1-((1-methyl-4-nitro-1*H*-imidazol-5-yl)thio)thieno[2,3-e][1,2,4]triazolo[4,3-a]pyrimidin-5(4*H*)-one, **6**
4-(Cyclopropylmethyl)-7-methyl-1-((1-methyl-4-nitro-1*H*-imidazol-5-yl)thio)thieno[2,3-e][1,2,4]triazolo[4,3-a]pyrimidin-5(4*H*)-one, **6**, was synthesized according to General Procedure D using 4-(cyclopropylmethyl)-7-methyl-1-thioxo-2,4-dihydrothieno[2,3-e][1,2,4]triazolo[4,3-a]pyrimidin-5(1*H*)-one, **5**, as the starting material (74%).
^{1}H NMR (400 MHz, DMSO-*d*$_6$): δ 8.13 (s, 1H),7.97 (s, 1H), 4.05 (d, J = 7.2 Hz, 2H), 3.85 (s, 3H), 2.70 (s, 3H), 1.32 (m, 1H), 0.46 (m, 4H); HRMS (m/z): [M]+ calcd. For $C_{16}H_{16}N_7O_3S_2$ 418.0756; found, 418.0757.

1-((1-Methyl-4-nitro-1*H*-imidazol-5-yl)thio)-4-propylbenzofuro[2,3-e][1,2,4]triazolo[4,3-a]pyrimidin-5(4*H*)-one, **8**
1-((1-Methyl-4-nitro-1*H*-imidazol-5-yl)thio)-4-propylbenzofuro[2,3-e][1,2,4]triazolo[4,3-a]pyrimidin-5(4*H*)-one, **8**, was synthesized according to General Procedure D using 4-propyl-1-thioxo-1,2-dihydrobenzofuro[2,3-e][1,2,4]triazolo[4,3-a]pyrimidin-5(4*H*)-one (**7**, compound **40** in (1)) as the starting material (27%).
^{1}H NMR (400 MHz, DMSO-*d*$_6$): δ 9.15 (d, J = 8.3 Hz, 1H), 8.15 (s, 1H), 8.00 (d, J = 8.5 Hz, 1H), 7.78 (t, J = 7.4 Hz, 1H), 7.60 (t, J = 7.3 Hz, 1H), 4.21 (t, J = 6.4 Hz, 2H), 3.88 (s, 3H), 1.79 (m, 1H), 0.93 (t, J = 7.4 Hz, 3H); HRMS (m/z): [M]+ calcd. For $C_{18}H_{16}N_7O_4S$ 426.0984; found, 426.0978.

10-Fluoro-1-((1-methyl-4-nitro-1*H*-imidazol-5-yl)thio)-4-propylbenzofuro[2,3-e][1,2,4]triazolo[4,3-a]pyrimidin-5(4*H*)-one, **10**
10-fluoro-1-((1-methyl-4-nitro-1*H*-imidazol-5-yl)thio)-4-propylbenzofuro[2,3-e][1,2,4]triazolo[4,3-a]pyrimidin-5(4*H*)-one, **10**, was synthesized according to General Procedure D using 10-fluoro-4-propyl-1-thioxo-2,4-dihydrobenzofuro[2,3-e][1,2,4]triazolo[4,3-a]pyrimidin-5(1*H*)-one (**9**, compound **41** in (1)) as the starting material (50%).
^{1}H NMR (400 MHz, DMSO-*d*$_6$): 8.11 (s, 1H), 7.91 (d, J = 8.4 Hz, 1H), 7.79–7.84 (m, 1H), 7.49–7.54 (m, 1H), 4.19 (t, J = 7.0 Hz, 2H), 3.84 (s, 3H), 1.75 (m, 1H), 0.91 (t, J = 7.4 Hz, 3H); ^{19}F NMR (376 MHz, DMSO-*d*$_6$): δ -101.39 HRMS (m/z): [M]+ calcd. For $C_{18}H_{15}FN_7O_4S$ 444.0890; found, 444.0897.

1-((1-Methyl-4-nitro-1*H*-imidazol-5-yl)thio)-4-propylthieno[2,3-e][1,2,4]triazolo[4,3-a]pyrimidin-5(4*H*)-one, **12**
1-((1-Methyl-4-nitro-1H-imidazol-5-yl)thio)-4-propylthieno[2,3-e][1,2,4]triazolo[4,3-a]pyrimidin-5(4*H*)-one, **12**, was synthesized according to General Procedure D using 4-propyl-1-thioxo-2,4-dihydrothieno[2,3-e][1,2,4]triazolo[4,3-a]pyrimidin-5(1*H*)-one (**11**, compound **42** in (1)) as the starting material (40%). ^{1}H NMR (400 MHz, DMSO-*d*$_6$): δ 8.41 (d, J = 5.5 Hz, 1H), 8.19 (d, J = 5.2 Hz, 1H), 8.14 (s, 1H), 4.14 (t, J = 7.4 Hz, 2H),

3.86 (s, 3H), 1.75 (m, 1H), 0.91 (t, J = 7.4 Hz, 3H); HRMS (m/z): [M]+ calcd. For $C_{14}H_{14}N_7O_3S_2$ 392.0600; found, 392.0601.

7-Chloro-1-((1-methyl-4-nitro-1*H*-imidazol-5-yl)thio)-4-propylthieno[2,3-e][1,2,4]triazolo[4,3-a]pyrimidin-5(4*H*)-one, **14**

7-Chloro-1-((1-methyl-4-nitro-1*H*-imidazol-5-yl)thio)-4-propylthieno[2,3-e][1,2,4]triazolo[4,3-a]pyrimidin-5(4*H*)-one, **14**, was synthesized according to General Procedure D using 7-chloro-4-propyl-1-thioxo-1,2-dihydrothieno[2,3-e][1,2,4]triazolo[4,3-a]pyrimidin-5(4*H*)-one (**13**, compound **45** in (1)) as the starting material (55%). ^{1}H NMR (400 MHz, DMSO-d_6): δ 8.29 (s, 1H), 8.14 (s, 1H), 4.12 (t, J = 7.2 Hz, 2H), 3.89 (s, 3H), 1.73 (m, 2H), 0.90 (t, J = 7.4 Hz, 3H); HRMS (m/z): [M]+ calcd. For $C_{14}H_{13}ClN_7O_3S_2$ 426.0210; found, 426.0212.

7-(tert-Butyl)-1-((1-methyl-4-nitro-1*H*-imidazol-5-yl)thio)-4-propylthieno[2,3-e][1,2,4]triazolo[4,3-a]pyrimidin-5(4*H*)-one, **16**

7-(tert-Butyl)-1-((1-methyl-4-nitro-1*H*-imidazol-5-yl)thio)-4-propylthieno[2,3-e][1,2,4]triazolo[4,3-a]pyrimidin-5(4*H*)-one, **16**, was synthesized according to General Procedure D using 7-(tert-butyl)-4-propyl-1-thioxo-2,4-dihydrothieno[2,3-e][1,2,4]triazolo[4,3-a]pyrimidin-5(1*H*)-one (**15**, compound **46** in (1)) as the starting material (53%).

^{1}H NMR (400 MHz, DMSO-d_6): δ 8.10 (s, 1H), 7.99 (s, 1H), 4.13 (t, J = 7.2 Hz, 2H), 3.84 (s, 3H), 1.73 (m, 1H), 0.90 (t, J = 7.4 Hz, 3H); HRMS (m/z): [M]+ calcd. For $C_{18}H_{22}N_7O_3S_2$ 448.1226; found, 448.1221.

1-((1-Methyl-4-nitro-1H-imidazol-5-yl)thio)-4-propylbenzo[4,5]thieno[2,3-e][1,2,4]triazolo[4,3-a]pyrimidin-5(4*H*)-one, **18**

1-((1-Methyl-4-nitro-1*H*-imidazol-5-yl)thio)-4-propylbenzo[4,5]thieno[2,3-e][1,2,4]triazolo[4,3-a]pyrimidin-5(4*H*)-one, **18**, was synthesized according to General Procedure D using 4-propyl-1-thioxo-2,4-dihydrobenzo[4,5]thieno[2,3-e][1,2,4]triazolo[4,3-a]pyrimidin-5(1*H*)-one (**17**, compound **48** in (1)) as the starting material (52%).

^{1}H NMR (400 MHz, DMSO-d_6): δ 9.42 (d, J = 8.2 Hz, 1H), 8.31 (d, J = 7.9 Hz, 1H), 8.12(s, 1H), 7.66-7.75 (m, 2H), 4.20 (t, J = 7.6 Hz, 2H), 3.80 (s, 3H), 1.79 (m, 1H), 0.92 (t, J = 7.5 Hz, 3H); HRMS (m/z): [M]+ calcd. For $C_{18}H_{16}N_7O_3S_2$ 442.0756; found, 442.0762.

7-Chloro-4-(cyclopropylmethyl)-1-thioxo-2,4-dihydrothieno[2,3-*e*][1,2,4]triazolo[4,3-*a*]pyrimidin-5(1*H*)-one, **19 (Allopole-A)**

Anhydrous hydrazine (81 μL, 2.57 mmol) was added to a solution of **33** (100 mg, 0.367 mmol) in anhydrous ethanol (6 mL) at room temperature. The reaction was sealed in a pressure tube, heated to 80 °C, and stirred overnight. The reaction was then cooled and the mixture dried with vigorous stirring under a stream of nitrogen. The crude residue was then redissolved in anhydrous ethanol (6 mL), and potassium hydroxide (61 mg, 1.10 mmol) and of carbon disulfide (222 μL, 3.67 mmol) were added. The pressure tube was once again sealed, and the reaction stirred overnight at 80 ˚C. The reaction was then cooled to room temperature and the pH was adjusted to 1 with hydrochloric acid (10 N). The mixture was then extracted three times with ethyl acetate. The organic layers were combined,

washed with water and brine, and dried over anhydrous sodium sulfate. The organic extract was decanted and concentrated in vacuo, and the residue was purified by silica gel column chromatography (hexane:ethyl acetate = 1:0 to 20:3) to produce compound **19** (6 mg, 6%). ^{1}H NMR (400 MHz, DMSO) δ 14.20 (s, 1H), 8.92 (s, 1H), 3.90 (d, *J* = 7.1 Hz, 2H), 1.29 (q, *J* = 7.6 Hz, 1H), 0.54 – 0.35 (m, 4H); HRMS (m/z): [M]+ calcd. For $C_{11}H_9ClN_4OS_2$ 312.9985; found, 312.9982.

7-Chloro-4-(cyclopropylmethyl)-1-((1-methyl-4-nitro-1*H*-imidazol-5-yl)thio)thieno[2,3-e][1,2,4]triazolo[4,3-a]pyrimidin-5(4*H*)-one, **20 (Allopole)**

7-Chloro-4-(cyclopropylmethyl)-1-((1-methyl-4-nitro-1*H*-imidazol-5-yl)thio)thieno[2,3-e][1,2,4]triazolo[4,3-a]pyrimidin-5(1*H*)-one, **20**, was synthesized according to General Procedure D using 7-chloro-4-(cyclopropylmethyl)-1-thioxo-2,4-dihydrothieno[2,3-e][1,2,4]triazolo[4,3-a]pyrimidin-5(1*H*)-one, **19**, as the starting material (78%).

^{1}H NMR (400 MHz, DMSO-*d*$_6$): δ 8.29 (s, 1H), 8.13 (s, 1H), 4.05 (d, J = 7.1 Hz, 2H), 3.90 (s, 3H), 1.46 (s, 9H), 1.30 (m, 1H), 0.47 (m, 4H); HRMS m/z (M+H) for $C_{15}H_{12}ClN_7O_3S_2$ calculated 438.0210, found 438.0217.

N-(3-(7-Fluoro-5-oxo-1-thioxo-1,2-dihydro-[1,2,4]triazolo[4,3-a]quinazolin-4(5*H*)-yl)propyl)-1-(5-(2-oxohexahydro-1H-thieno[3,4-d]imidazol-4-yl)pentanamido)-3,6,9,12-tetraoxapentadecan-15-amide **22**

Biotin *N*-hydroxysuccinimide ester (4.0 mg, 0.012 mmol) and TEA (0.0017 mL) were added to a solution of 1-amino-N-(3-(7-fluoro-5-oxo-1-thioxo-1,2-dihydro-[1,2,4]triazolo[4,3-a]quinazolin-4(5H)-yl)propyl)-3,6,9,12-tetraoxapentadecan-15-amide (54 mg, 0.01 mmol, compound **94** in (2)) in DMF (0.5 mL), and the reaction was stirred at room temperature overnight. The reaction was then concentrated, and the product was purified as a light-yellow solid using column chromatography (DCM/MeOH 90:10).

^{1}H NMR (400 MHz, methanol-*d*$_4$) δ 10.50 (dd, J = 9.3, 4.6 Hz, 1H), 7.97 (dd, J = 8.5, 3.1 Hz, 1H), 7.62 (ddd, J = 9.3, 7.7, 3.1 Hz, 1H), 4.48 (dd, J = 8.0, 4.8 Hz, 1H), 4.38 – 4.14 (m, 2H), 3.73 (t, J = 6.0 Hz, 1H), 3.61 (dd, J = 10.5, 3.8 Hz, 8H), 3.52 (t, J = 5.5 Hz, 1H), 3.27 – 3.10 (m, 2H), 2.92 (ddd, J = 12.6, 7.2, 5.0 Hz, 1H), 2.78 – 2.57 (m, 1H), 2.46 (t, J = 6.0 Hz, 1H), 2.21 (t, J = 7.3 Hz, 2H), 2.02 (p, J = 6.9 Hz, 1H), 1.84 – 1.50 (m, 2H), 1.52 – 1.22 (m, 3H), 1.03 (t, J = 7.4 Hz, 2H); HRMS (m/z): [M]+ calcd. For $C_{33}H_{47}FN_8O_8S_2$ 766.9054; found, 767.3028.

Methyl 3-amino-5-chlorothiophene-2-carboxylate, **25**

To a solution of **36** (300 mg, 1.1 mmol) in dioxane (3 mL) 20% H_2SO_4 (2.2 mmol) was added, and the resulting reaction mixture was heated to 100 ^{o}C for 6 h. The solvent was removed under reduced pressure. After addition of about 3 mL of water, the mixture was stirred for further 15 min at room temperature, extracted with ethyl acetate, dried and concentrated under reduced pressure. The residue was purified by column chromatography (ethyl acetate/hexane 30:70) to afford the title compound **25** as a light-yellow solid, 91% yield. ^{1}H NMR (400 MHz, $CDCl_3$): δ 6.43 (s, 1H), 5.54 (b, 1H), 3.88 (s, 3H).; HRMS m/z (M+H) for $C_6H_6ClNO_2S$ calculated 191.9886, found 191.9886.

Methyl 5-chloro-3-isothiocyanatothiophene-2-carboxylate, **29**

Triethylamine (2.5 mL, 17.6 mmol) and thiophosgene (410 µL, 5.27 mmol) were added to a solution of methyl 3-amino-5-chlorothiophene-2-carboxylate hydrochloride (**25**, 1.00 g, 4.38 mmol) in dry THF (50 mL) at 0 ˚C under a nitrogen atmosphere. The resulting mixture was allowed to warm to room temperature and stirred for 3 h. The reaction was diluted with water, and the THF was removed in vacuo. The resulting mixture was extracted three times with ethyl acetate and the organic layers were combined, washed with water and brine, and dried over anhydrous sodium sulfate. The organic extract was decanted and concentrated in vacuo. The crude product (**29**) was used without further purification.

6-Chloro-3-(cyclopropylmethyl)-2-thioxo-2,3-dihydrothieno[3,2-*d*]pyrimidin-4(1*H*)-one, **33**

Cyclopropylmethylamine (420 µL, 4.85 mmol) was added to a solution of **29** (1.00 g, 4.41 mmol) in dry THF (45 mL) at room temperature. The reaction was then refluxed and stirred overnight. The mixture was subsequently cooled to room temperature and concentrated in vacuo. The residue was then redissolved in anhydrous ethanol (40 mL), and potassium hydroxide (445 mg, 7.93 mmol) was added. The mixture was refluxed and stirred for 3 h. After completion of the reaction, the mixture was diluted with water and the pH was adjusted to 1 with hydrochloric acid (10 N). The resulting mixture was extracted three times with ethyl acetate. The organic layers were combined, washed with water and brine, and dried over anhydrous sodium sulfate. The organic extract was decanted and concentrated in vacuo, and the residue was purified by silica gel column chromatography (hexane:ethyl acetate = 1:0 to 3:1) to produce compound **33** (964 mg, 80%).
^{1}H NMR (400 MHz, DMSO) δ 14.19 (s, 1H), 8.90 (s, 1H), 3.90 (d, *J* = 7.1 Hz, 2H), 1.34 – 1.22 (m, 1H), 0.58 – 0.29 (m, 4H); MS (ESI, m/z) 273.0 [M+H]+; HRMS (m/z): [M]+ calcd. For $C_{10}H_{10}ClN_2OS_2$ 272.9923; found, 272.9924.

Methyl 3-acetamido-5-chlorothiophene-2-carboxylate, **36**

To a solution of methyl 3-acetamidothiophene-2-carboxylate (**35**, A2B Chem, San Diego, CA, 50.0 mg, 0.25 mmol) in chloroform (0.3 mL), sulfuryl chloride (24 µL, 0.30 mmol) was added dropwise, and the solution heated to reflux for 2 h. The solvent was evaporated, and the residue was purified by column chromatography (ethyl acetate/hexane 30:70) to afford the title compound **36** (15 mg) as a white solid, 23% yield. ^{1}H NMR (400 MHz, $CDCl_3$): δ 10.11 (s, 1H),8.03 (s, 1H), 3.86 (s, 3H), 2.21 (s, 3H).

Methyl 3-acetamido-4,5-dichlorothiophene-2-carboxylate, **37**

To a solution of methyl 3-acetamidothiophene-2-carboxylate (**35**) (50.0 mg, 0.25 mmol) in chloroform (0.3 mL), sulfuryl chloride (81 µL, 1 mmol) was added dropwise, and the solution heated to reflux for 4 h. The solvent was evaporated, and the residue was purified by column chromatography (ethyl acetate/hexane 20:80) to afford the title compound 37 as a white solid, 61% yield. ^{1}H NMR (400 MHz, $CDCl_3$): δ 8.18 (s, 1H), 3.89 (s, 3H), 2.24 (s, 3H).; HRMS m/z (M+H) for $C_8H_7Cl_2NO_3S$ calculated 267.9602, found 267.9597.

Off-target screening

Off-target was determined by the the Psychoactive Drug Screening Program (PDSP) at the University of North Carolina. We thank Dr. Bryan L. Roth (University of North

Carolina at Chapel Hill) and the National Institute of Mental Health's Psychoactive Drug Screening Program (Contract # HHSN-271-2008-00025-C) for screening data. Reference: Besnard, J.; Ruda, G. F.; Setola, V.; Abecassis, K.; Rodriguiz, R. M.; Huang, X. P.; Norval, S.; Sassano, M. F.; Shin, A. I.; Webster, L. A.; Simeons, F. R.; Stojanovski, L.; Prat, A.; Seidah, N. G.; Constam, D. B.; Bickerton, G. R.; Read, K. D.; Wetsel, W. C.; Gilbert, I. H.; Roth, B. L.; Hopkins, A. L. Automated design of ligands to polypharmacological profiles. *Nature* **2012**, *492*, 215–220. Procedures: https://pdsp.unc.edu/pdspweb/content/UNC-CH%20Protocol%20Book.pdf

No significant in vitro interactions (<50% inhibition at 10 μM) for any of the compounds were found at the following sites (human, unless otherwise noted): $5HT_{1A}$, $5HT_{1B}$, $5HT_{1D}$, $5HT_{1E}$, $5HT_{2A}$, $5HT_{2B}$, $5HT_{2C}$, $5HT_3$, $5HT_{5A}$, $5HT_6$, $5HT_7$, α_{1A}, α_{1B}, α_{1D}, α_{2A}, α_{2B}, α_{2C}, β_1, β_2, β_3, BZP rat brain site, D_1, D_2, D_3, D_4, D_5, δ-opioid receptor (DOR), $GABA_A$, H_1, H_2, H_3, H_4, M_1, M_2, M_5, κ-opioid receptor (KOR), μ-opioid receptor (MOR), σ_1, σ_2, DAT, NET, SERT. Additional detailed information is provided in *SI Appendix,* Table S5.

Summary of results:

PDSP no.	Compound	Interactions (K_i, μM)
60133	**6**	None observed.
60389	**5**	BZP rat, 0.41.
60477	**Allopole-A**	None observed.
60478	**Allopole**	5-HT_{2A}, 9.2; 5-HT_{2C}, 1.3.

Fig. S1.

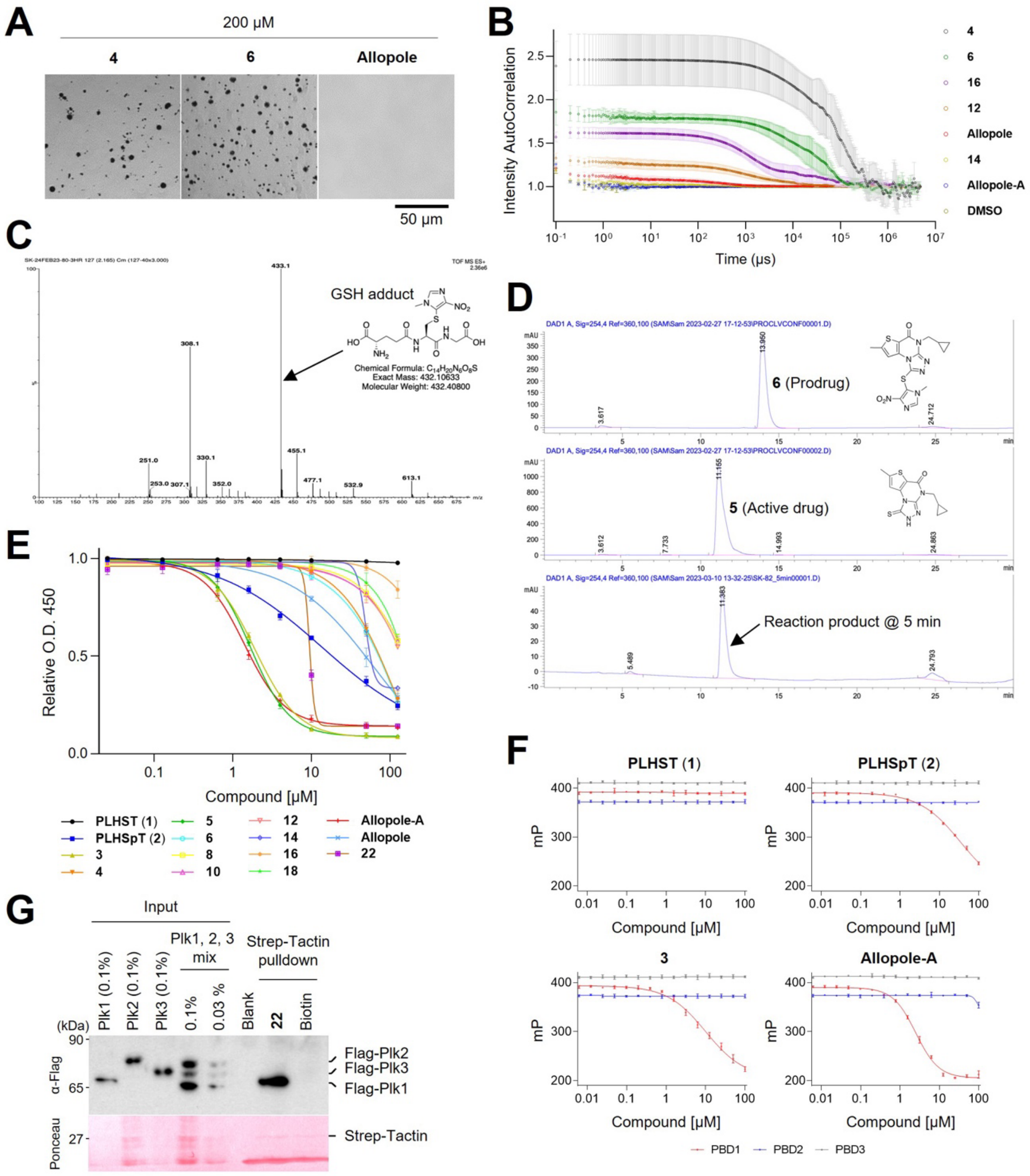

Fig. S1. Solubility and activity of Allopole and its related compounds. (*A*) Images showing insoluble particles of 200 μM of the indicated compounds dissolved in the PBS buffer. The aggregates of moderately soluble compounds (**12** and **16**) were not large enough for microscopic imaging. (*B*) Dynamic light scattering profile for the indicated compounds dissolved in phosphate-buffered saline (pH 7.4) buffer. (*C*) Mass spectrometry spectrum showing the detection of the GSH adduct with the prodrug 5-thio-1-methyl-4-nitroimidazolyl moiety. (*D*) HPLC profiles showing control elution profiles for compound **6** (prodrug) (top) and its active form **5** (unmasked active form) (middle) and the product detected after reacting **6** with GSH for 5 minutes at room temperature (bottom) (see Methods for details). © ELISA-based assays performed to determine anti-PBD1 activity as described in Methods. Quantified data obtained from three or more (number indicated in parentheses) independent

experiments are provided in *SI Appendix,* Table S1. Bars, mean of three independent experiments ± s.d. (*F*) FP-based assays carried out to determine PBD1-binding specificity as described in Methods. A pair of previously characterized phosphopeptides, PLHSpT (**2**, K_d of 450 nM) and its respective non-phospho form (**1**) (3), were included as controls. Bars, mean of three independent experiments ± s.d. IC_{50} values determined from three experiments are provided in *SI Appendix,* Table S2. (*G*) Pull-down and immunoblotting analyses showing that the biotinylated compound **22** specifically interacts with Plk1 but not Plk2 or Plk3 in the mixed total lysates prepared from HEK293 cells expressing each of the Flag-fused constructs (lanes 1–3). The same membrane stained with Ponceau S is provided.

Fig. S2.

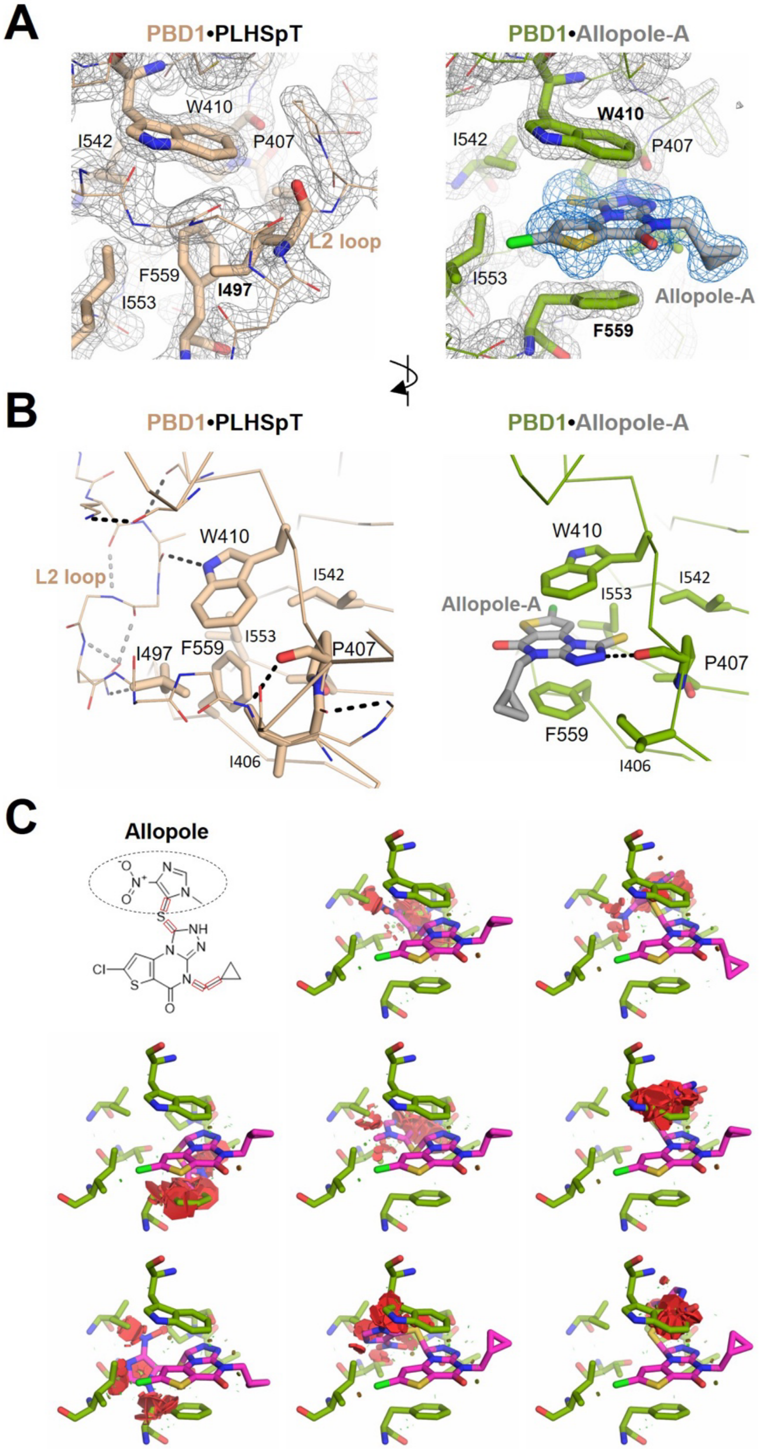
A
PBD1•PLHSpT
W410
I542
P407
L2 loop
F559
I497
I553
PBD1•Allopole-A
W410
I542
P407
I553
Allopole-A
F559
B
PBD1•PLHSpT
W410
L2 loop
I542
I553
I497
F559
P407
I406
PBD1•Allopole-A
W410
I542
Allopole-A
I553
P407
F559
I406
C
Allopole

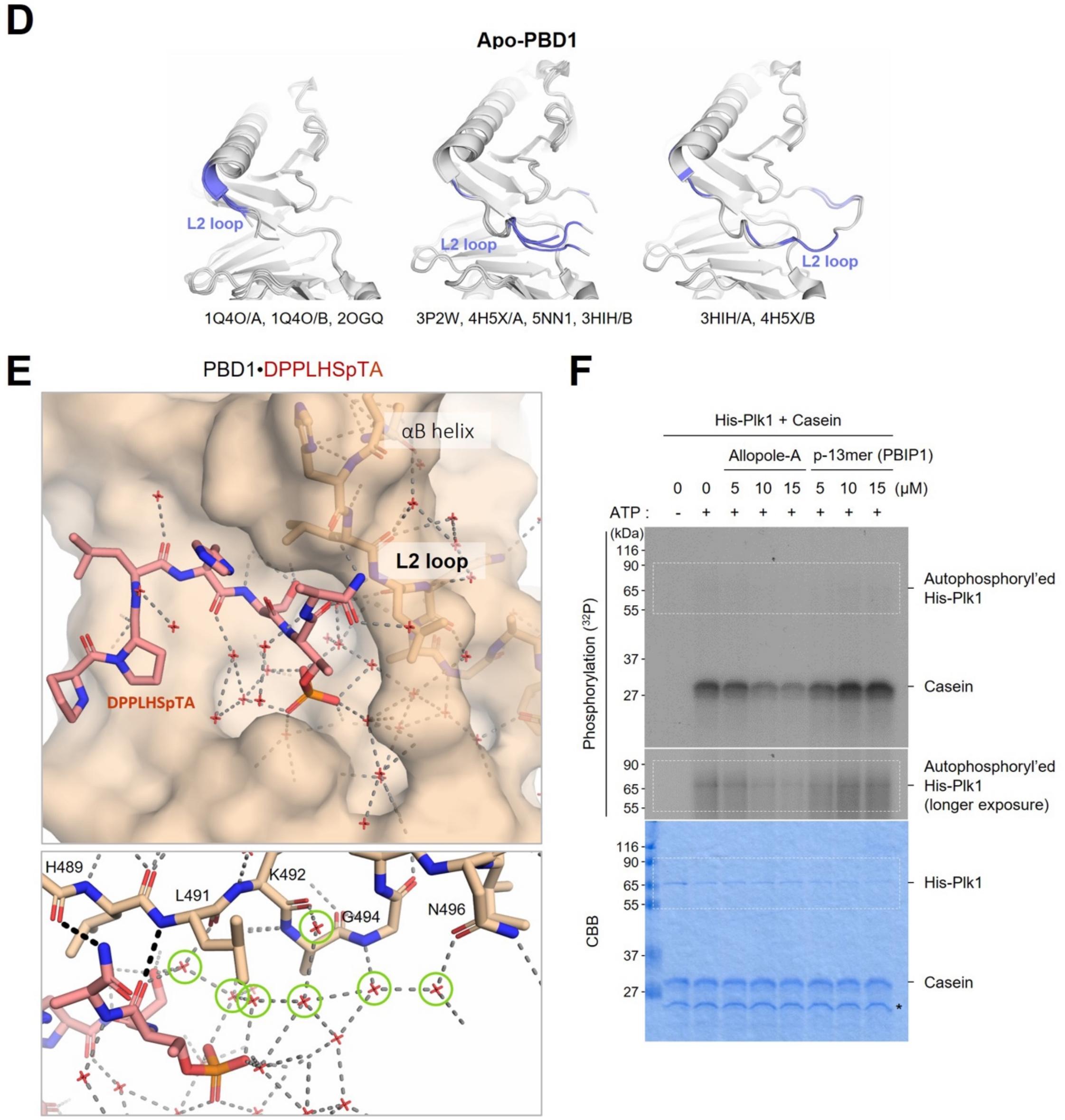

Fig. S2. Nature of Allopole-A binding versus PLHSpT binding to the PBD1. (*A* and *B*) Structural changes in the W-F pocket before (PBD1-PLHSpT; PDB 3HIK) and after Allopole-A binding (PBD1•Allopole-A; PDB 8CRC), showing the displacement of the L2 loop upon Allopole-A binding. (*A*) The 2*Fo–Fc* electron density map of the protein is contoured to 1.5 σ (shown as gray mesh). Simulated annealing *Fo-Fc* omit map of Allopole-A is shown at 2.0 σ as blue mesh. The black dashed line in (*B*, left) indicates a H-bond between the L2 loop and W410, which is disrupted by Allopole-A (*B*, right). The Allopole-A triazole ring also establishes a H-bond with the P407 carbonyl group. (*C*) Three-dimensional conformers for Allopole were generated using the RDKit rdk_confgen.py script. Allopole (top, left) has 4 rotatable bonds (indicated by red rectangles) capable of generating 12 possible conformers of the prodrug moiety (dotted circle). Among them, the top-8-lowest-energy Allopole structures (magenta in 8 models with PBD1 in green) were used for van der Waals clash analysis. The clashes were visualized by Pymol using the show_bump.py Python module. In all cases, severe van der Waals clashes (marked by red disks) were observed. (*D*) An ensemble of apo-PBD1 structures

generated by combining L2 loop–lacking (1Q4O chain A and B and 2OGQ), L2 loop–truncated (3P2W, 4H5X chain A, 5NN1, 3HIH chain B), and L2 loop–containing (3HIH chain A and 4H5X chain B) structures. The regions colored in purple blue indicate crystal packing contacts. This indicates that the truncated and full lengths L2 loops became ordered due to the packing contacts. (*E*) A structural model showing PBD1–DPPLHSpTA interactions (PDB 3P36) mediated by a network of multiple water molecules (green circles) and two H-bonds between H489 and L491 of PBD1 and the DPPLHSpTA backbone (black dashed lines). Note that L491, K492, G494, and N496 residues collectively establish the chain of water-mediated interactions with the phospholigand, DPPLHSpTA. These water network-mediated interactions remain unchanged in the crystal structure of the PBD1–PLHSpT complex (PDB 3HIK), where a H-bond is formed similarly between L491 and the peptide. (*F*) In vitro kinase assays performed with His-Plk1 and casein as described in the Methods. An autoradiogram showing ^{32}P incorporated onto casein (top) and autophosphorylated Plk1 (dotted box) is provided along with the Coomassie Brilliant Blue (CBB)-stained gel. Protein bands excised from the gel were quantified using a scintillation counter and the results are provided in Fig. 2*E*. Asterisk, contaminating protein from casein.

Fig. S3.

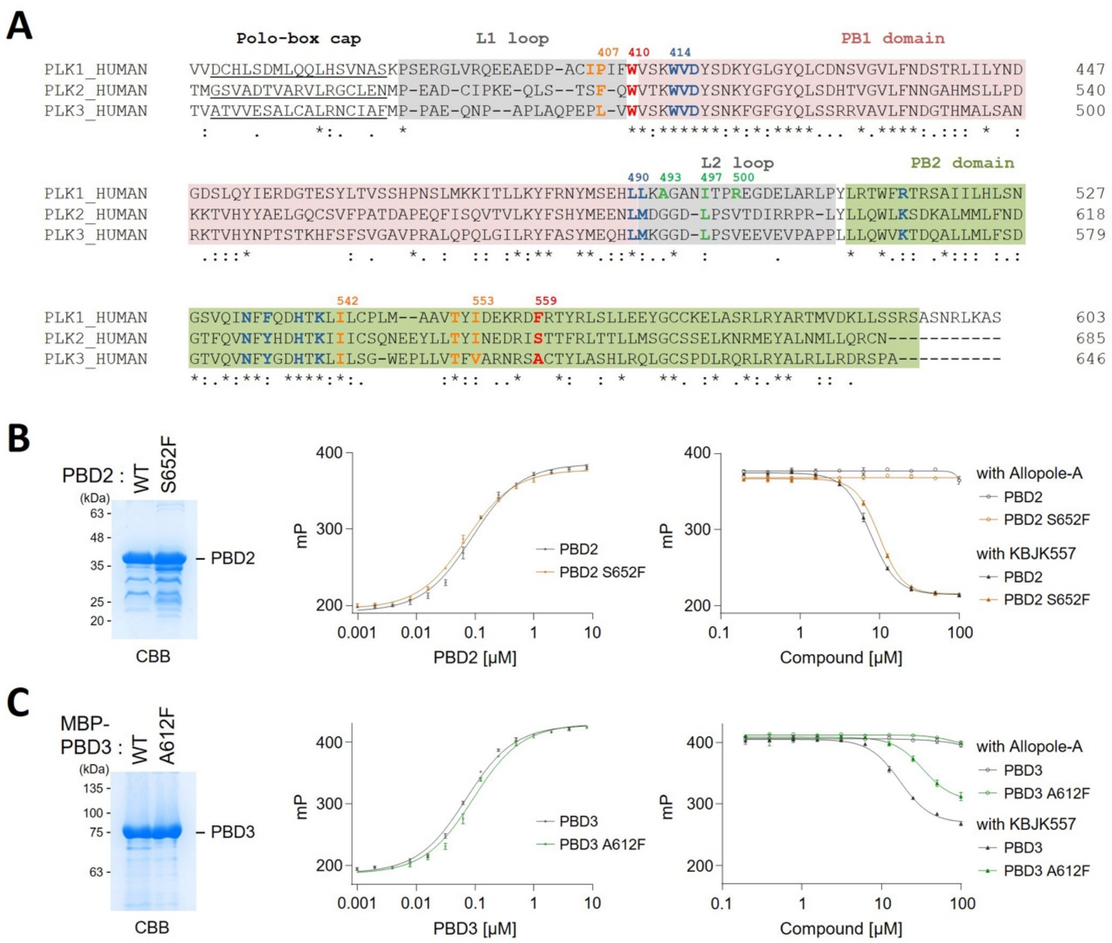

D

Protein	K_d ± SD [nM]	EC_{85} [nM]
PBD2	80.15 ± 6.31	390
PBD2 S652F	62.71 ± 3.88	370
MBP-PBD3	62.4 ± 1.38	350
MBP-PBD3 A612F	85.40 ± 8.36	400

Fig. S3. Sequence alignment showing unique PBD2 S652 and PBD3 A612 residues and FP-based Allopole-A inhibition assays carried out with PBD2 S652F and PBD A612F mutants. (*A*) Human PBD1 (Uniprot ID: P53350), PBD2 (Uniprot ID: Q9NYY3), and PBD3 (15niport ID:Q9H4B4) sequences were aligned using Clustal Omega. The polo-box cap and L1 loop regions were adjusted based on the structural superposition of PBD1 (PDB ID: 3HIK), PBD2 (PDB_ID: 4RS6), and the AlphaFold model of PBD3 (https://alphafold.ebi.ac.uk). PB1 and PB2 domains are labeled with pink and green boxes, respectively. Residues in the WF pocket are shown in bold red and bold orange letters. Residues important for phospholigand binding are labeled in bold blue letters. L2 loop anchor points binding to the WF pocket (A493, I497, and R500) are labeled in bold green letters. (*B* and *C*) Using purified PBD2 or MBP-PBD3 proteins (left), comparative FP binding (middle) and inhibition (right) assays were performed as in *SI Appendix,* Fig. S1*F*. KBJK557 (4) was included to show that the inhibition assays with PBD2 and MBP-PBD3 are working properly under our conditions. CBB, Coomassie Brilliant Blue-stained gel. **(***D***)** Summary of K_d and EC_{85} values for the indicated proteins. The K_d values for the indicated proteins were determined after extrapolating them from the binding curves as the

concentration at which 50% of FITC-conjugated ligands (FITC-GPMQTSpTPKNG for PBD2 and FITC-GPLATSpTPKNG for PBD3) (5) are protein-bound. The EC_{85} values indicate the effective concentration of 85% of the maximal concentration calculated for each protein. The FP values were obtained 30 minutes after incubation.

Fig. S4.

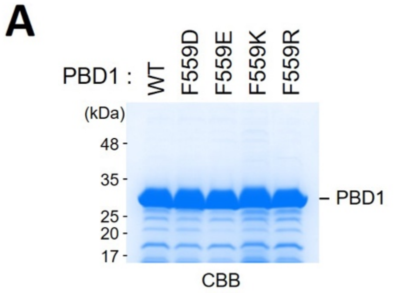

B

PBD1	K_d ± SD [nM]	EC_{85} [nM]
WT	16.80 ± 1.42	75
F559D	28.14 ± 0.84	140
F559E	124.00 ± 1.45	450
F559K	703.97 ± 104.69	N.D.
F559R	459.9 ± 22.61	N.D.

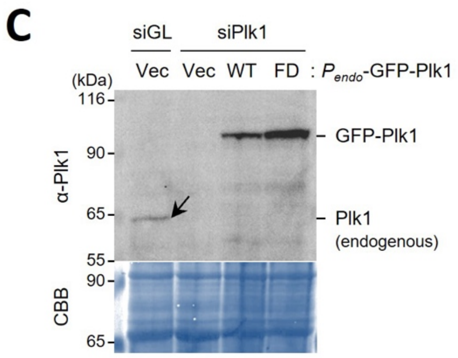

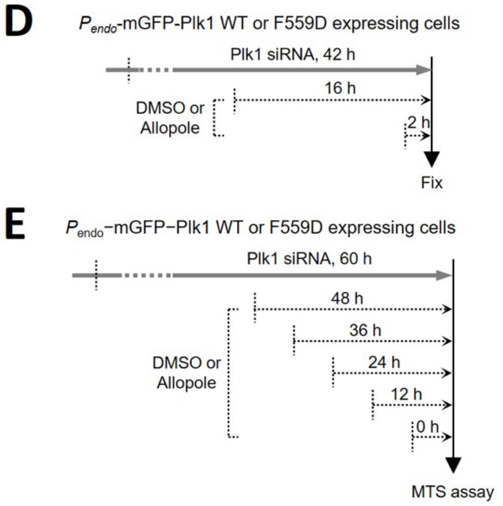

Fig. S4. Generation of F559 mutants for in vitro and cell-based assays in Fig. 4. (*A*) SDS-PAGE *showing the proteins used for the FP assays in Fig. 4 A* and *B. CBB, a Coomassie Brilliant Blue–stained gel.* (*B*) Summary of K_d and EC_{85} values for PBD1 WT and mutants. The K_d values for PBD1 WT and mutants were determined after extrapolating them from the binding curves as the concentration at which 50% of the FITC-Ahx-DPPLHSpTAI-NH_2 are PBD1-bound. The EC_{85} values indicate the effective concentration of 85% of the maximal concentration calculated for each protein. The FP values were obtained 30 minutes after incubation. For the F559K and F559R mutants, we were not able to determine the EC_{85} values and therefore used 2 μM of these proteins for the assays in Fig. 4*B*. *N.D., not determined.* (*C*) Immunoblotting analysis showing the level of mGFP-Plk1 WT and the F559D mutant expressed under the endogenous Plk1 promoter after depleting endogenous Plk1. Arrow, endogenous Plk1. (*D* and *E*) Detailed experimental schedules for Fig. 4*D* and 4*E*, respectively.

Table S1.

Table S1. Anti-PBD1 activity of **4**-derived heterocyclic inhibitors determined by ELISA-based and L363 cell–based assays.

**Compound**	**Structure**	**Anti-Plk1 (full length) ELISA (IC_{50})* Mean ± SEM (μM)[a]**	**Cell efficacy (GI_{50}) Mean ± SD (μM)[b]**
**1**, PLHST	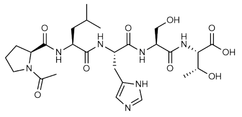	>50	N. S.
**2**, PLHSpT	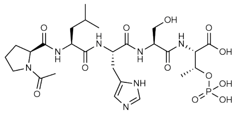	13.97 ± 1.00	N. S.
**3****	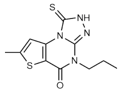	2.15 ± 0.13 (6)	N. S.
**4**** (prodrug of **3**)	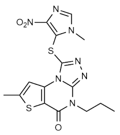	>50	4.88 ± 0.10 (low solubility)
**5**	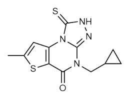	1.66 ± 0.04 (4)	N. S.
**6** (prodrug of **5**)	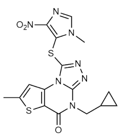	>50	3.13 ± 0.15 (low solubility)
**8** (prodrug)	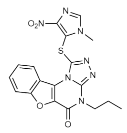	>50	8.31 ± 0.56
**10** (prodrug)	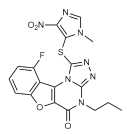	>50	7.53 ± 0.43
**12** (prodrug)	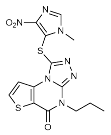	>50	8.40 ± 0.74 (moderate solubility)
**14** (prodrug)	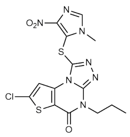	>50	6.04 ± 0.17

**16** (prodrug)	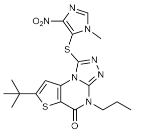	>50	4.19 ± 0.15 (moderate solubility)
**18** (prodrug)	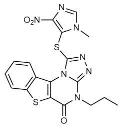	>50	7.05 ± 0.27
**19** Allopole-A	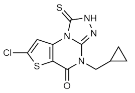	1.40 ± 0.03	N. S.
**20** (prodrug of **19**), Allopole	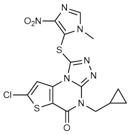	>25	4.09 ± 0.14
**21*** **	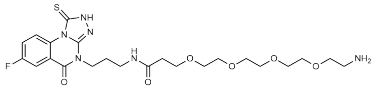	5.36 ± 0.40	N. D.
**22**	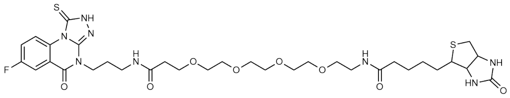	9.32 ± 0.17	N. D.

*Assays were performed with total cellular lysates from HEK293A cells expressing the full-length Plk1. **Data from Park, J.E., et al. (1). *** Data from Alverez C.N., et al. (2). N. S., not significant; N. D., not determined.

[a] IC_{50} values were determined from at least three independent experiments except as noted in parentheses.

[b] A colorimetric MTS cell viability assay was carried out using multiple myeloma–derived L363 cells. The concentration that inhibited cell growth by 50% (GI_{50}) was determined from at least three independent experiments.

Table S2.

Table S2. Conversion of Allopole to Allopole-A under various buffer conditions, room temperature, 1 h

Buffers*	Compounds detected				
		**Allopole-A**	**Allopole-A (-S)**	**Allopole (-Cl)**	**Allopole**
**#1**	pk area	9.61E+06	6.64E+06	4.85E+04	7.45E+08
	umol/L	1.3	0.9	0.0	97.9
	% of total	1.3	0.9	0.0	97.9
**#1** + 5 mM GSH	pk area	1.38E+09	5.81E+07	6.89E+06	8.76E+05
	umol/L	95.4	4.0	0.5	0.1
	% of total	95.4	4.0	0.5	0.1
**#2**	pk area	4.90E+06	1.05E+07	2.42E+04	6.28E+08
	umol/L	0.8	1.6	0.0	97.6
	% of total	0.8	1.6	0.0	97.6
**#2** + 5 mM GSH	pk area	5.81E+08	1.63E+08	2.57E+06	2.41E+03
	umol/L	77.9	21.8	0.3	0.0
	% of total	77.9	21.8	0.3	0.0
**#3**	pk area	1.56E+06	4.21E+06	0.00E+00	7.80E+07
	umol/L	1.9	5.0	0.0	93.1
	% of total	1.9	5.0	0.0	93.1
**#3** + 5 mM GSH	pk area	1.89E+08	9.21E+07	1.11E+06	9.35E+04
	umol/L	66.9	32.7	0.4	0.0
	% of total	66.9	32.7	0.4	0.0

* Buffer #1: 20 mM Tris (pH 8.0), 50 mM NaCl, 1 mM EDTA, 1% DMSO
Buffer #2: 1 x PBS (pH 7.4), 1 mM EDTA, 1% DMSO
Buffer #3: 20 mM HEPES (pH 7.0), 50 mM NaCl, 1 mM EDTA, 1% DMSO

Samples were prepared by incubating 100 μM of Allopole in the indicated buffer conditions in the absence or presence of 5 mM GSH for 1 h at room temperature. Note that in the absence of GSH, Allopole remains stable.

Table S3.

Table S3. FP-based inhibition assays against Plk1–3 PBDs.

Compound	Respective prodrug	PBD1* IC_{50} ± SD [nM]	PBD2* IC_{50} ± SD [nM]	PBD3* IC_{50} ± SD [nM]
**2**, PLHSpT	–	35.02 ± 5.8	N. S.	N. S.
**3**, NCK149	**4**, NCK173	10.17 ± 1.5	N. S.	N. S.
**5**, NCK181	**6**, NCK182	12.83 ± 2.6	N. S.	N. S.
**19**, Allopole-A	**20**, NCK190	2.53 ± 0.18	N. S.	N. S.

*Assays were performed with bacterially expressed purified Plk1–3 PBDs. N. S., not significant. IC_{50} values were determined from at least three independent experiments.

Table S4.

Table S4. Data collection and refinement statistics (molecular replacement)

	Plk1-PBD + Allopole-A
**Data collection**	
Space group	P 1 21 1
Cell dimensions	
a, *b*, *c* (Å)	39.36 51.90 51.45
α, β, γ (°)	90.00 106.64 90.00
Resolution range (Å)	49.29 - 1.65
$CC_{1/2}$	99.9 (88.3)
R_{merge}†	6.8 (88.1)
$<I/\sigma(I)>$	9.7 (2.0)
Completeness (%)	97.6
Redundancy	7.0
No. reflections	23444
**Refinement**	
$R_{work/}$ R_{free}	0.177/0.219
No. atoms	
Protein	1746
Ligand/ion	25
Solvent	180
B-factors (Å^2)	
Protein	36.42
Ligand	39.56
Solvent	47.46
Ramachandran	
Favored (%)	95.31
Allowed (%)	4.69
Outliers (%)	0
Rotamer outliers (%)	0
R.m.s deviations	
Bond lengths (Å)	0.013
Bond angles (º)	1.351
PDB ID	8CRC

†R merge = $\Sigma_{hkl}\Sigma_i |I_i(hkl)-<I(hkl)>|/\Sigma_{hkl}\Sigma_i I_i(hkl)$

Table S5.

Table S5. Off-target K_i values for selected compounds described in this study

Receptor	Allopole-A		Allopole	
	Primary screen[a] AVE(%inhib)±SD	Secondary screen[b] K_i (μM)	Primary screen[a] AVE(%inhib)±SD	Secondary screen[b] K_i (μM)
5-HT1A	−20.0±27.4	>10	−12.8±19.7	>10
5-HT1B[d]	17.7±42.2	>10	60.8±40.9	>10
5-HT1D	22.5±25.6	>10	32.8±27.1	>10
5-HT1E	31.3±7.6	>10	22.2±10.5	>10
5-HT2A[d]	37.0±10.4	>10	52.4±11.8	9.2
5-HT2B	−17.7±11.2	>10	−0.5±5.0	>10
5-HT2C[d]	−18.3±22.0	>10	59.5±3.0	1.3
5-HT3	−3.8±3.1	>10	−0.8±4.6	>10
5-HT5A	−1.4±14.4	>10	24.5±9.3	>10
5-HT6	−5.0±11.7	>10	21.9±15.6	>10
5-HT7A	−88.1±20.8	>10	14.9±9.9	>10
Alpha1A	−9.4±18.0	>10	40.6±10.5	>10
Alpha1B	−7.7±9.7	>10	−1.6±9.5	>10
Alpha1D	−9.9±23.4	>10	−15.1±19.8	>10
Alpha2A	−6.3±7.9	>10	9.4±4.6	>10
Alpha2B	−6.4±7.1	>10	8.3±16.4	>10
Alpha2C	10.6±4.2	>10	6.4±7.5	>10
Beta1[d]	−6.2±19.4	>10	67.3±49.1	>10
Beta2	−2.1±19.7	>10	0.1±10.6	>10
Beta3	17.9±8.6	>10	30.8±22.8	>10
BZP Rat brain site	2.3±21.8	>10	9.5±7.6	>10
D1	−17.2±25.0	>10	21.5±6.8	>10
D2	−27.4±13.1	>10	3.6±6.6	>10
D3	−7.0±21.7	>10	12.8±12.8	>10
D4	−24.3±4.4	>10	12.6±4.9	>10
D5	28.7±10.7	>10	20.9±9.5	>10
DAT[d]	43.9±13.0	>10	54.0±10.7	>10
DOR	−13.5±6.8	>10	−1.0±4.8	>10
GABAA	35.7±11.1	>10	33.3±4.2	>10
H1	11.5±2.8	>10	35.8±11.9	>10
H2	−27.4±17.1	>10	−16.4±21.5	>10
H3	−18.5±3.8	>10	−12.5±7.9	>10
H4	−4.5±11.4	>10	8.2±3.1	>10
KOR	−0.3±8.7	>10	3.7±4.1	>10
M1[d]	39.3±10.7	>10	50.7±26.1	>10
M2	21.1±19.7	>10	9.3±19.3	>10
M3	7.5±45.6	>10	48.3±21.9	>10
M4	−8.8±4.8	>10	−8.7±9.5	>10
M5[c,d]	130.2±3.0	>10	95.0±7.4	>10
MOR	30.8±47.5	>10	40.6±51.0	>10
NET	−39.3±20.6	>10	−56.8±10.5	>10

PBR	5.3±8.3	>10	-2.1±5.3	>10
SERT	−28.9±28.7	>10	−56.0±13.9	>10
Sigma 1	2.0±21.4	>10	41.8±11.9	>10
Sigma 2	40.6±9.5	>10	40.7±5.8	>10

[a] Primary screening performed at 10 μM, and only a percent inhibition greater than 50% would trigger a secondary screen at that receptor.

[b] Secondary screening results from full inhibition curves are expressed as K_i (μM). When the secondary screen K_i is listed as >10 μM, it means that the primary binding results were not validated in the secondary screen.

[c] Data of the secondary screen of Allopole-A indicated no significant interaction.

[d] Data of the secondary screen of Allopole indicated no significant interaction.

Table S6.

Table S6. Antibodies used in this study

Antibodies	Species	Source	Cat #	Exp. dilution
Primary antibodies				
Anti-PBIP	Rabbit	(6)	Lab supply	1:500 (IB)
Anti-Bub1	Rabbit	(7)	Lab supply	1:500 (IB)
Anti-BubR1	Mouse	BD Biosciences	BD612503	1:1000 (IB)
Anti-Cdc25C (C-20)	Rabbit	Santa Cruz Biotech	sc-327	1:1000 (IB)
Anti-Cdc25C pT48	Rabbit	Cell Signaling	#9527	1:1000 (IB)
Anti-β-catenin	Mouse	BD Biosciences	BD610154	1:1000 (IB)
Anti-TCTP	Rabbit	Cell Signaling	#8441	1:1000 (IB)
Anti-TCTP pS46	Rabbit	Cell Signaling	#5251	1:1000 (IB)
Anti-FLAG (M2)	Mouse	Millipore Sigma	F1804	1:1000 (IB)
Anti-FLAG M2 Affinity Gel	Mouse	Millipore Sigma	A2220	
Anti-FLAG (M2)-HRP conjugate	Mouse	Millipore Sigma	A8592	1:10000 (IB)
Anti-Plk1 (F-8)	Mouse	Santa Cruz Biotech	sc-17783	1:400 (IB), 1:100 (IF)
Anti-Cep192 (1-647)	Rabbit	(8)	Lab supply	1:200 (IF)
Anti-Crest	Human	Antibodies, Inc.	15-234	1:500 (IF)
Secondary antibodies (Immunofluorescence)				
Anti-mouse IgG Alexa Fluor 594-conjugated	Donkey	Invitrogen	A-21203	1:300 (IF)
Anti-rabbit IgG Alexa Fluor 647-conjugated	Donkey	Invitrogen	A-31573	1:300 (IF)
Anti-human IgG Alexa Fluor 488-conjugated	Goat	Invitrogen	A-11013	1:300 (IF)

Movie S1 (separate file).

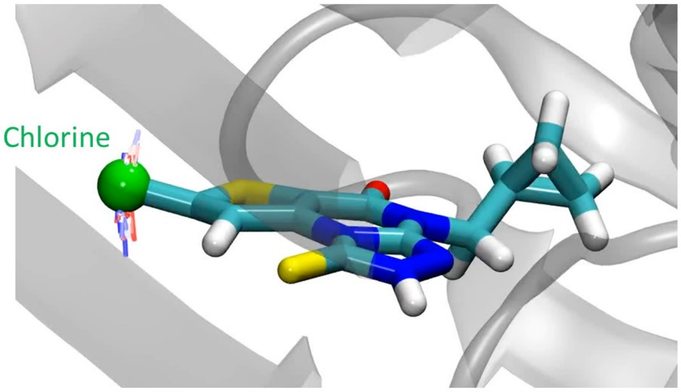

Movie S1. Molecular simulation showing the flexibility of the chloride moiety of Allopole-A cocrystallized with PBD1. To mimic the crystallized state of the PBD1•Allopole-A complex (PDB 8CRC), simulation was performed after restraining the positions of the PBD1 residues in the presence of a symmetry mate (see Methods for details). The movement of the chloride moiety (colored lines) was tracked during the entire length of time (200 ns~~ec~~).

Movie S2 (separate file).

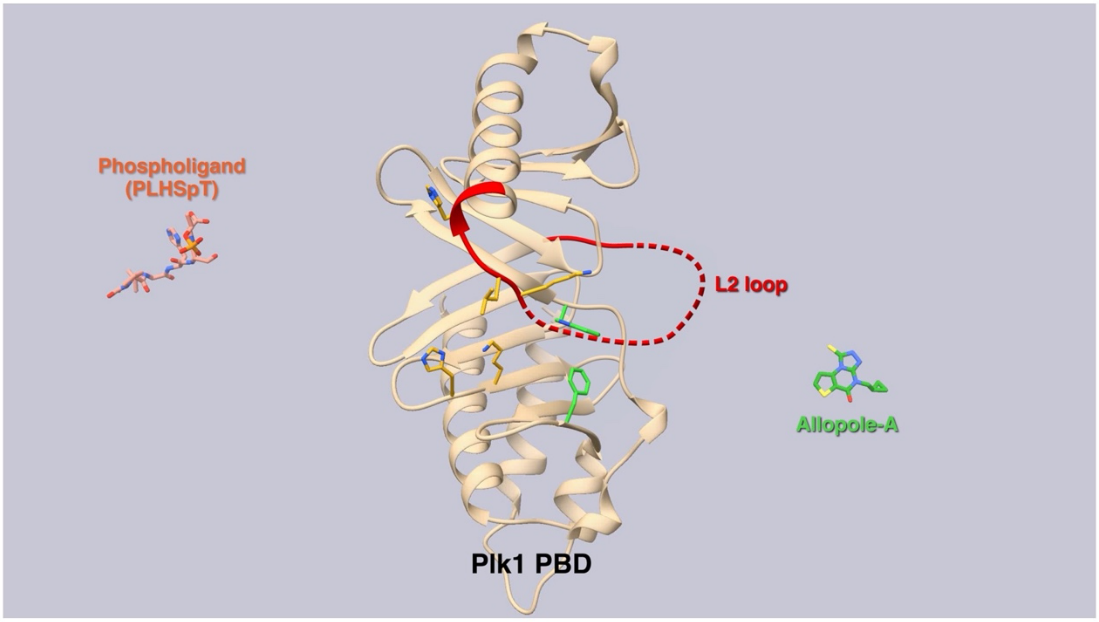

Movie S2. The mechanism underlying how Allopole-A inhibits the PBD1•phospholigand interaction. See Fig. 5 for details. Apo-PBD1, PBD1•PLHSpT, and PBD1•Allopole-A structures were modeled using PDBs 1Q4O, 3HIK, and 8CRC, respectively. See Fig. 5 for details.

SI Appendix, References

1. J.-E. Park *et al.*, Structural Optimization and Anticancer Activity of Polo-Like Kinase 1 (Plk1) Polo-Box Domain (PBD) Inhibitors and their Prodrugs. *ACS Pharmacol. Transl. Sci.* 6, 422-444 (2023).
2. C. N. Alverez *et al.*, Identification of a New Heterocyclic Scaffold for Inhibitors of the Polo-Box Domain of Polo-like Kinase 1. *J Med Chem* **63**, 14087-14117 (2020).
3. S. M. Yun *et al.*, Structural and functional analyses of minimal phosphopeptides targeting the polo-box domain of polo-like kinase 1. *Nature structural & molecular biology* **16**, 876-882. (2009).
4. P. Gunasekaran *et al.*, Development of a Polo-like Kinase-1 Polo-Box Domain Inhibitor as a Tumor Growth Suppressor in Mice Models. *J Med Chem* **63**, 14905-14920 (2020).
5. W. Reindl, M. Gräber, K. Strebhardt, T. Berg, Development of high-throughput assays based on fluorescence polarization for inhibitors of the polo-box domains of polo-like kinases 2 and 3. *Anal. Biochem.* **395**, 189-194. (2009).
6. Y. H. Kang *et al.*, Self-regulated Plk1 recruitment to kinetochores by the Plk1-PBIP1 interaction is critical for proper chromosome segregation. *Mol. Cell* **24**, 409-422 (2006).
7. Z. Tang, R. Bharadwaj, B. Li, H. Yu, Mad2-Independent inhibition of APC^{Cdc20} by the mitotic checkpoint protein BubR1. *Dev. Cell* **1**, 227-237 (2001).
8. T.-S. Kim *et al.*, Hierarchical recruitment of Plk4 and regulation of centriole biogenesis by two centrosomal scaffolds, Cep192 and Cep152. *Proc. Natl. Acad. Sci. USA* **110**, E4849-4857 (2013).

Source Data

Fig. 1*C*

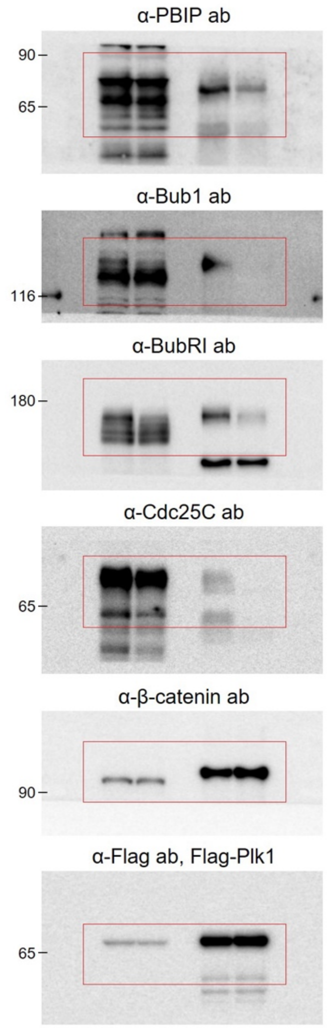

Fig. 4F

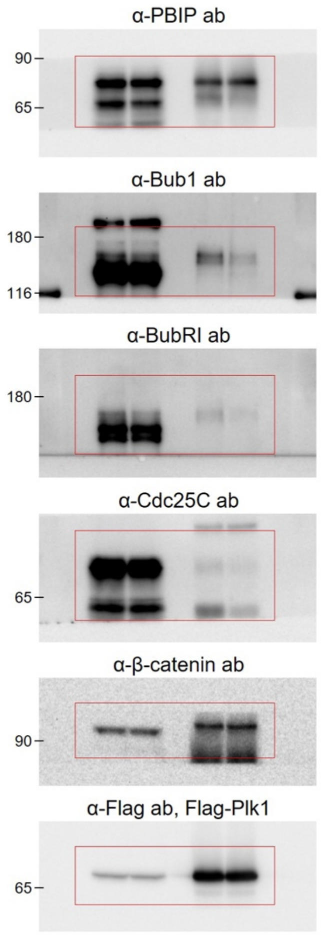

SI Appendix,* Fig. S1*G

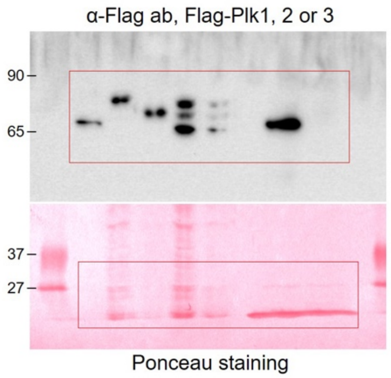

***SI Appendix,* Fig. S2F**

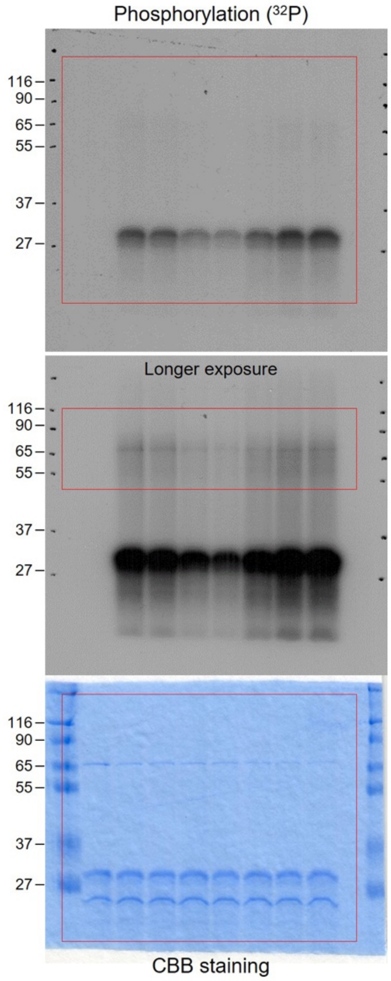

***SI Appendix,* Fig. S3B**

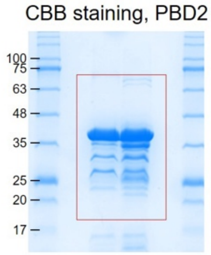

***SI Appendix,* Fig. S3C**

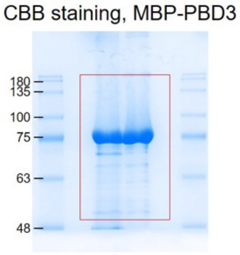

Red boxes indicate the regions cropped to generate figures

SI Appendix*, Fig. S4*A

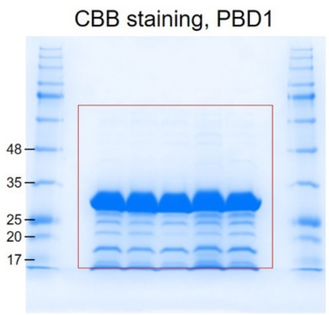

SI Appendix*, Fig. S4*C

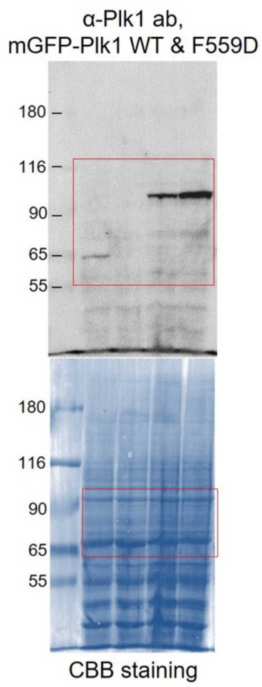

Red boxes indicate the regions cropped to generate figures

Original data (NMR and Mass spectrum)
for all the compounds described in this study

Compound 5

^{1}H NMR spectrum

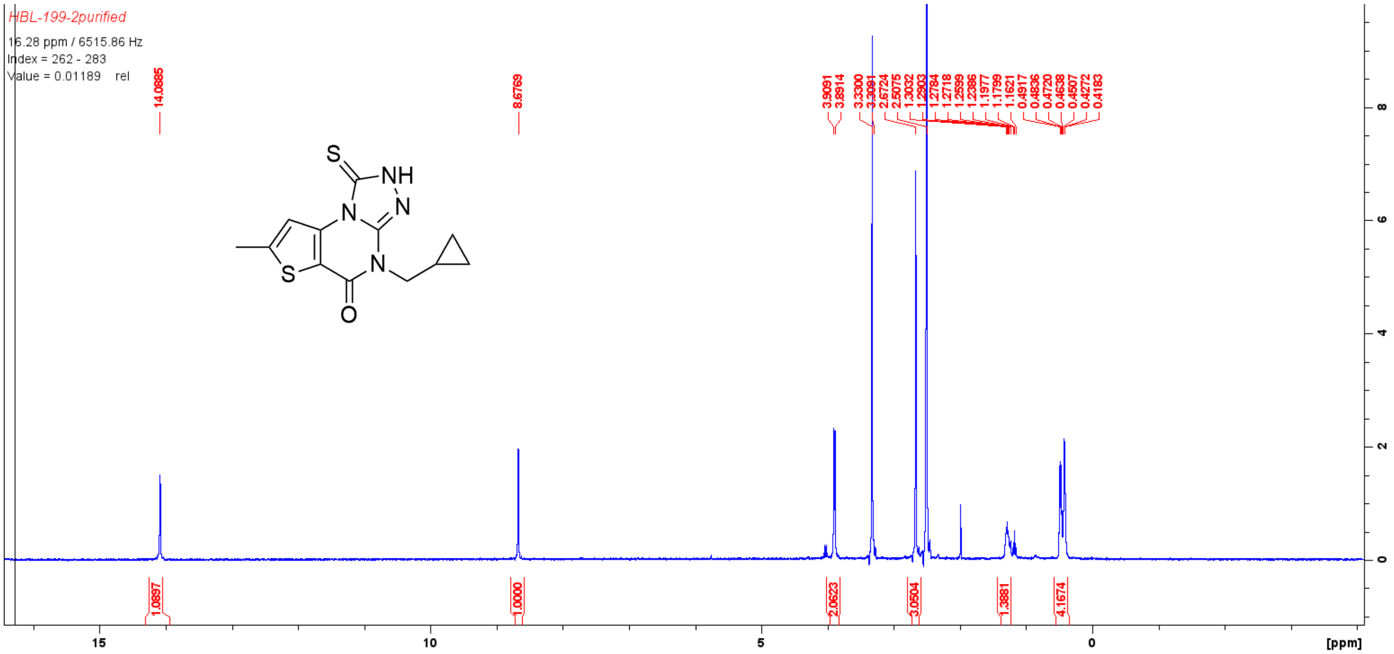

Mass spectrum

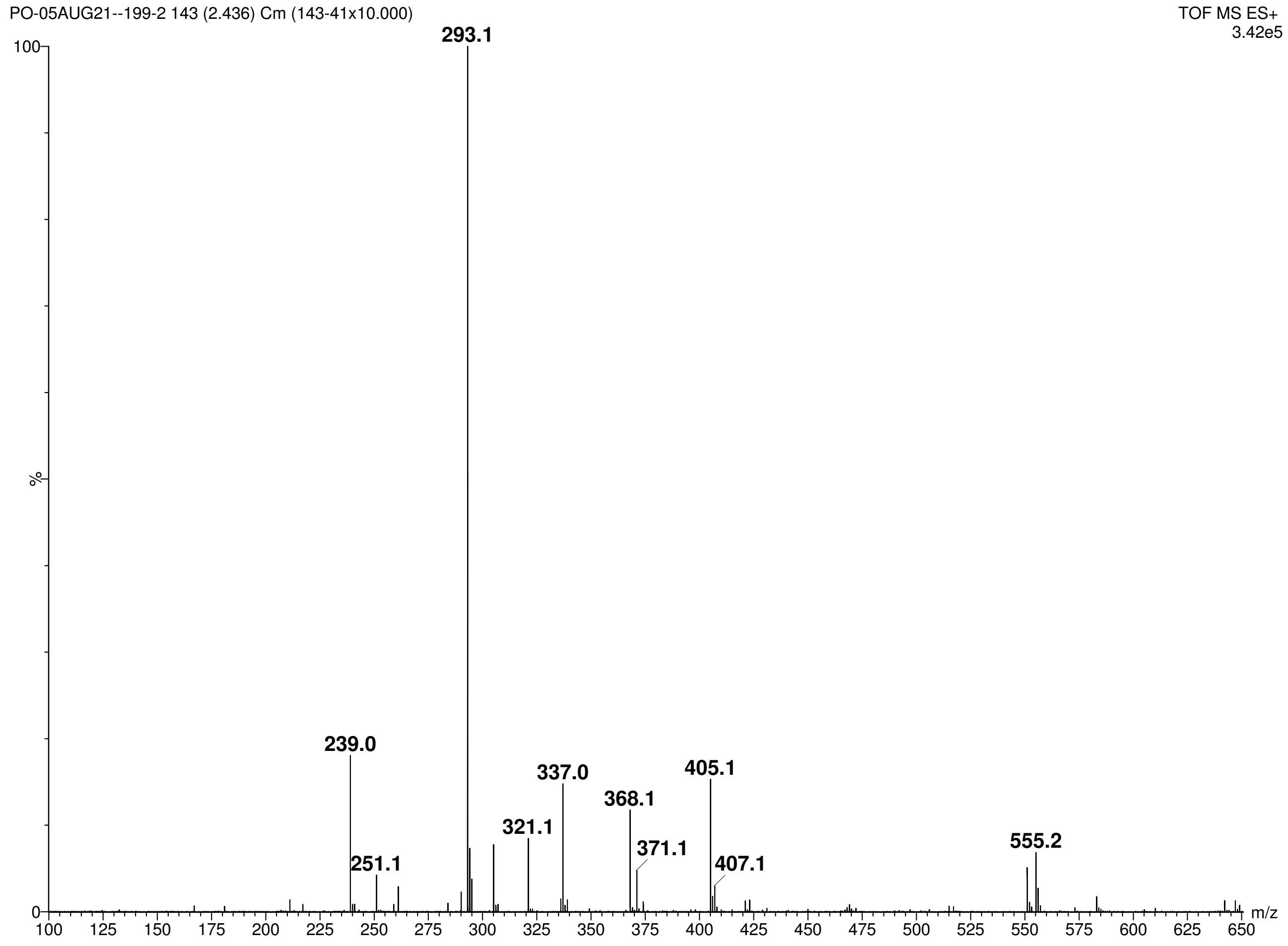

Elemental Composition Report

Page 1

Single Mass Analysis

Tolerance = 5.0 mDa / DBE: min = -1.5, max = 100.0
Element prediction: Off
Number of isotope peaks used for i-FIT = 3

Monoisotopic Mass, Even Electron Ions
26 formula(e) evaluated with 1 results within limits (up to 50 closest results for each mass)
Elements Used:
C: 0-100 H: 0-250 N: 4-4 O: 0-20 32S: 2-2
PO-05AUG21--199-2 143 (2.436) AM2 (Ar,25000.0,0.00,0.00); ABS
TOF MS ES+

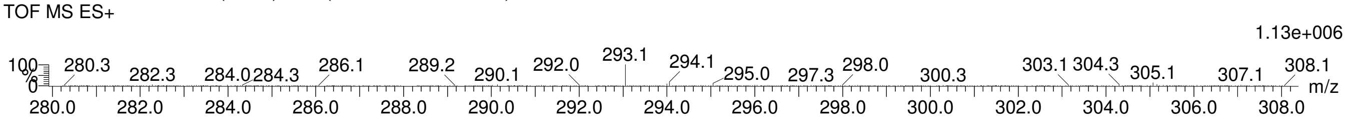

Minimum:				-1.5				
Maximum:		5.0	5.0	100.0				
Mass	Calc. Mass	mDa	PPM	DBE	i-FIT	Norm	Conf(%)	Formula
293.0527	293.0531	-0.4	-1.4	8.5	635.2	n/a	n/a	C12 H13 N4 O 32S2

Data File C:\Chem32\1\Data\HOBIN\HBL199.D
Sample Name: HBL 199 2

HPLC

===

Acq. Operator : ANLYTICAL HPLC Seq. Line : 2
Acq. Instrument : Instrument 1 Location : 2
Injection Date : 8/5/2021 8:04:40 AM Inj : 1
Inj Volume : 100.0 µl
Different Inj Volume from Sample Entry! Actual Inj Volume : 10.0 µl
Acq. Method : C:\Chem32\1\DATA\HOBIN\HOBIN 2021 08 05 10 30 44\HOBIN_AN_(A)ACCN_05 95 (B) TEAA_95 05.M
Last changed : 8/5/2021 7:10:20 AM by ANLYTICAL HPLC
Analysis Method : C:\Chem32\1\Methods\ZW_A10 100_B_20MIN.M
Last changed : 5/27/2021 8:51:31 PM by SYSTEM
Additional Info : Peak(s) manually integrated

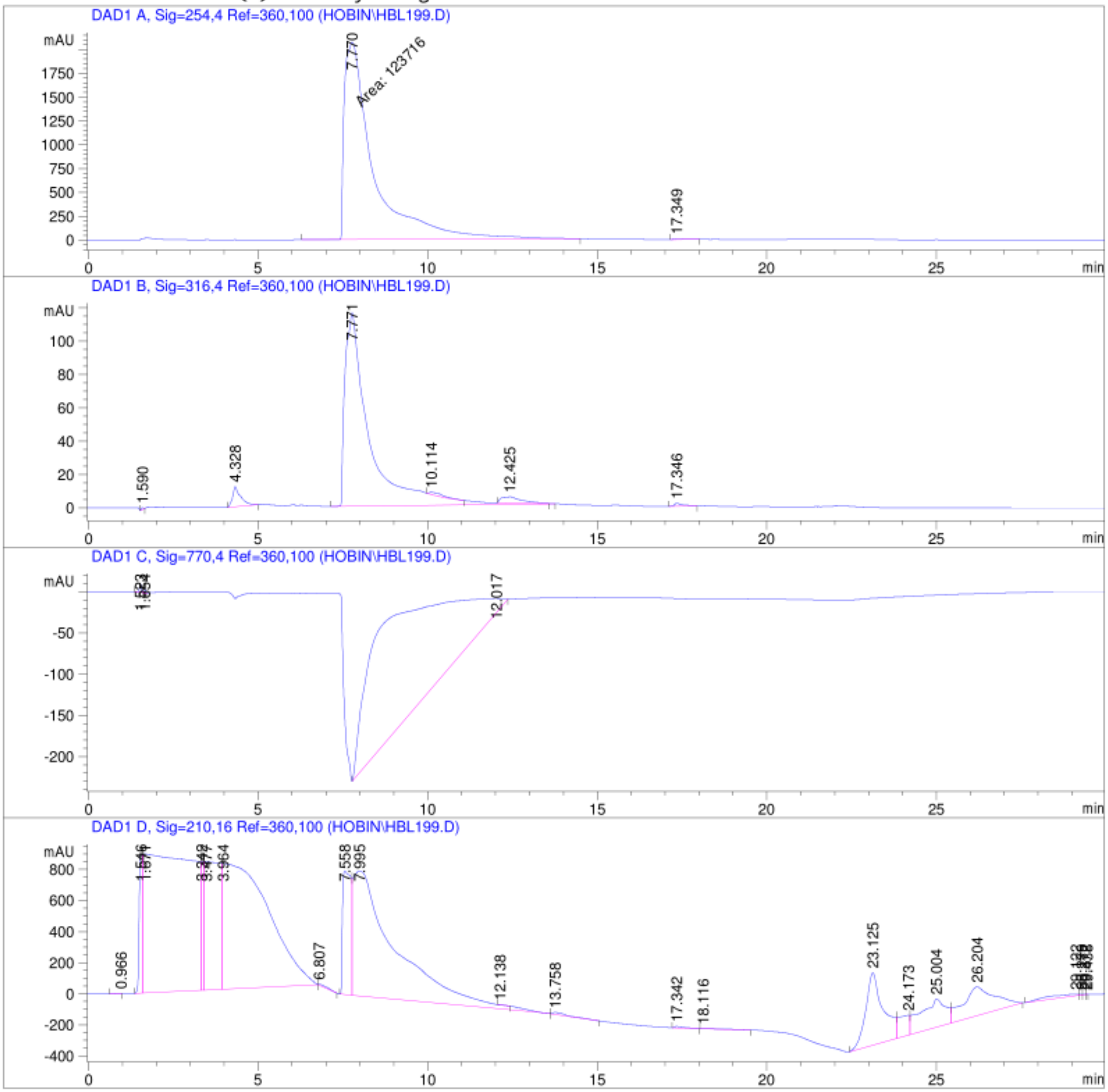

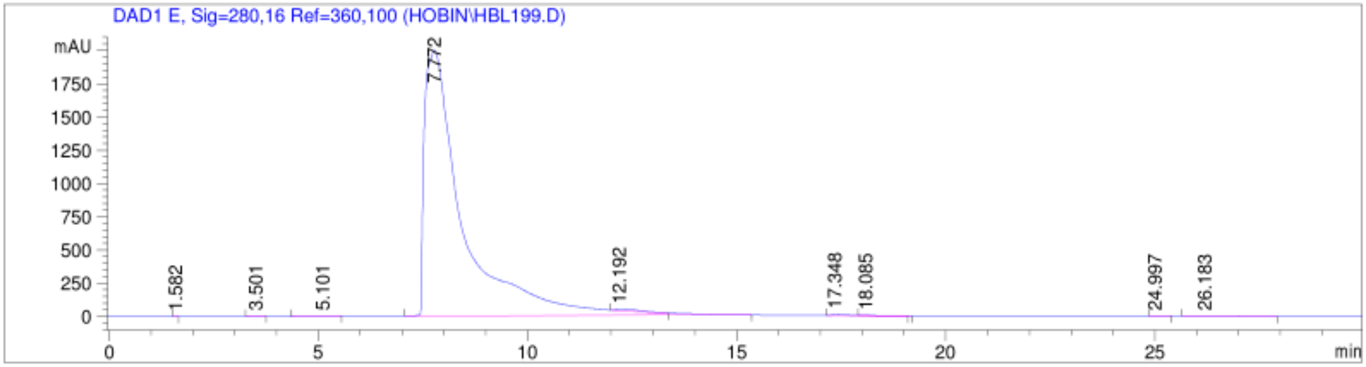

===

Area Percent Report

===

Sorted By : Signal
Multiplier : 1.0000
Dilution : 1.0000
Use Multiplier & Dilution Factor with ISTDs

Signal 1: DAD1 A, Sig=254,4 Ref=360,100

Peak #	RetTime [min]	Type	Width [min]	Area [mAU*s]	Height [mAU]	Area %
1	7.770	MM T	0.9958	1.23716e5	2070.67871	99.8757
2	17.349	BB	0.2581	153.91313	7.87161	0.1243

Totals : 1.23870e5 2078.55032

Signal 2: DAD1 B, Sig=316,4 Ref=360,100

Peak #	RetTime [min]	Type	Width [min]	Area [mAU*s]	Height [mAU]	Area %
1	1.590	BB	0.0779	5.14150	1.11239	0.0821
2	4.328	BB	0.2167	195.83829	11.97964	3.1282
3	7.771	BV R	0.6348	5822.68408	116.16634	93.0069
4	10.114	VV E	0.4446	48.80513	1.39367	0.7796
5	12.425	VB E	0.5128	158.28419	3.74690	2.5283
6	17.346	BB	0.2235	29.73528	1.81140	0.4750

Totals : 6260.48847 136.21035

Signal 3: DAD1 C, Sig=770,4 Ref=360,100

Compound 6

^{1}H NMR spectrum

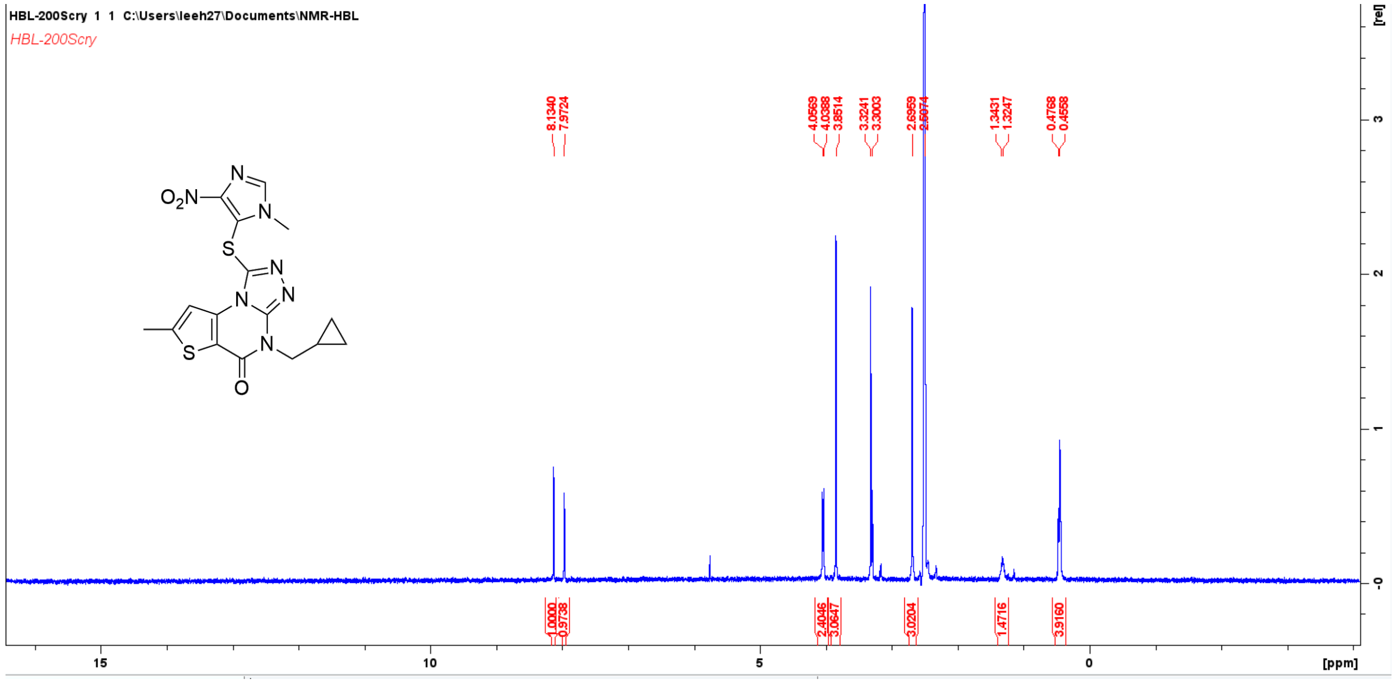

Mass spectrum

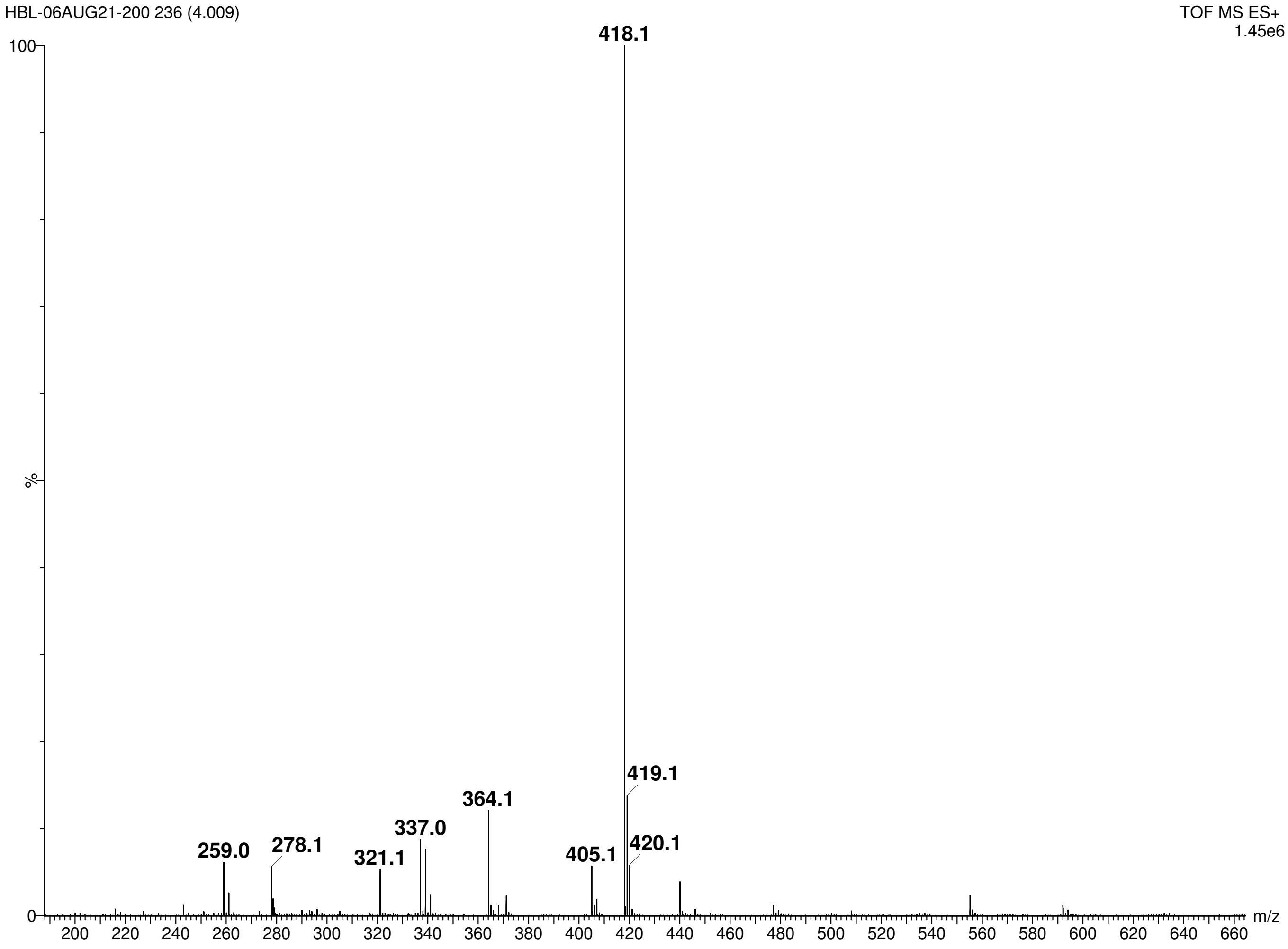

Elemental Composition Report

Single Mass Analysis

Tolerance = 5.0 mDa / DBE: min = -1.5, max = 100.0
Element prediction: Off
Number of isotope peaks used for i-FIT = 3

Monoisotopic Mass, Even Electron Ions
52 formula(e) evaluated with 1 results within limits (up to 50 closest results for each mass)
Elements Used:
C: 0-100 H: 0-250 N: 7-7 O: 0-20 32S: 2-2
HBL-06AUG21-200 229 (3.891) AM2 (Ar,25000.0,0.00,0.00); ABS
TOF MS ES+

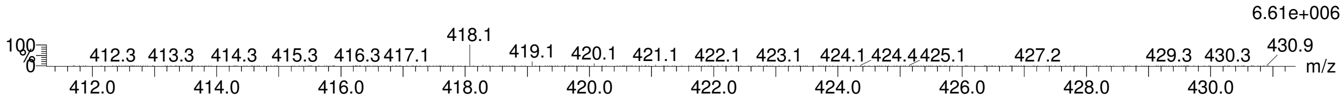

Mass	Calc. Mass	mDa	PPM	DBE	i-FIT	Norm	Conf(%)	Formula
Minimum:				-1.5				
Maximum:		5.0	5.0	100.0				
418.0757	418.0756	0.1	0.2	12.5	497.5	n/a	n/a	C16 H16 N7 O3 32S2

Data File C:\Chem32\1\Data\HOBIN\HBL08060000002.D
Sample Name: HBL 200S

HPLC

===

Acq. Operator : ANLYTICAL HPLC   Seq. Line : 1
Acq. Instrument : Instrument 1   Location : 4
Injection Date : 8/6/2021 7:32:52 AM   Inj : 1
Inj Volume : 100.0 µl
Different Inj Volume from Sample Entry! Actual Inj Volume : 20.0 µl
Acq. Method : C:\Chem32\1\DATA\HOBIN\HOBIN 2021 08 06 10 21 47\HOBIN_AN_(A)ACCN_05 95 (B) TEAA_95 05.M
Last changed : 8/5/2021 7:10:20 AM by ANLYTICAL HPLC
Analysis Method : C:\Chem32\1\Methods\HBL_A05 95_B95 05_20MIN.M
Last changed : 9/15/2021 2:37:26 PM by SYSTEM
Additional Info : Peak(s) manually integrated

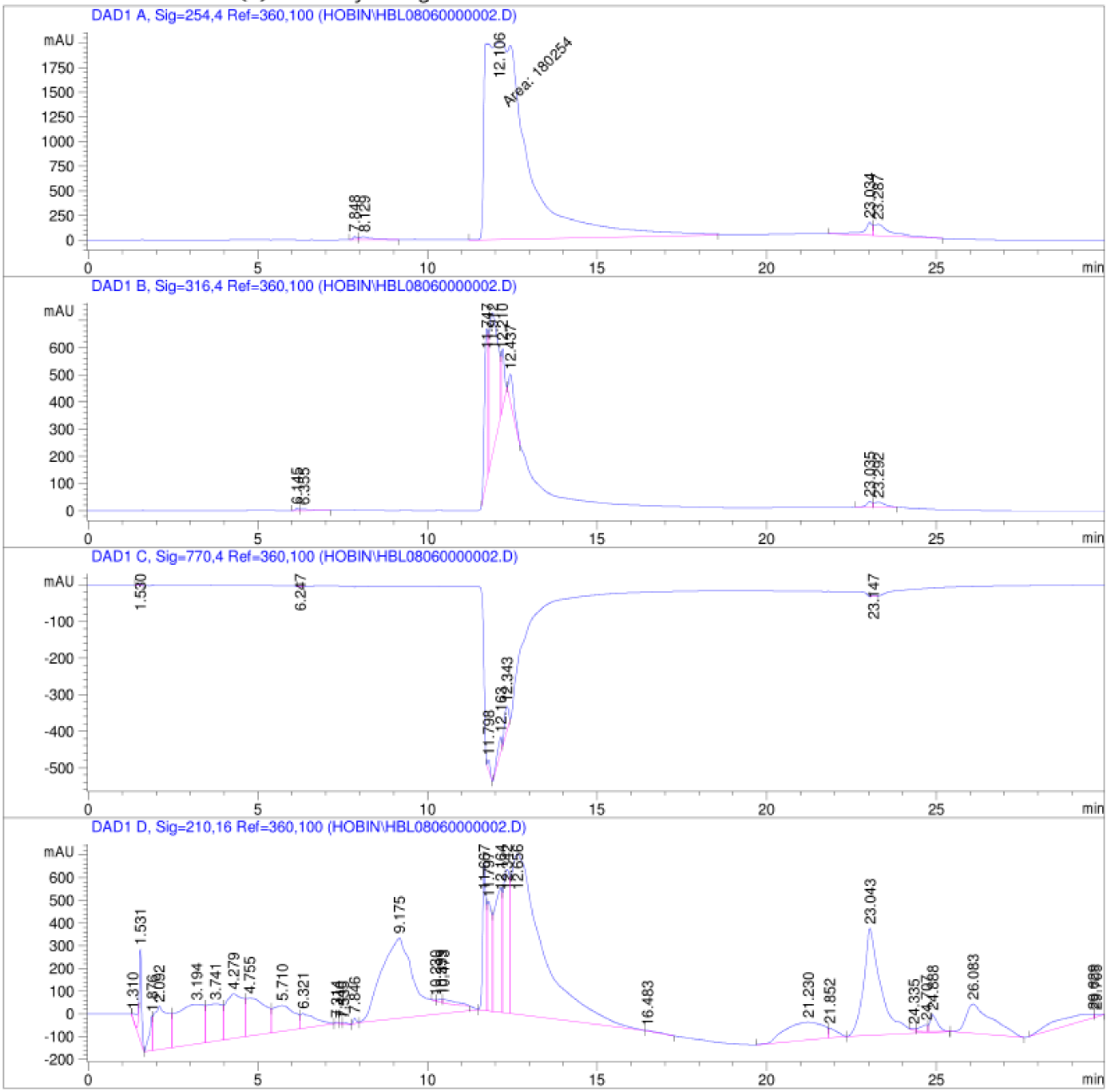

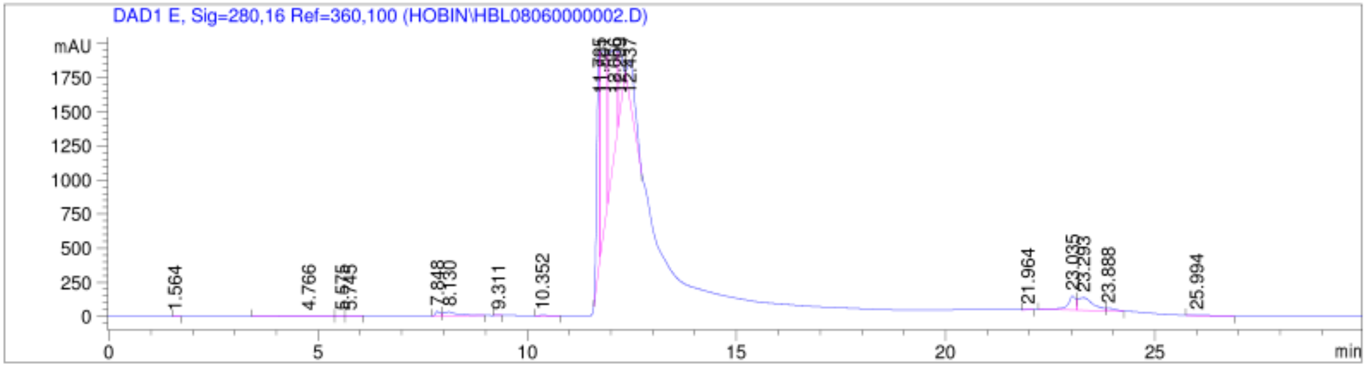

===

Area Percent Report

===

Sorted By : Signal
Multiplier : 1.0000
Dilution : 1.0000
Use Multiplier & Dilution Factor with ISTDs

Signal 1: DAD1 A, Sig=254,4 Ref=360,100

Peak #	RetTime [min]	Type	Width [min]	Area [mAU*s]	Height [mAU]	Area %
1	7.848	BV	0.1113	245.57950	32.09408	0.1307
2	8.129	VV R	0.3223	712.78003	28.06906	0.3794
3	12.106	MM T	1.5008	1.80254e5	2001.72656	95.9407
4	23.034	VV R	0.2618	2624.13794	131.62860	1.3967
5	23.287	VB	0.4812	4044.07300	115.30089	2.1525

Totals : 1.87880e5 2308.81919

Signal 2: DAD1 B, Sig=316,4 Ref=360,100

Peak #	RetTime [min]	Type	Width [min]	Area [mAU*s]	Height [mAU]	Area %
1	6.145	BV	0.1073	47.09761	6.44281	0.2836
2	6.355	VB	0.2532	96.95863	5.11299	0.5839
3	11.747	BV	0.1197	4296.05615	558.66278	25.8729
4	11.912	VV	0.2428	9083.35449	522.52069	54.7043
5	12.210	VB	0.0868	1321.07043	217.60759	7.9561
6	12.437	BB	0.1716	1132.99353	100.47794	6.8234
7	23.035	BV	0.1679	246.95720	20.89854	1.4873
8	23.292	VB	0.3207	379.95911	17.82144	2.2883

Totals : 1.66044e4 1449.54478

Compound 8

^1H NMR spectrum

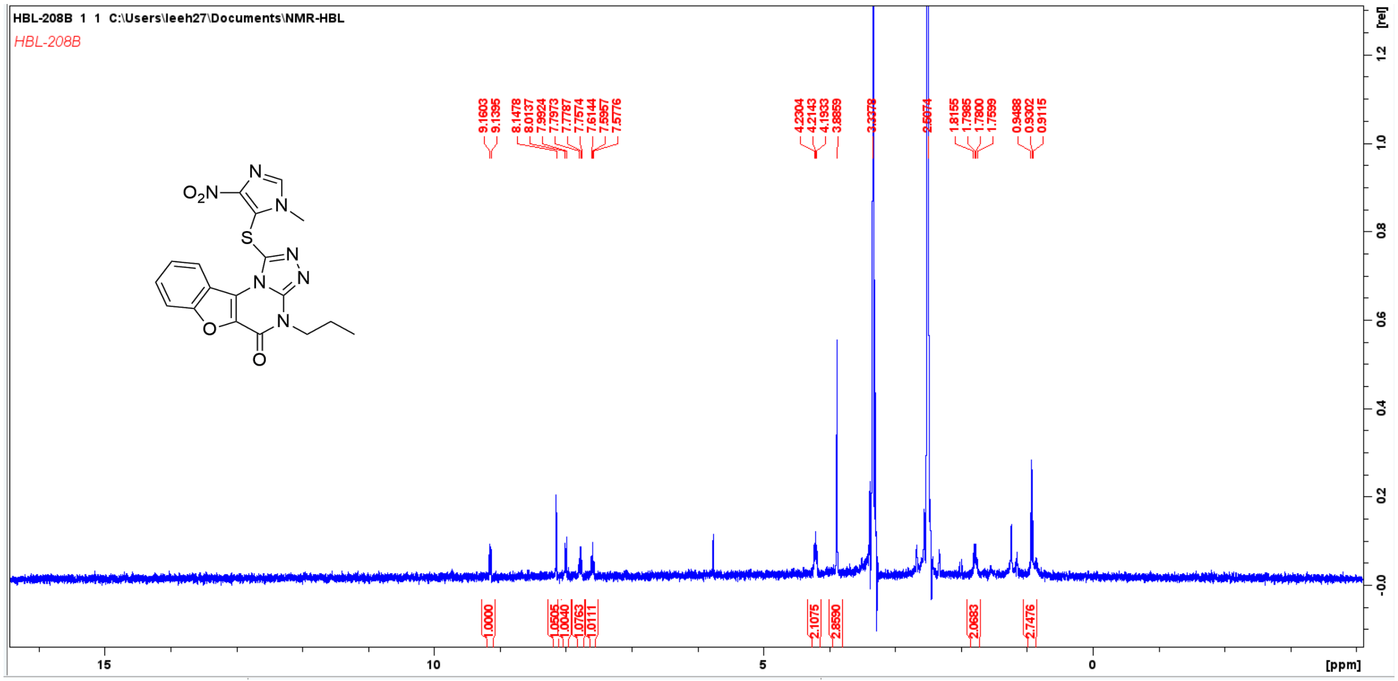

Mass spectrum

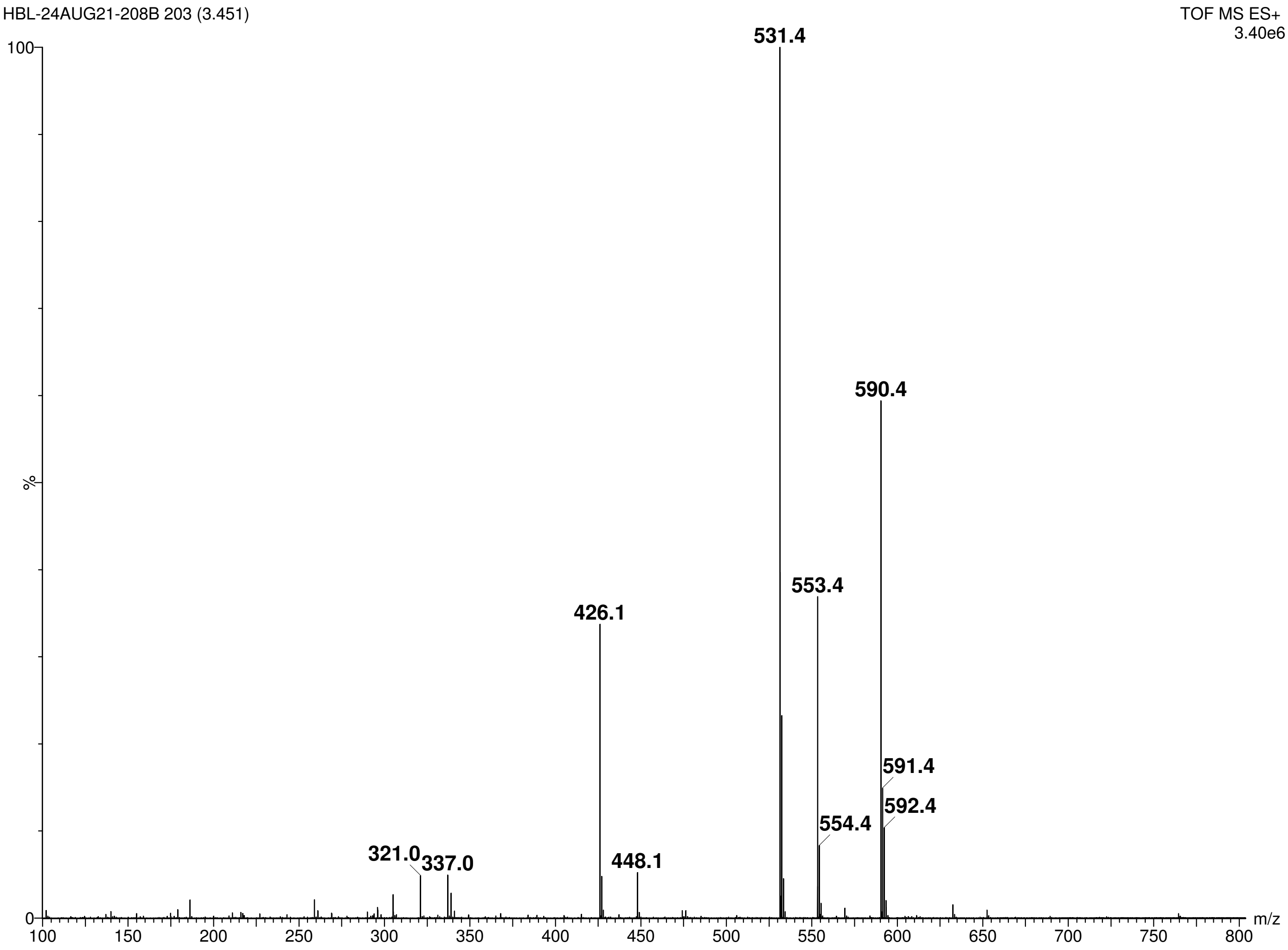

Elemental Composition Report

Page 1

Single Mass Analysis

Tolerance = 5.0 mDa / DBE: min = -1.5, max = 100.0
Element prediction: Off
Number of isotope peaks used for i-FIT = 3

Monoisotopic Mass, Even Electron Ions
63 formula(e) evaluated with 1 results within limits (up to 50 closest results for each mass)
Elements Used:
C: 0-100 H: 0-250 N: 7-7 O: 0-20 32S: 1-1
HBL-24AUG21-208B 200 (3.400) AM2 (Ar,25000.0,0.00,0.00); ABS
TOF MS ES+

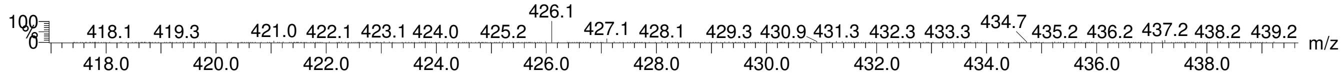

Minimum:				-1.5				
Maximum:		5.0	5.0	100.0				
Mass	Calc. Mass	mDa	PPM	DBE	i-FIT	Norm	Conf(%)	Formula
426.0978	426.0984	-0.6	-1.4	14.5	437.8	n/a	n/a	C18 H16 N7 O4 32S

Data File C:\Chem32\1\Data\HOBIN\HBL08311.D
Sample Name: HBL208

HPLC

===

Acq. Operator : PAOLA Seq. Line : 1
Acq. Instrument : Instrument 1 Location : 15
Injection Date : 8/31/2021 2:20:03 PM Inj : 1
Inj Volume : 100.000 µl
Different Inj Volume from Sample Entry! Actual Inj Volume : 8.000 µl
Acq. Method : C:\HPCHEM\1\METHODS\HBLA5T95.M
Last changed : 8/27/2021 9:11:28 AM by PAOLA
Analysis Method : C:\Chem32\1\Methods\HBL_A05 95_B95 05_20MIN.M
Last changed : 9/15/2021 2:37:26 PM by SYSTEM
Additional Info : Peak(s) manually integrated

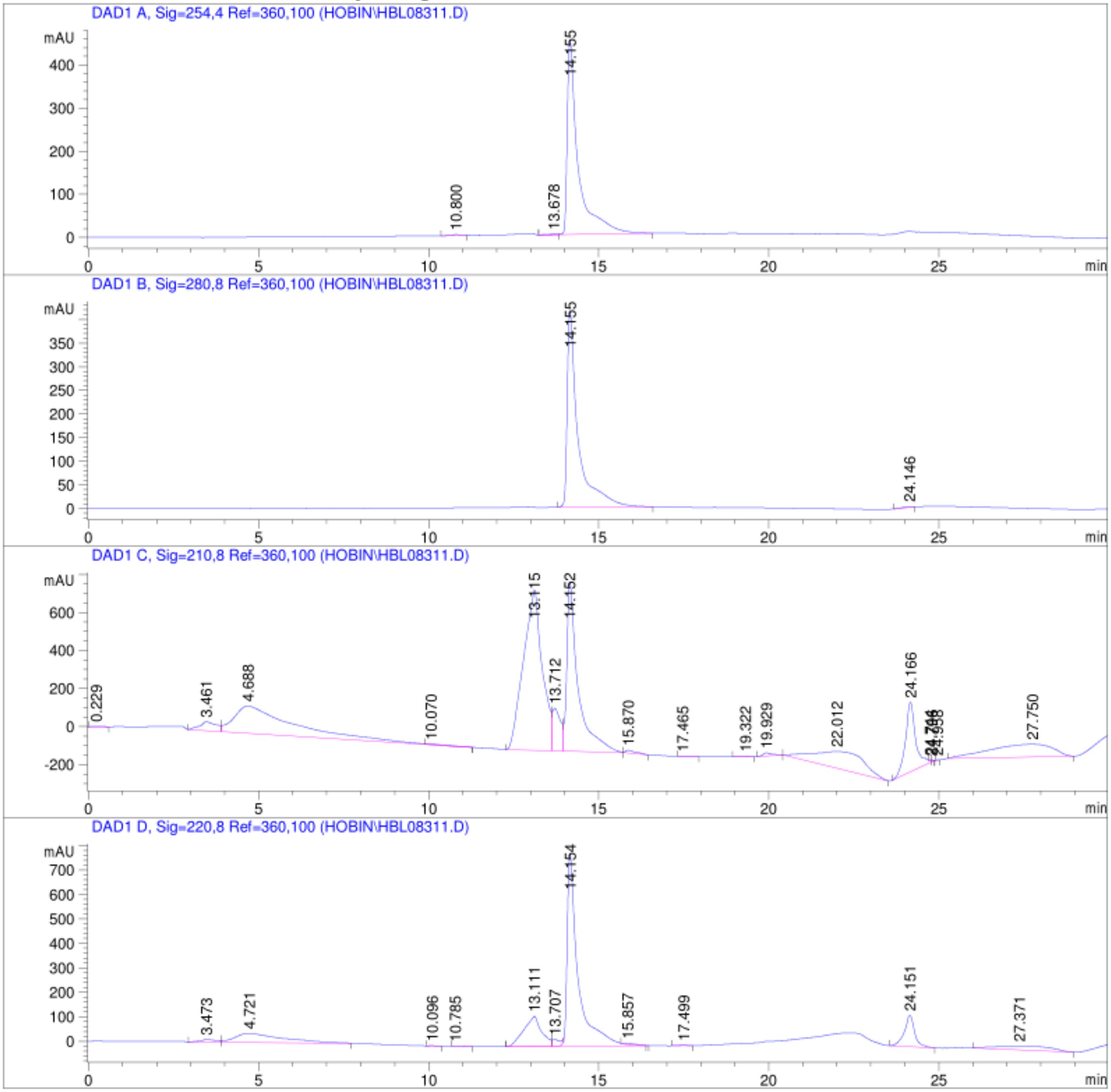

===

Area Percent Report

===

Sorted By : Signal
Multiplier : 1.0000
Dilution : 1.0000
Use Multiplier & Dilution Factor with ISTDs

Signal 1: DAD1 A, Sig=254,4 Ref=360,100

Peak #	RetTime [min]	Type	Width [min]	Area [mAU*s]	Height [mAU]	Area %
1	10.800	BB	0.1977	28.38407	2.07339	0.2773
2	13.678	BV E	0.2913	29.58100	1.28139	0.2890
3	14.155	VB R	0.3170	1.01787e4	451.58386	99.4337

Totals : 1.02366e4 454.93864

Signal 2: DAD1 B, Sig=280,8 Ref=360,100

Peak #	RetTime [min]	Type	Width [min]	Area [mAU*s]	Height [mAU]	Area %
1	14.155	BB	0.3168	9344.57813	414.83142	99.7047
2	24.146	BB	0.2381	27.67387	1.49382	0.2953

Totals : 9372.25199 416.32524

Signal 3: DAD1 C, Sig=210,8 Ref=360,100

Peak #	RetTime [min]	Type	Width [min]	Area [mAU*s]	Height [mAU]	Area %
1	0.229	BB	0.3056	43.48315	2.22906	0.0425
2	3.461	BV	0.4686	1624.90173	46.85941	1.5888
3	4.688	VV R	1.5930	1.75619e4	142.98347	17.1714
4	10.070	VB E	0.5495	139.34212	3.24152	0.1362
5	13.115	BV	0.5988	3.22389e4	841.79730	31.5219
6	13.712	VV	0.2436	3837.44458	222.02841	3.7521
7	14.152	VB	0.3238	2.05023e4	886.58844	20.0464
8	15.870	BB	0.3017	285.58682	13.66956	0.2792
9	17.465	BB	0.2148	24.83601	1.78018	0.0243
10	19.322	BB	0.2314	17.56840	1.03857	0.0172
11	19.929	BB	0.2549	267.70972	15.24454	0.2618
12	22.012	BB	1.2910	9430.67676	87.24854	9.2209
13	24.166	BV R	0.3018	7655.67139	366.41086	7.4854
14	24.744	VV E	0.0434	12.05203	4.21610	0.0118
15	24.796	VB E	0.0524	15.40728	4.44710	0.0151
16	24.958	BV	0.0681	8.74142	1.95668	8.547e 3
17	27.750	BB	1.5358	8607.87305	66.25198	8.4164

Compound 10

^{1}H NMR spectrum

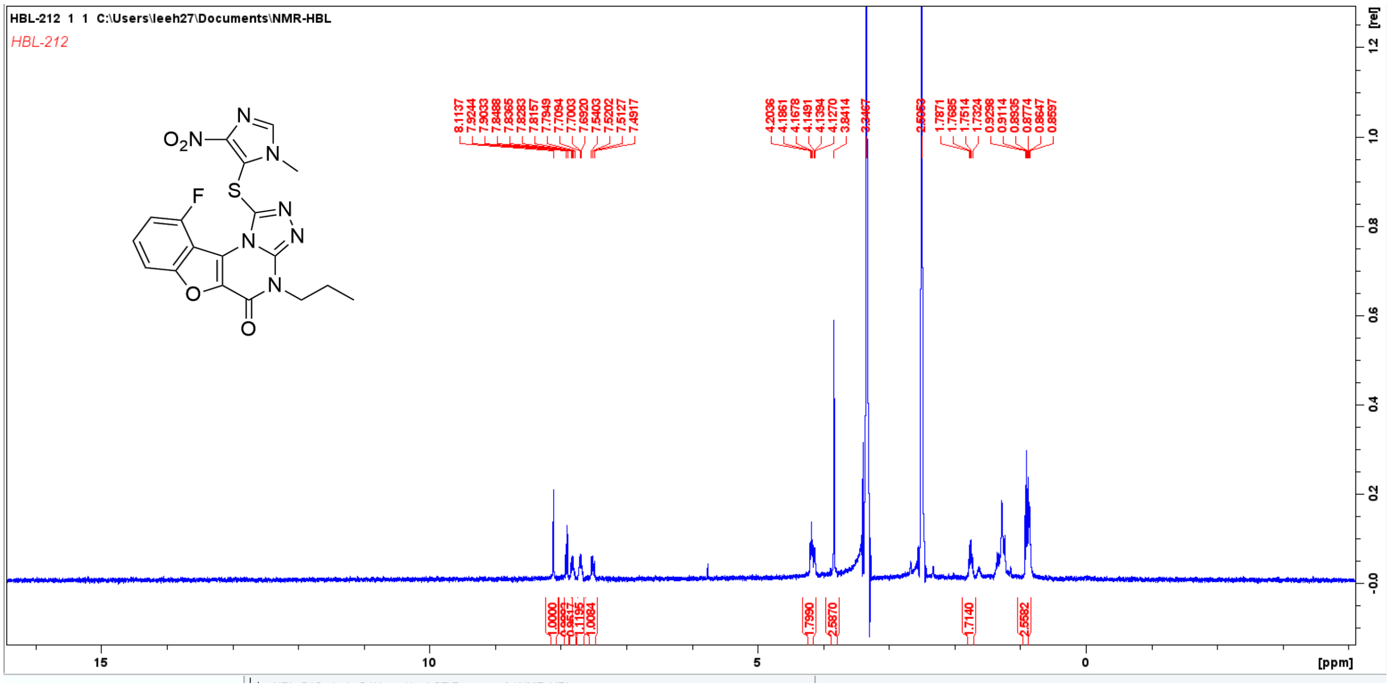

^{19}F NMR spectrum

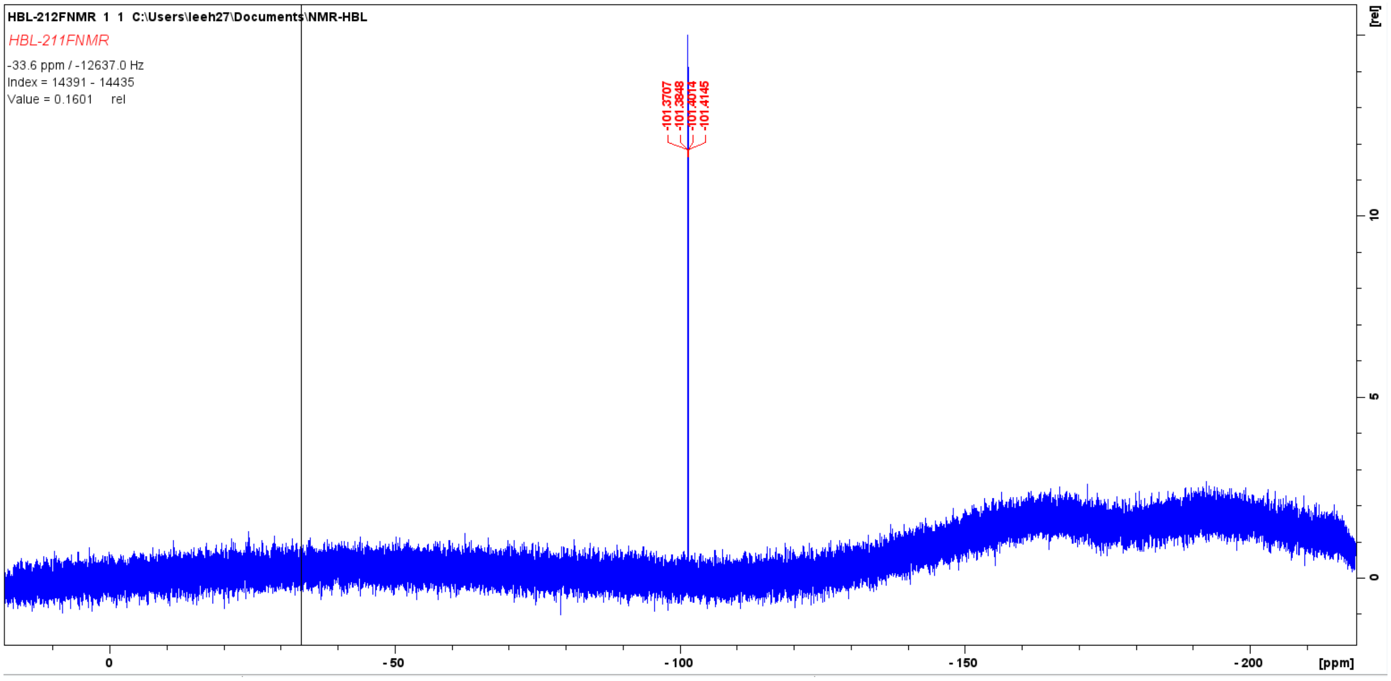

Mass spectrum

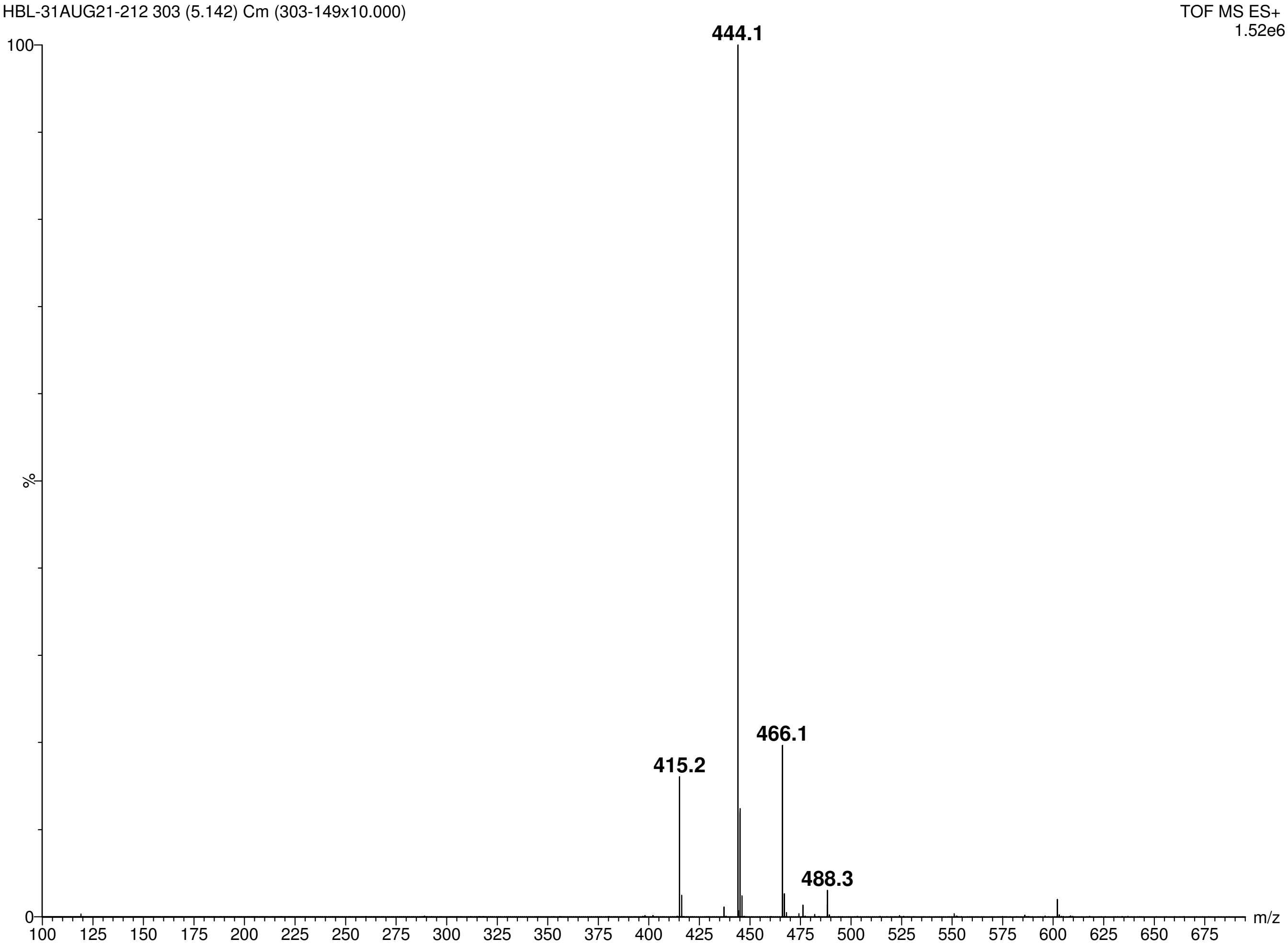

Elemental Composition Report

Single Mass Analysis

Tolerance = 3.0 PPM / DBE: min = -1.5, max = 100.0
Element prediction: Off
Number of isotope peaks used for i-FIT = 3

Monoisotopic Mass, Even Electron Ions
2513 formula(e) evaluated with 8 results within limits (up to 50 closest results for each mass)
Elements Used:
C: 0-100 H: 0-250 N: 3-8 O: 0-20 F: 0-2 32S: 0-1
HBL-31AUG21-212 300 (5.092) AM2 (Ar,25000.0,0.00,0.00); ABS
TOF MS ES+

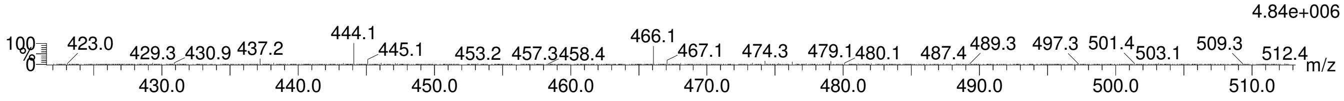

Minimum:				-1.5				
Maximum:		5.0	3.0	100.0				
Mass	Calc. Mass	mDa	PPM	DBE	i-FIT	Norm	Conf(%)	Formula
444.0900	444.0902	-0.2	-0.5	5.5	485.3	2.819	5.97	C13 H19 N3 O13 F
	444.0902	-0.2	-0.5	10.5	485.5	3.026	4.85	C15 H16 N7 O5 F2 32S
	444.0897	0.3	0.7	23.5	487.3	4.886	0.76	C26 H11 N5 O2 F
	444.0904	-0.4	-0.9	14.5	483.8	1.360	25.68	C17 H14 N7 O8
	444.0908	-0.8	-1.8	19.5	486.4	3.974	1.88	C23 H12 N5 O3 F2
	444.0890	1.0	2.3	9.5	483.2	0.724	48.47	C16 H18 N3 O12
	444.0890	1.0	2.3	14.5	485.0	2.583	7.55	C18 H15 N7 O4 F 32S
	444.0888	1.2	2.7	5.5	485.5	3.027	4.85	C14 H20 N3 O9 F2 32S

HPLC

Data File C:\Chem32\1\Data\PAOLA\Paola 2023-03-07 12-40-34\NCK1840000001.D
Sample Name: NCK184

==

Acq. Operator : SYSTEM Seq. Line : 1
Acq. Instrument : hplc Location : 13
Injection Date : 3/7/2023 12:44:30 PM Inj : 1
Inj Volume : 100.000 µl
Different Inj Volume from Sample Entry! Actual Inj Volume : 5.000 µl
Sequence File : C:\Chem32\1\Data\PAOLA\Paola 2023-03-07 12-40-34\Paola.S
Method : C:\Chem32\1\Data\PAOLA\Paola 2023-03-07 12-40-34\HBL1_A05-95_B95-05_20MIN.M
(Sequence Method)
Last changed : 3/7/2023 12:40:34 PM by SYSTEM

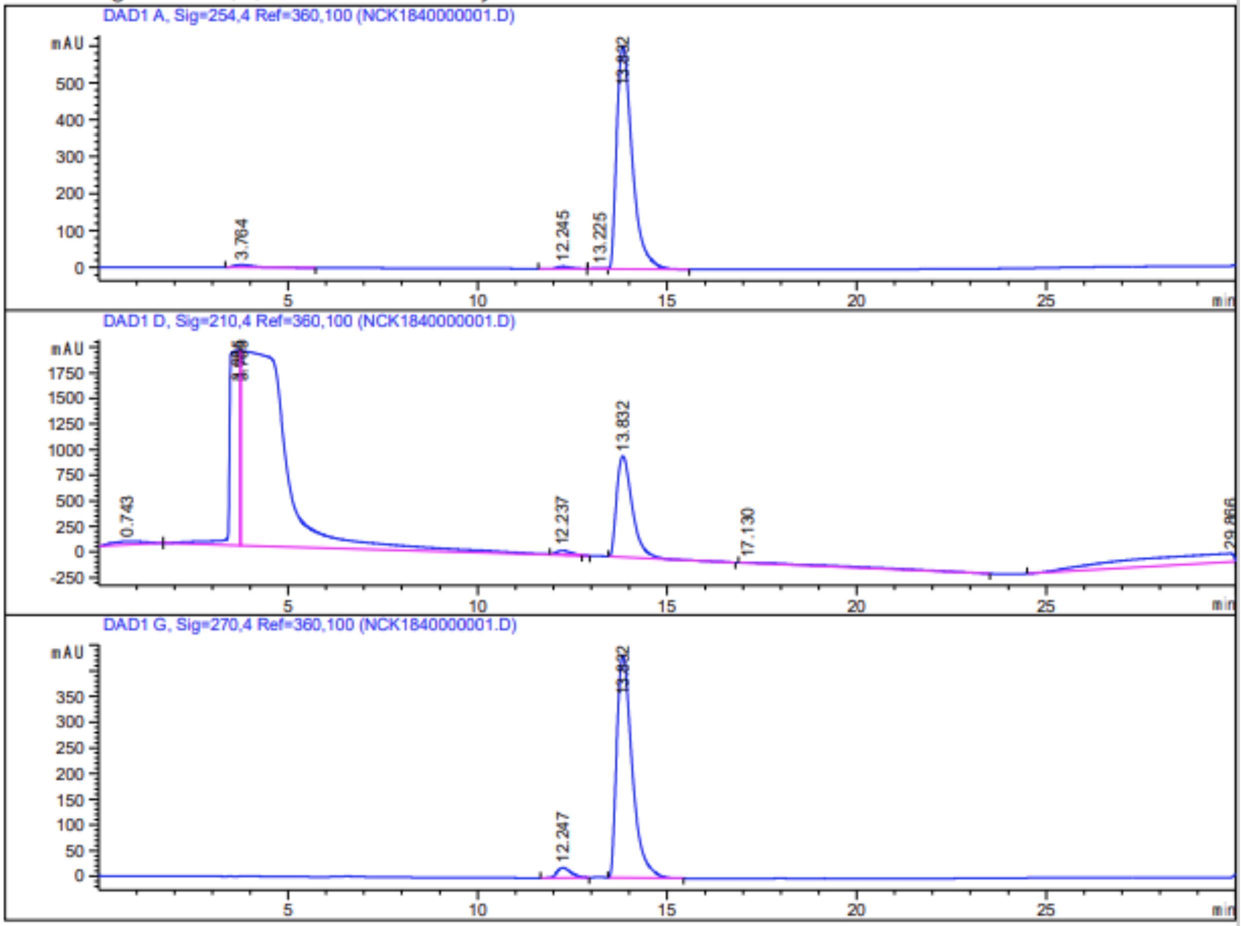

===

Fraction Information

===

Fraction collection using a timetable

===

No Fractions found.

===

===

Area Percent Report

===

Sorted By : Signal
Multiplier : 1.0000
Dilution : 1.0000
Sample Amount: : 1.00000 [ng/ul] (not used in calc.)
Use Multiplier & Dilution Factor with ISTDs

Signal 1: DAD1 A, Sig=254,4 Ref=360,100

Peak #	RetTime [min]	Type	Width [min]	Area [mAU*s]	Height [mAU]	Area %
1	3.764	BB	0.5426	288.93622	7.61549	1.6349
2	12.245	BB	0.3845	162.81548	6.40566	0.9213
3	13.225	BV E	0.2933	32.34383	1.70497	0.1830
4	13.832	VB R	0.4443	1.71892e4	603.51776	97.2609

Totals : 1.76733e4 619.24388

Signal 2: DAD1 D, Sig=210,4 Ref=360,100

Peak #	RetTime [min]	Type	Width [min]	Area [mAU*s]	Height [mAU]	Area %
1	0.743	BB	0.7481	2267.07349	38.88858	0.9205
2	3.685	BV	0.2264	3.55594e4	1899.62988	14.4378
3	3.763	VV R	1.3749	1.56620e5	1898.52173	63.5909
4	12.237	VB E	0.3812	930.66632	38.61475	0.3779
5	13.832	BB	0.4548	2.86445e4	986.26959	11.6302
6	17.130	BB	6.1626	2843.26709	5.44096	1.1544
7	29.866	BBA	2.8467	1.94284e4	80.87397	7.8883

Totals : 2.46294e5 4948.23947

Signal 3: DAD1 G, Sig=270,4 Ref=360,100

Peak #	RetTime [min]	Type	Width [min]	Area [mAU*s]	Height [mAU]	Area %
1	12.247	BB	0.4048	493.79620	19.40805	3.8595
2	13.832	BB	0.4431	1.23005e4	433.38617	96.1405

Totals : 1.27943e4 452.79422

===

*** End of Report ***

Compound 12

^{1}H NMR spectrum

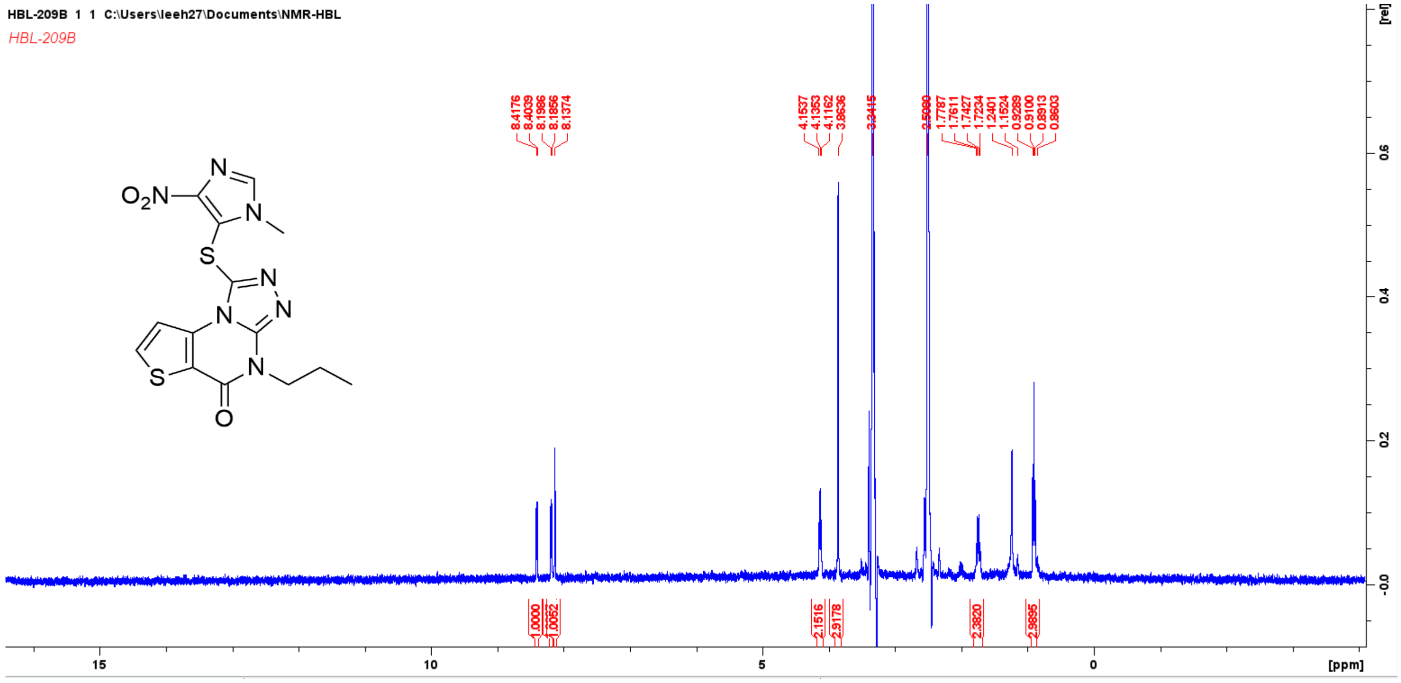

Mass spectrum

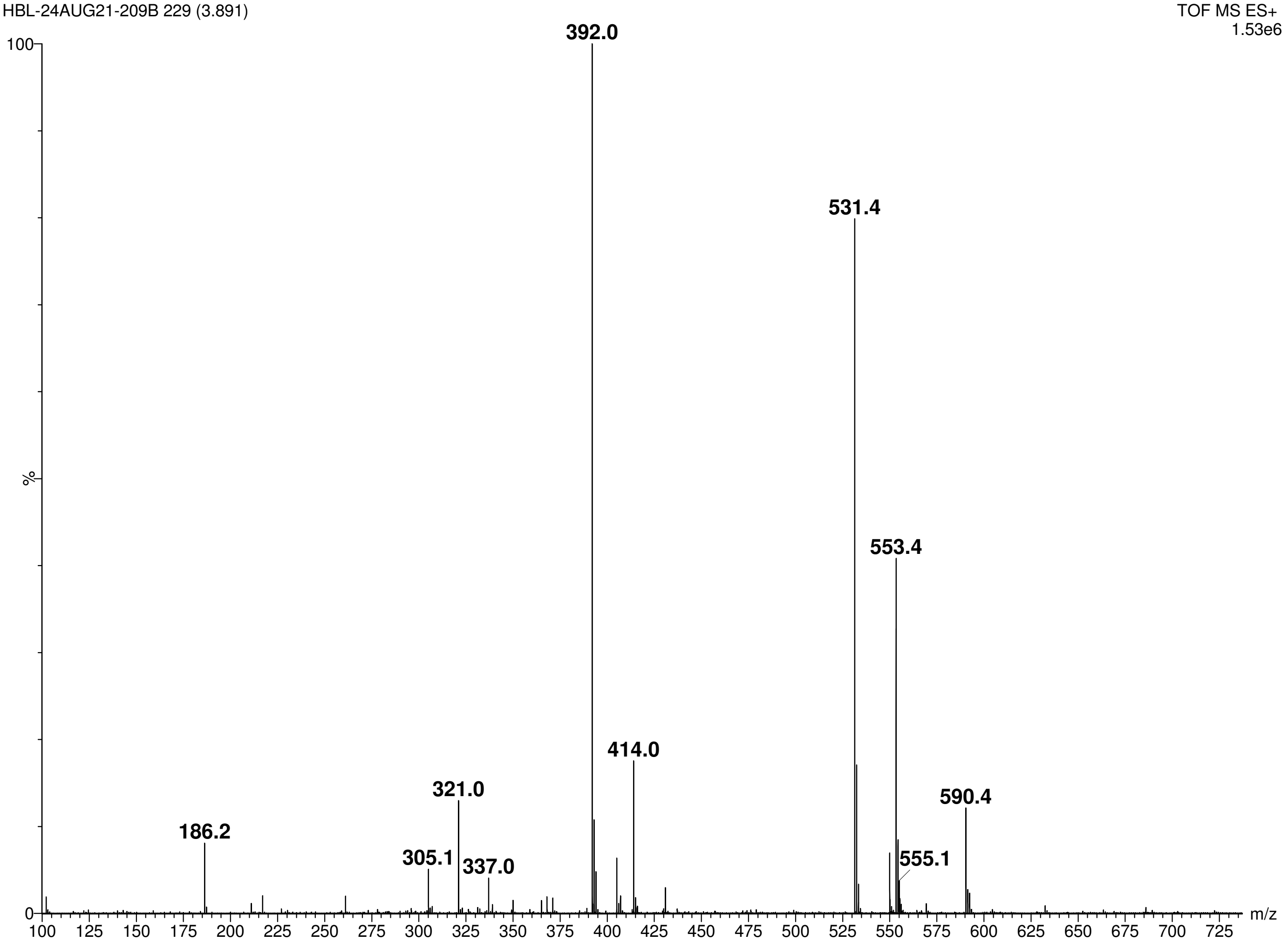

Elemental Composition Report

Single Mass Analysis

Tolerance = 5.0 mDa / DBE: min = -1.5, max = 100.0
Element prediction: Off
Number of isotope peaks used for i-FIT = 3

Monoisotopic Mass, Even Electron Ions
41 formula(e) evaluated with 1 results within limits (up to 50 closest results for each mass)
Elements Used:
C: 0-100 H: 0-250 N: 7-7 O: 0-20 32S: 2-2
HBL-24AUG21-209B 226 (3.840) AM2 (Ar,25000.0,0.00,0.00); ABS
TOF MS ES+

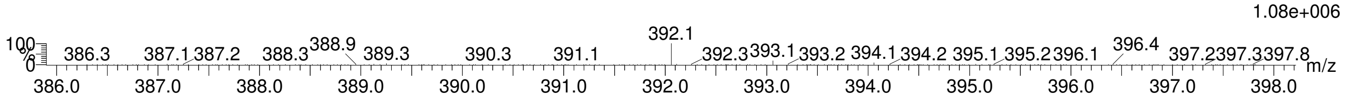

Minimum:				-1.5				
Maximum:		5.0	5.0	100.0				
Mass	Calc. Mass	mDa	PPM	DBE	i-FIT	Norm	Conf(%)	Formula
392.0601	392.0600	0.1	0.3	11.5	468.3	n/a	n/a	C14 H14 N7 O3 32S2

Data File C:\Chem32\1\Data\HOBIN\HBL08274.D
Sample Name: HBL209

HPLC

===

Acq. Operator : PAOLA Seq. Line : 4
Acq. Instrument : Instrument 1 Location : 14
Injection Date : 8/27/2021 10:57:57 AM Inj : 1
Inj Volume : 100.000 µl
Different Inj Volume from Sample Entry! Actual Inj Volume : 8.000 µl
Acq. Method : C:\HPCHEM\1\METHODS\HBLA5T95.M
Last changed : 8/27/2021 9:11:28 AM by PAOLA
Analysis Method : C:\Chem32\1\Methods\HBL_A05 95_B95 05_20MIN.M
Last changed : 9/15/2021 2:37:26 PM by SYSTEM
Additional Info : Peak(s) manually integrated

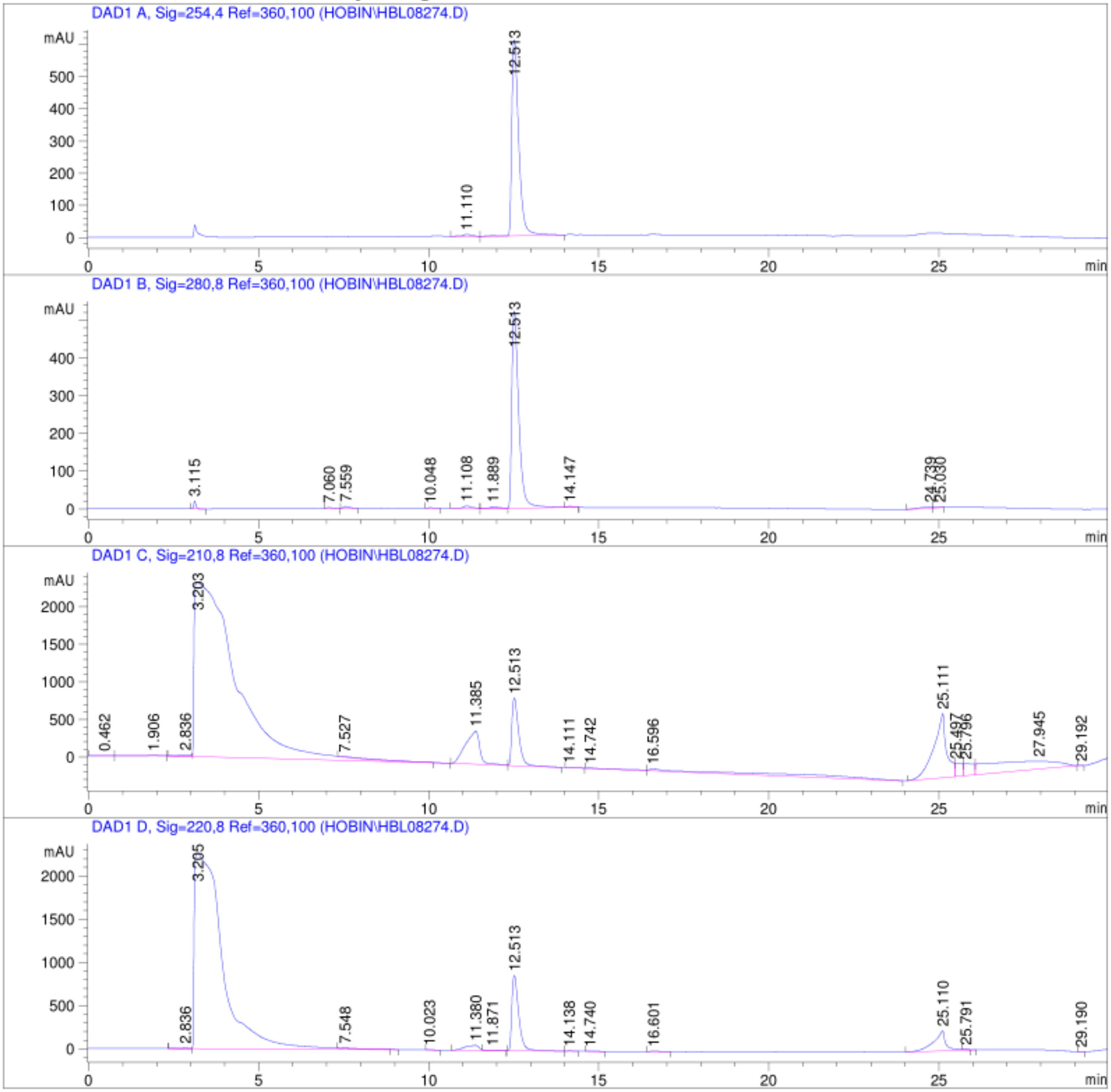

===

Area Percent Report

===

Sorted By : Signal
Multiplier : 1.0000
Dilution : 1.0000
Use Multiplier & Dilution Factor with ISTDs

Signal 1: DAD1 A, Sig=254,4 Ref=360,100

Peak #	RetTime [min]	Type	Width [min]	Area [mAU*s]	Height [mAU]	Area %
1	11.110	BB	0.2795	127.15454	6.46475	1.4332
2	12.513	BV	0.2174	8744.75391	609.45917	98.5668

Totals : 8871.90845 615.92392

Signal 2: DAD1 B, Sig=280,8 Ref=360,100

Peak #	RetTime [min]	Type	Width [min]	Area [mAU*s]	Height [mAU]	Area %
1	3.115	BB	0.0620	87.51526	21.25585	1.1018
2	7.060	BB	0.1650	22.41096	2.09111	0.2821
3	7.559	BB	0.1798	62.09456	5.18105	0.7818
4	10.048	BB	0.1829	14.91207	1.28994	0.1877
5	11.108	BB	0.2512	103.80964	6.14308	1.3069
6	11.889	BV E	0.2521	68.18967	3.89794	0.8585
7	12.513	VV R	0.2159	7425.92090	522.10693	93.4903
8	14.147	VB E	0.1971	32.20908	2.66294	0.4055
9	24.739	BV	0.3368	87.60110	3.14612	1.1029
10	25.030	VB	0.2183	38.32257	2.25493	0.4825

Totals : 7942.98581 570.02988

Signal 3: DAD1 C, Sig=210,8 Ref=360,100

Peak #	RetTime [min]	Type	Width [min]	Area [mAU*s]	Height [mAU]	Area %
1	0.462	BB	0.3849	67.01169	2.61584	0.0240
2	1.906	BB	0.5702	173.45657	4.04540	0.0621
3	2.836	BV E	0.2596	427.83337	21.92733	0.1531
4	3.203	VV R	1.0327	1.96971e5	2319.63403	70.4906
5	7.527	VB E	0.9671	593.92560	7.45790	0.2126
6	11.385	BV	0.4847	1.29422e4	443.21756	4.6317
7	12.513	VB	0.2146	1.26934e4	899.60077	4.5426
8	14.111	BB	0.2121	44.58476	3.37602	0.0160
9	14.742	BV	0.7094	377.38416	6.59663	0.1351
10	16.596	VB	3.5726	8866.43066	29.39878	3.1731

Compound 14

^{1}H NMR spectrum

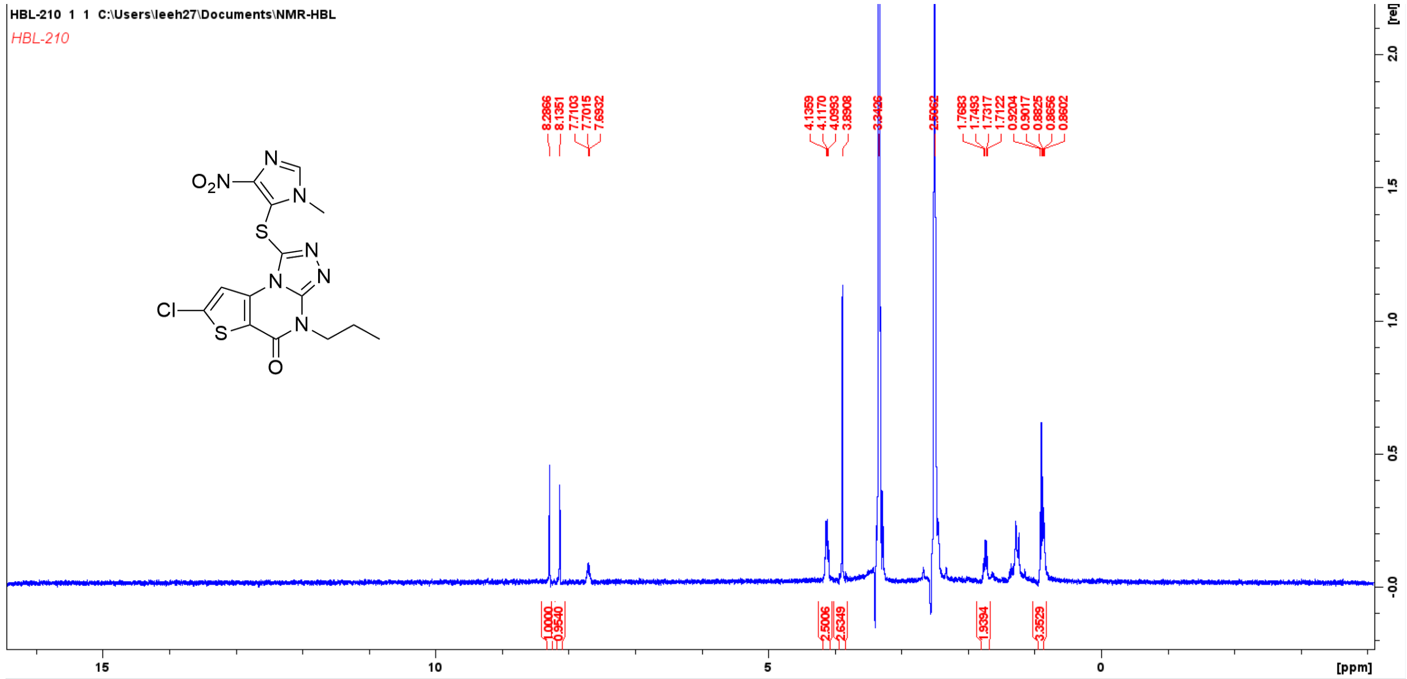

Mass spectrum

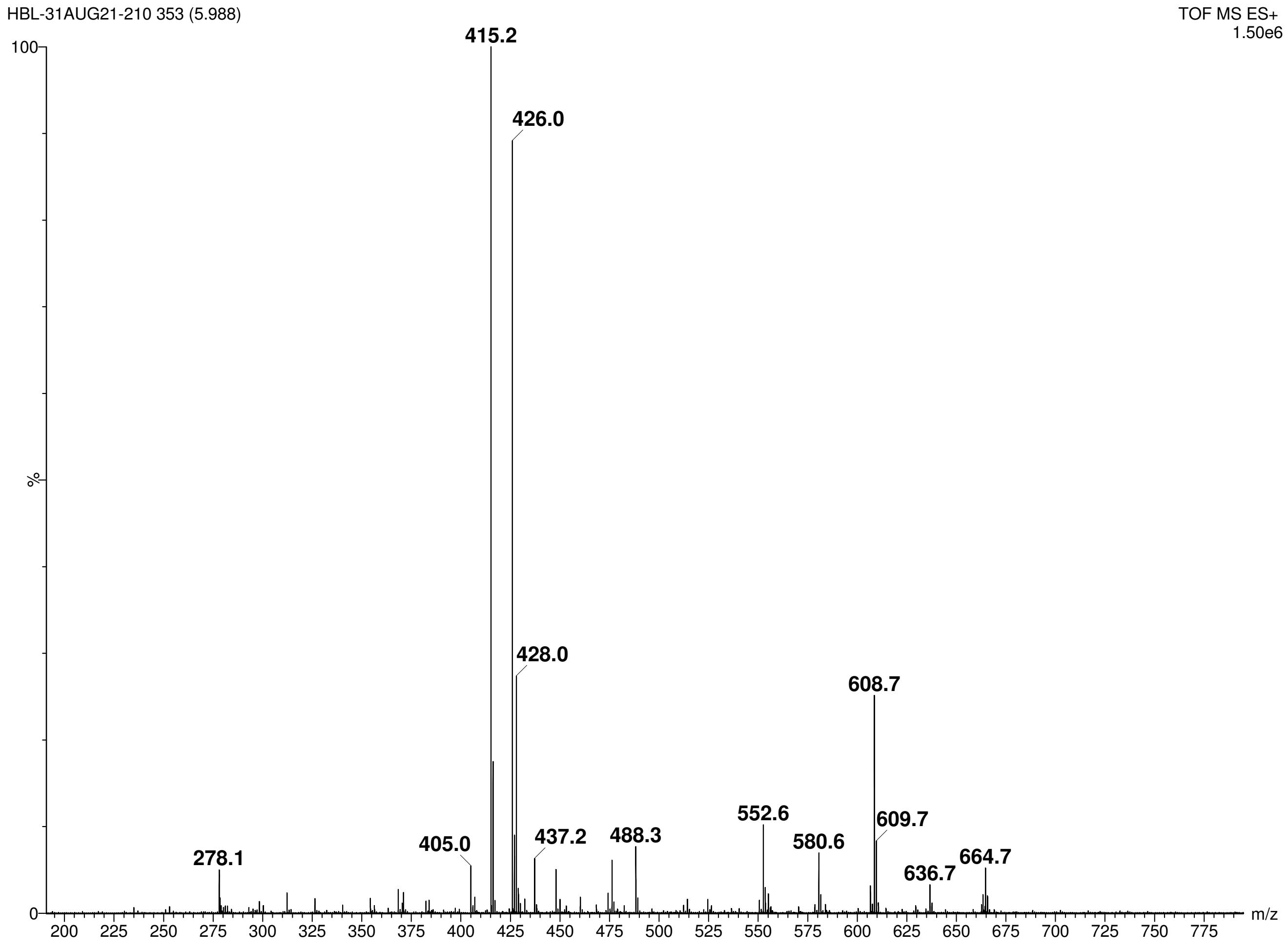

Elemental Composition Report

Page 1

Single Mass Analysis

Tolerance = 5.0 mDa / DBE: min = -1.5, max = 100.0
Element prediction: Off
Number of isotope peaks used for i-FIT = 3

Monoisotopic Mass, Even Electron Ions
41 formula(e) evaluated with 1 results within limits (up to 50 closest results for each mass)
Elements Used:
C: 0-100 H: 0-250 N: 7-7 O: 0-20 32S: 2-2 35Cl: 1-1
HBL-31AUG21-210 351 (5.954) AM2 (Ar,25000.0,0.00,0.00); ABS
TOF MS ES+

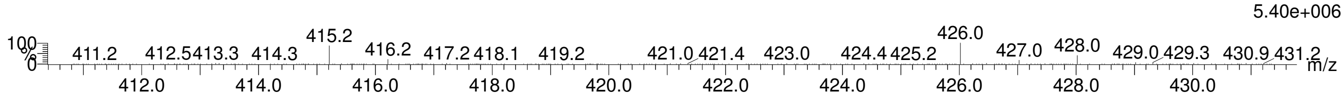

Mass	Calc. Mass	mDa	PPM	DBE	i-FIT	Norm	Conf(%)	Formula
Minimum:				-1.5				
Maximum:		5.0	5.0	100.0				
426.0212	426.0210	0.2	0.5	11.5	555.9	n/a	n/a	C14 H13 N7 O3 32S2 35Cl

Data File C:\Chem32\1\Data\HOBIN\HBL08313.D
Sample Name: HBL210

HPLC

===

Acq. Operator : PAOLA Seq. Line : 3
Acq. Instrument : Instrument 1 Location : 16
Injection Date : 8/31/2021 1:09:43 PM Inj : 1
Inj Volume : 100.000 µl
Different Inj Volume from Sample Entry! Actual Inj Volume : 8.000 µl
Acq. Method : C:\HPCHEM\1\METHODS\HBLA5T95.M
Last changed : 8/27/2021 9:11:28 AM by PAOLA
Analysis Method : C:\Chem32\1\Methods\HBL_A05 95_B95 05_20MIN.M
Last changed : 9/15/2021 2:37:26 PM by SYSTEM
Additional Info : Peak(s) manually integrated

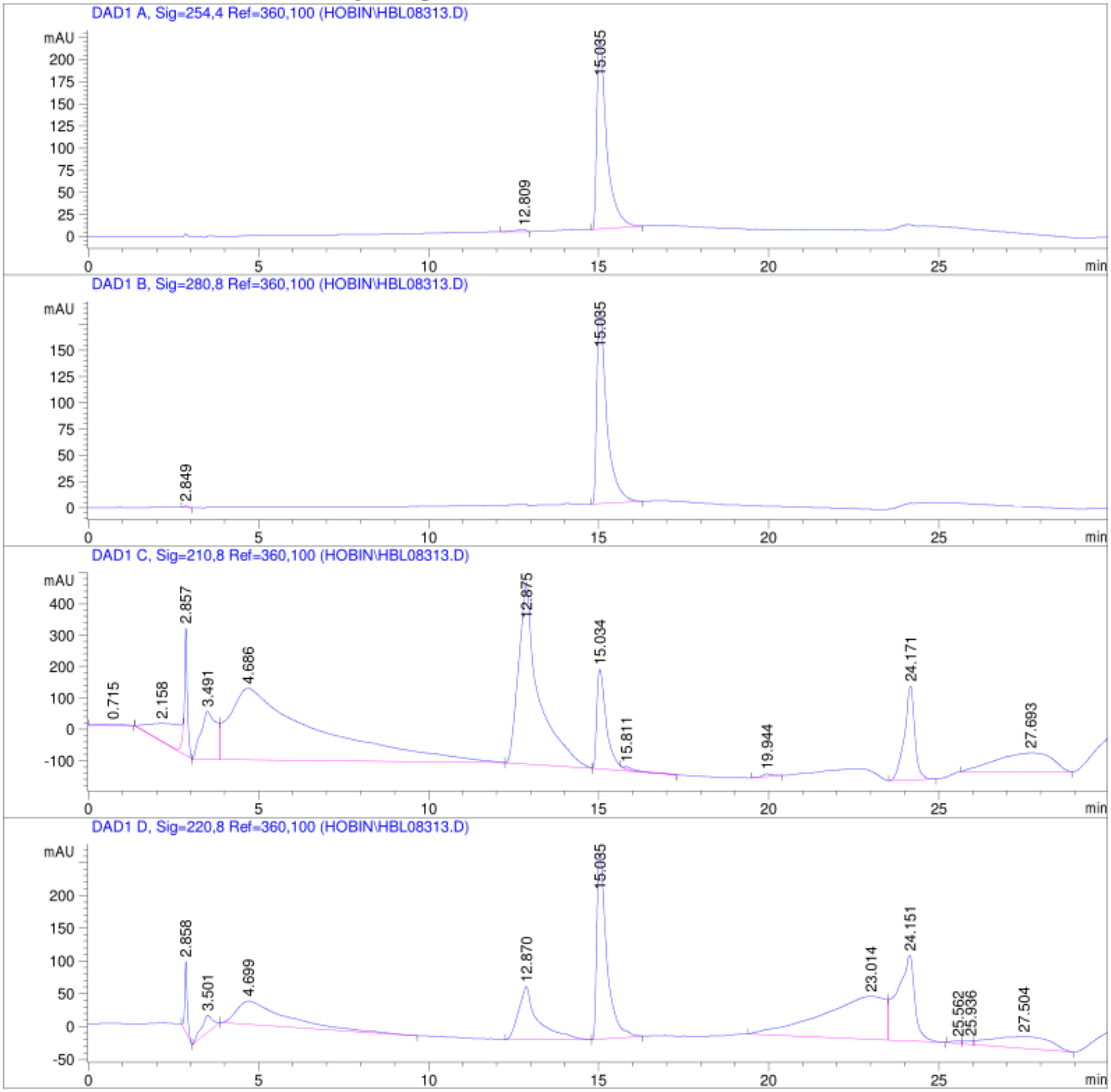

===
Area Percent Report
===

Sorted By : Signal
Multiplier : 1.0000
Dilution : 1.0000
Use Multiplier & Dilution Factor with ISTDs

Signal 1: DAD1 A, Sig=254,4 Ref=360,100

Peak #	RetTime [min]	Type	Width [min]	Area [mAU*s]	Height [mAU]	Area %
1	12.809	BB	0.3219	57.19051	2.31764	1.2787
2	15.035	BB	0.3071	4415.19434	213.64574	98.7213

Totals : 4472.38485 215.96338

Signal 2: DAD1 B, Sig=280,8 Ref=360,100

Peak #	RetTime [min]	Type	Width [min]	Area [mAU*s]	Height [mAU]	Area %
1	2.849	BB	0.0817	10.74781	1.85240	0.2829
2	15.035	BB	0.3069	3788.15430	183.45390	99.7171

Totals : 3798.90211 185.30630

Signal 3: DAD1 C, Sig=210,8 Ref=360,100

Peak #	RetTime [min]	Type	Width [min]	Area [mAU*s]	Height [mAU]	Area %
1	0.715	BB	0.5726	191.21915	4.45648	0.1998
2	2.158	BV E	0.8782	3966.02539	58.11294	4.1432
3	2.857	VB R	0.0874	2559.21826	406.64340	2.6736
4	3.491	BV	0.4245	4923.69482	154.21886	5.1437
5	4.686	VB	2.2680	4.03724e4	228.42746	42.1764
6	12.875	BB	0.5659	2.36588e4	579.98438	24.7159
7	15.034	BV R	0.2972	6285.57813	317.16611	6.5664
8	15.811	VB E	0.3504	218.13921	8.30609	0.2279
9	19.944	BB	0.2932	171.49976	8.58092	0.1792
10	24.171	BV R	0.3037	6382.65381	300.58713	6.6678
11	27.693	BB	1.3901	6993.57910	60.25454	7.3061

Totals : 9.57228e4 2126.73830

Compound 16

^{1}H NMR spectrum

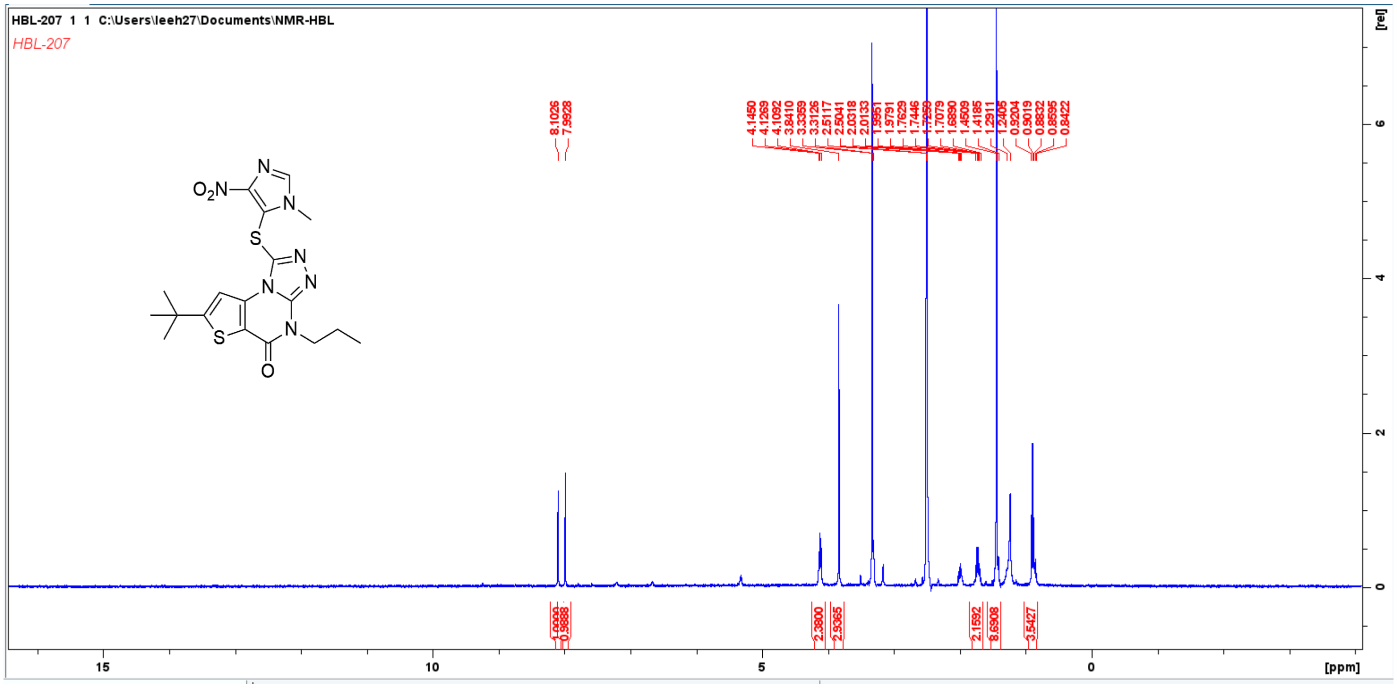

Mass spectrum

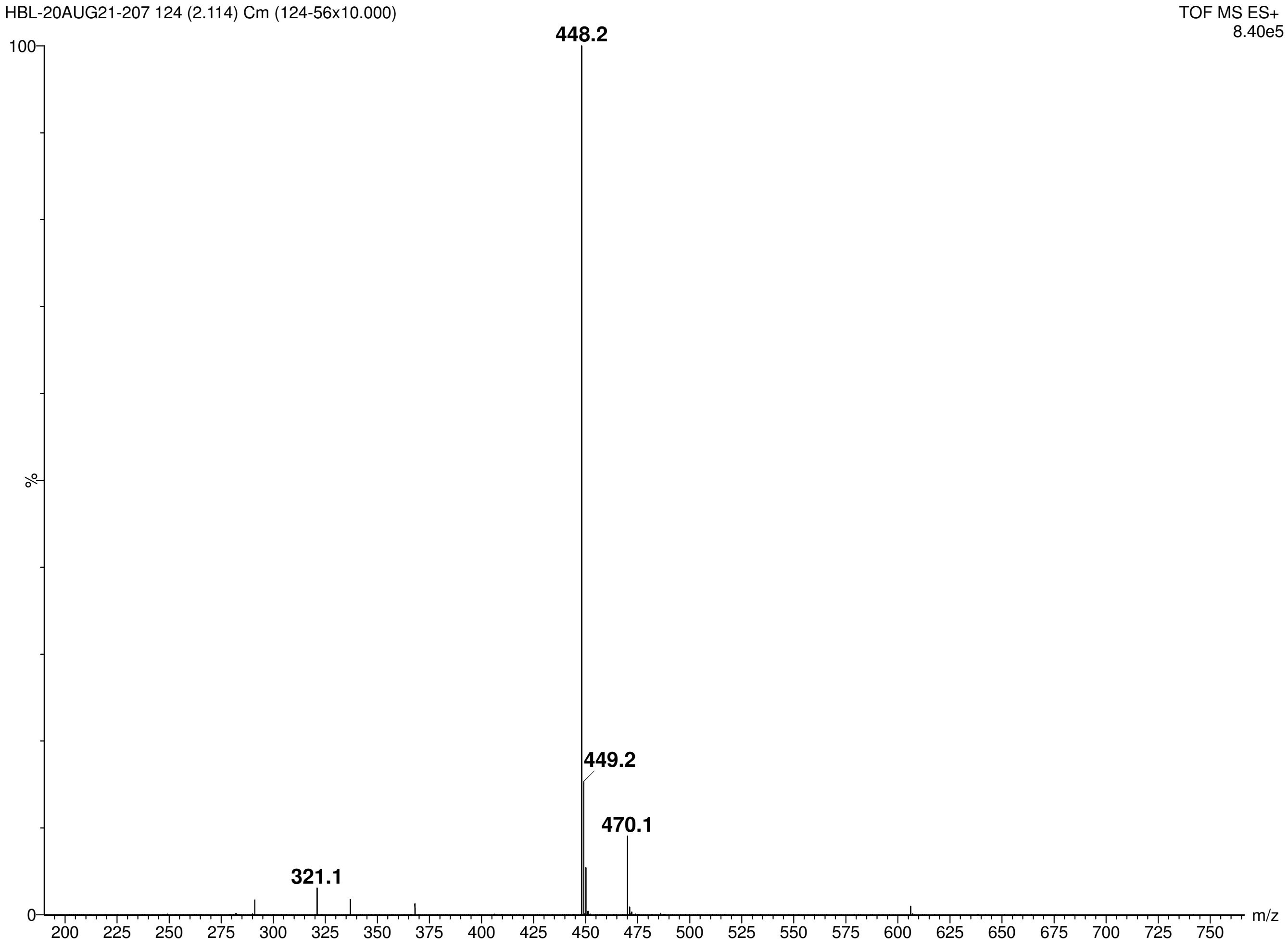

Elemental Composition Report

Page 1

Single Mass Analysis

Tolerance = 5.0 mDa / DBE: min = -1.5, max = 100.0
Element prediction: Off
Number of isotope peaks used for i-FIT = 3

Monoisotopic Mass, Even Electron Ions
57 formula(e) evaluated with 1 results within limits (up to 50 closest results for each mass)
Elements Used:
C: 0-100 H: 0-250 N: 7-7 O: 0-20 32S: 2-2
HBL-20AUG21-207 127 (2.165) AM2 (Ar,25000.0,0.00,0.00); ABS
TOF MS ES+

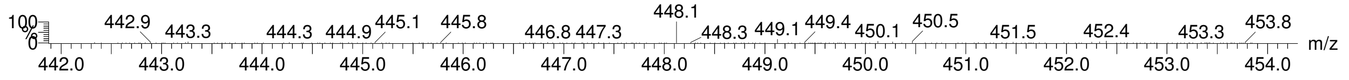

Mass	Calc. Mass	mDa	PPM	DBE	i-FIT	Norm	Conf(%)	Formula
Minimum:				-1.5				
Maximum:		5.0	5.0	100.0				
448.1221	448.1226	-0.5	-1.1	11.5	466.9	n/a	n/a	C18 H22 N7 O3 32S2

Data File C:\Chem32\1\Data\HOBIN\HBL08272.D
Sample Name: HBL207

HPLC

===

Acq. Operator : PAOLA Seq. Line : 2
Acq. Instrument : Instrument 1 Location : 12
Injection Date : 8/27/2021 9:55:41 AM Inj : 1
Inj Volume : 100.000 µl
Different Inj Volume from Sample Entry! Actual Inj Volume : 8.000 µl
Acq. Method : C:\HPCHEM\1\METHODS\HBLA5T95.M
Last changed : 8/27/2021 9:11:28 AM by PAOLA
Analysis Method : C:\Chem32\1\Methods\HBL_A05 95_B95 05_20MIN.M
Last changed : 9/15/2021 2:37:26 PM by SYSTEM
Additional Info : Peak(s) manually integrated

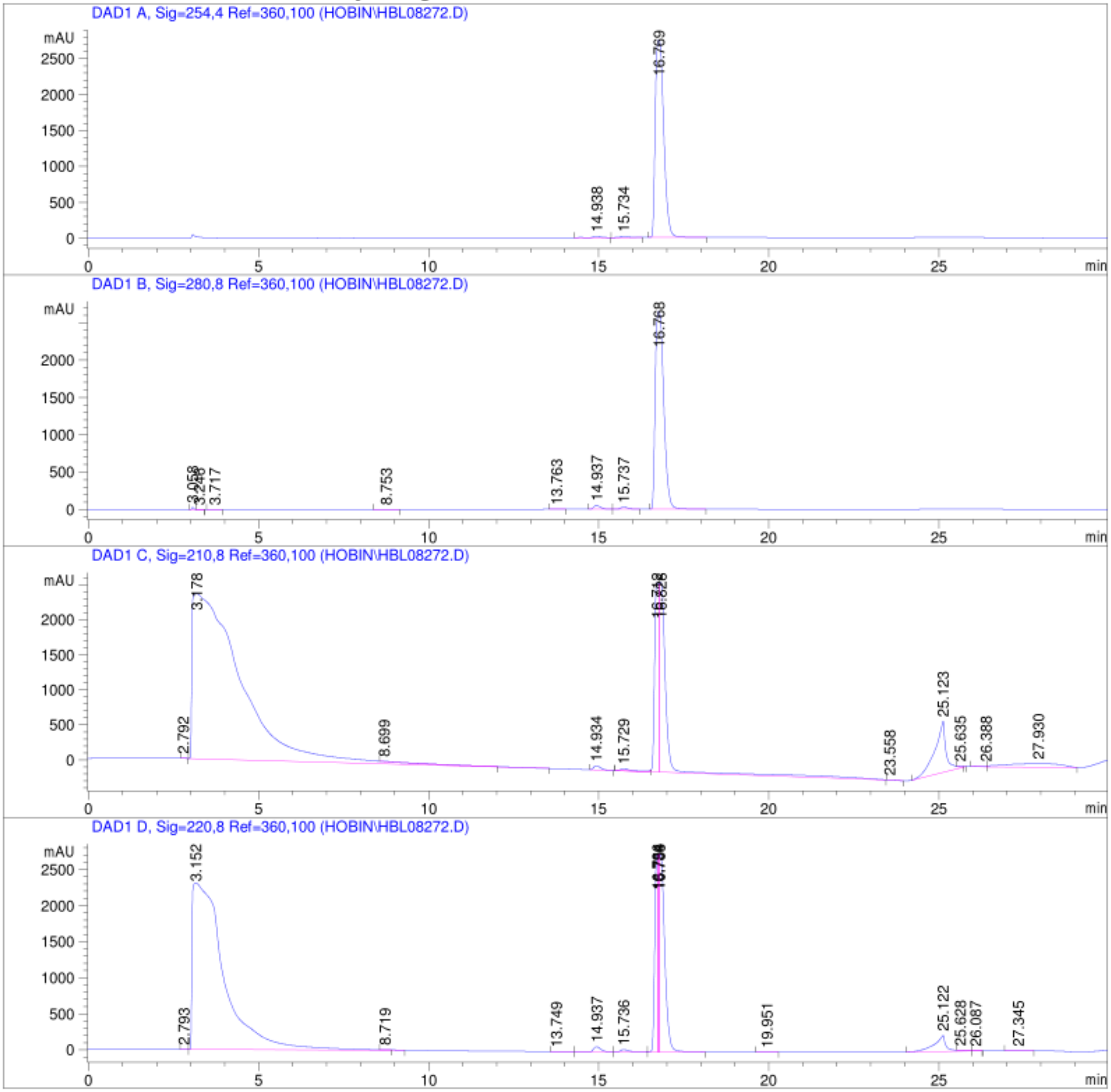

===

Area Percent Report

===

Sorted By : Signal
Multiplier : 1.0000
Dilution : 1.0000
Use Multiplier & Dilution Factor with ISTDs

Signal 1: DAD1 A, Sig=254,4 Ref=360,100

Peak #	RetTime [min]	Type	Width [min]	Area [mAU*s]	Height [mAU]	Area %
1	14.938	VB R	0.2260	308.77090	19.40110	0.6463
2	15.734	BB	0.2519	277.85129	17.07187	0.5816
3	16.769	BB	0.2740	4.71853e4	2752.75732	98.7720

Totals : 4.77719e4 2789.23030

Signal 2: DAD1 B, Sig=280,8 Ref=360,100

Peak #	RetTime [min]	Type	Width [min]	Area [mAU*s]	Height [mAU]	Area %
1	3.058	BV R	0.0617	114.34147	27.98954	0.2438
2	3.246	VB E	0.1164	8.01647	1.05740	0.0171
3	3.717	BB	0.1480	14.43710	1.40415	0.0308
4	8.753	BB	0.2108	16.07827	1.21142	0.0343
5	13.763	BB	0.2229	17.23404	1.24984	0.0368
6	14.937	BB	0.2252	699.33118	49.38911	1.4914
7	15.737	BB	0.2438	426.56326	27.36977	0.9097
8	16.768	BB	0.2747	4.55957e4	2650.28735	97.2362

Totals : 4.68917e4 2759.95858

Signal 3: DAD1 C, Sig=210,8 Ref=360,100

Peak #	RetTime [min]	Type	Width [min]	Area [mAU*s]	Height [mAU]	Area %
1	2.792	BB	0.1148	30.42336	4.28614	9.542e 3
2	3.178	BV R	1.2178	2.33868e5	2356.52612	73.3513
3	8.699	VB E	0.5720	194.96768	4.37409	0.0612
4	14.934	BB	0.2239	799.38049	56.89571	0.2507
5	15.729	BV E	0.2947	607.19171	30.18139	0.1904
6	16.712	VV R	0.1236	2.34726e4	2695.97144	7.3620
7	16.828	VB	0.1949	3.73371e4	2707.04395	11.7106
8	23.558	BB	0.2741	59.36706	3.11822	0.0186
9	25.123	BV R	0.2857	1.67310e4	729.15057	5.2476
10	25.635	VV E	0.0950	81.71651	13.05194	0.0256
11	26.388	BV	0.2263	210.09898	11.76969	0.0659

Compound 18

^{1}H NMR spectrum

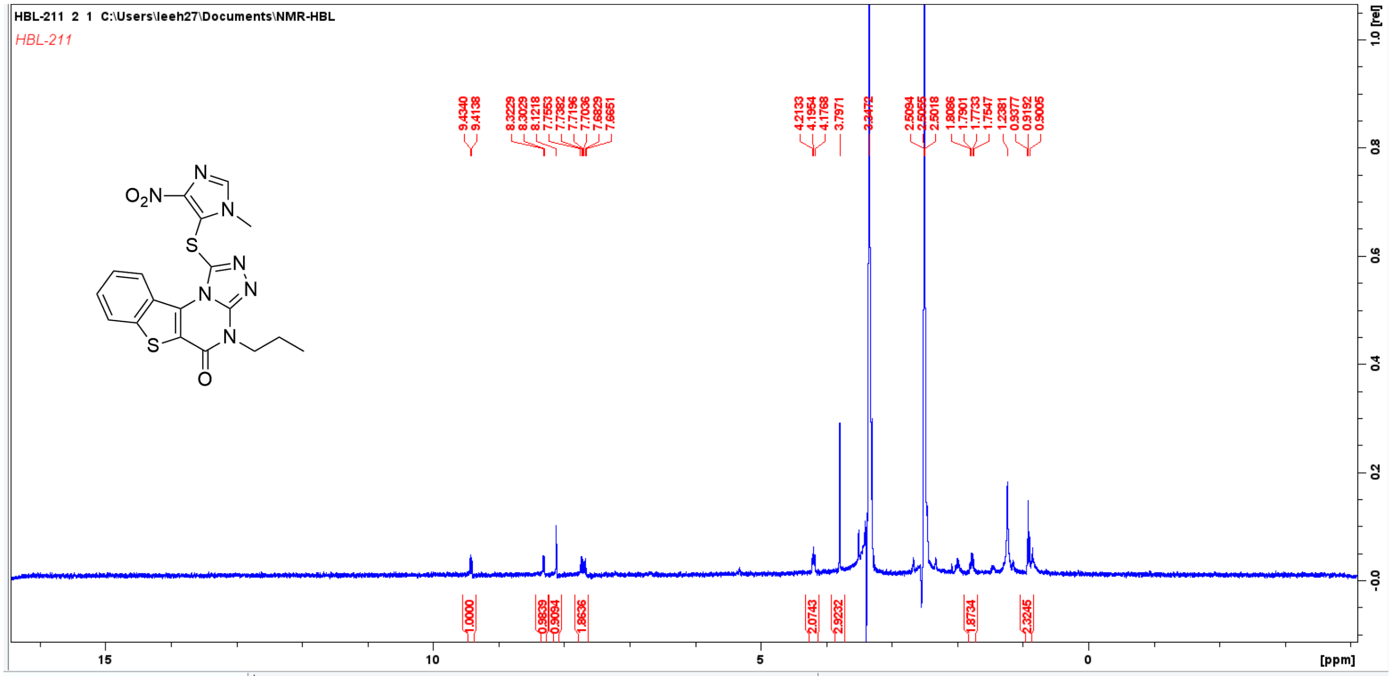

Mass spectrum

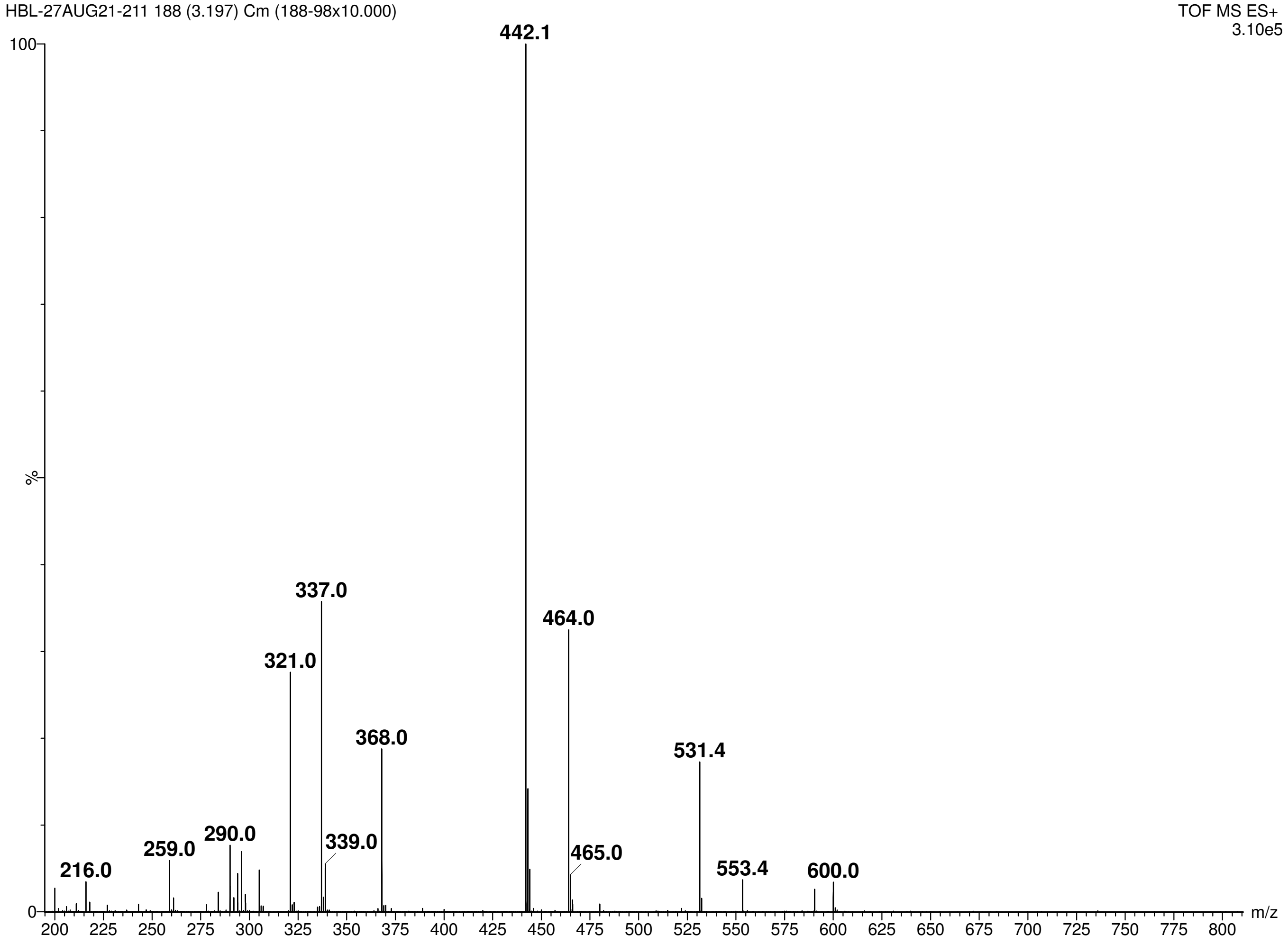

Elemental Composition Report

Page 1

Single Mass Analysis

Tolerance = 5.0 mDa / DBE: min = -1.5, max = 100.0
Element prediction: Off
Number of isotope peaks used for i-FIT = 3

Monoisotopic Mass, Even Electron Ions
58 formula(e) evaluated with 1 results within limits (up to 50 closest results for each mass)
Elements Used:
C: 0-100 H: 0-250 N: 7-7 O: 0-20 32S: 2-2
HBL-27AUG21-211 187 (3.180) AM2 (Ar,25000.0,0.00,0.00); ABS
TOF MS ES+

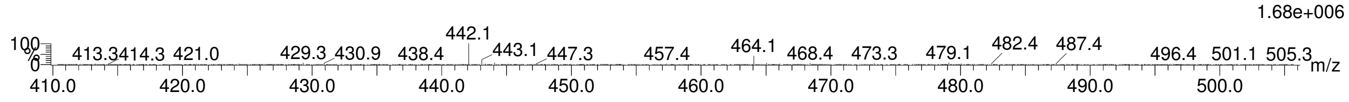

Mass	Calc. Mass	mDa	PPM	DBE	i-FIT	Norm	Conf(%)	Formula
Minimum:				-1.5				
Maximum:		5.0	5.0	100.0				
442.0762	442.0756	0.6	1.4	14.5	384.9	n/a	n/a	C18 H16 N7 O3 32S2

Data File C:\Chem32\1\Data\HOBIN\HBL08275.D
Sample Name: HBL211

HPLC

===

Acq. Operator : PACLA   Seq. Line : 5
Acq. Instrument : Instrument 1   Location : 15
Injection Date : 8/27/2021 11:29:10 AM   Inj : 1
Inj Volume : 100.000 µl
Different Inj Volume from Sample Entry! Actual Inj Volume : 8.000 µl
Acq. Method : C:\HPCHEM\1\METHODS\HBLA5T95.M
Last changed : 8/27/2021 9:11:28 AM by PACLA
Analysis Method : C:\Chem32\1\Methods\HBL_A05-95_B95-05_20MIN.M
Last changed : 9/15/2021 2:37:26 PM by SYSTEM
Additional Info : Peak(s) manually integrated

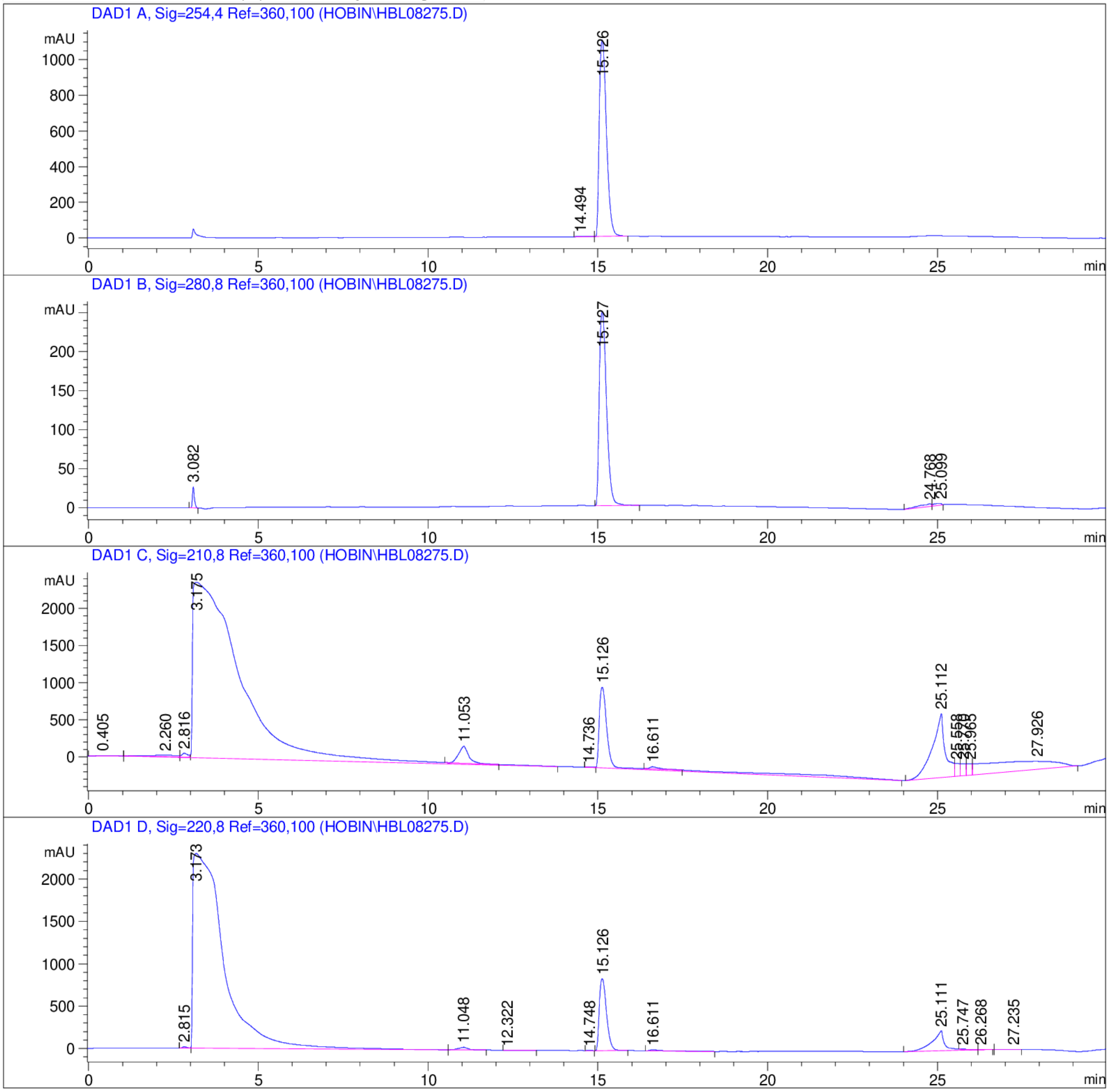

===

Area Percent Report

===

Sorted By : Signal
Multiplier : 1.0000
Dilution : 1.0000
Use Multiplier & Dilution Factor with ISTDs

Signal 1: DAD1 A, Sig=254,4 Ref=360,100

Peak #	RetTime [min]	Type	Width [min]	Area [mAU*s]	Height [mAU]	Area %
1	14.494	BB	0.2595	26.13659	1.60911	0.1618
2	15.126	BB	0.2327	1.61246e4	1102.23242	99.8382

Totals : 1.61508e4 1103.84153

Signal 2: DAD1 B, Sig=280,8 Ref=360,100

Peak #	RetTime [min]	Type	Width [min]	Area [mAU*s]	Height [mAU]	Area %
1	3.082	BB	0.0597	105.27458	26.85098	2.7050
2	15.127	BB	0.2339	3666.10352	248.88199	94.1983
3	24.768	BV	0.3727	84.11865	2.84737	2.1614
4	25.099	VB	0.2546	36.40422	1.75086	0.9354

Totals : 3891.90097 280.33119

Signal 3: DAD1 C, Sig=210,8 Ref=360,100

Peak #	RetTime [min]	Type	Width [min]	Area [mAU*s]	Height [mAU]	Area %
1	0.405	BB	0.4634	137.85814	3.69360	0.0447
2	2.260	BV E	0.7923	1361.90845	23.28656	0.4417
3	2.816	VV E	0.1827	690.56372	54.17210	0.2240
4	3.175	VV R	1.1739	2.28747e5	2362.00146	74.1891
5	11.053	VB E	0.3188	4783.74023	231.78017	1.5515
6	14.736	BV E	0.1952	63.33775	4.82299	0.0205
7	15.126	VV R	0.3249	2.45177e4	1087.82861	7.9518
8	16.611	VB E	0.2887	572.63269	28.46863	0.1857
9	25.112	BV	0.3298	2.30193e4	861.19098	7.4658
10	25.558	VV	0.1329	1641.80273	175.83464	0.5325
11	25.770	VV	0.1600	1771.73816	161.54063	0.5746
12	25.965	VV	0.1444	1645.36475	151.83604	0.5336
13	27.926	VB	2.0942	1.93764e4	108.98524	6.2843

Totals : 3.08329e5 5255.44167

Compound 19 (Allopole-A)

^{1}H NMR spectrum

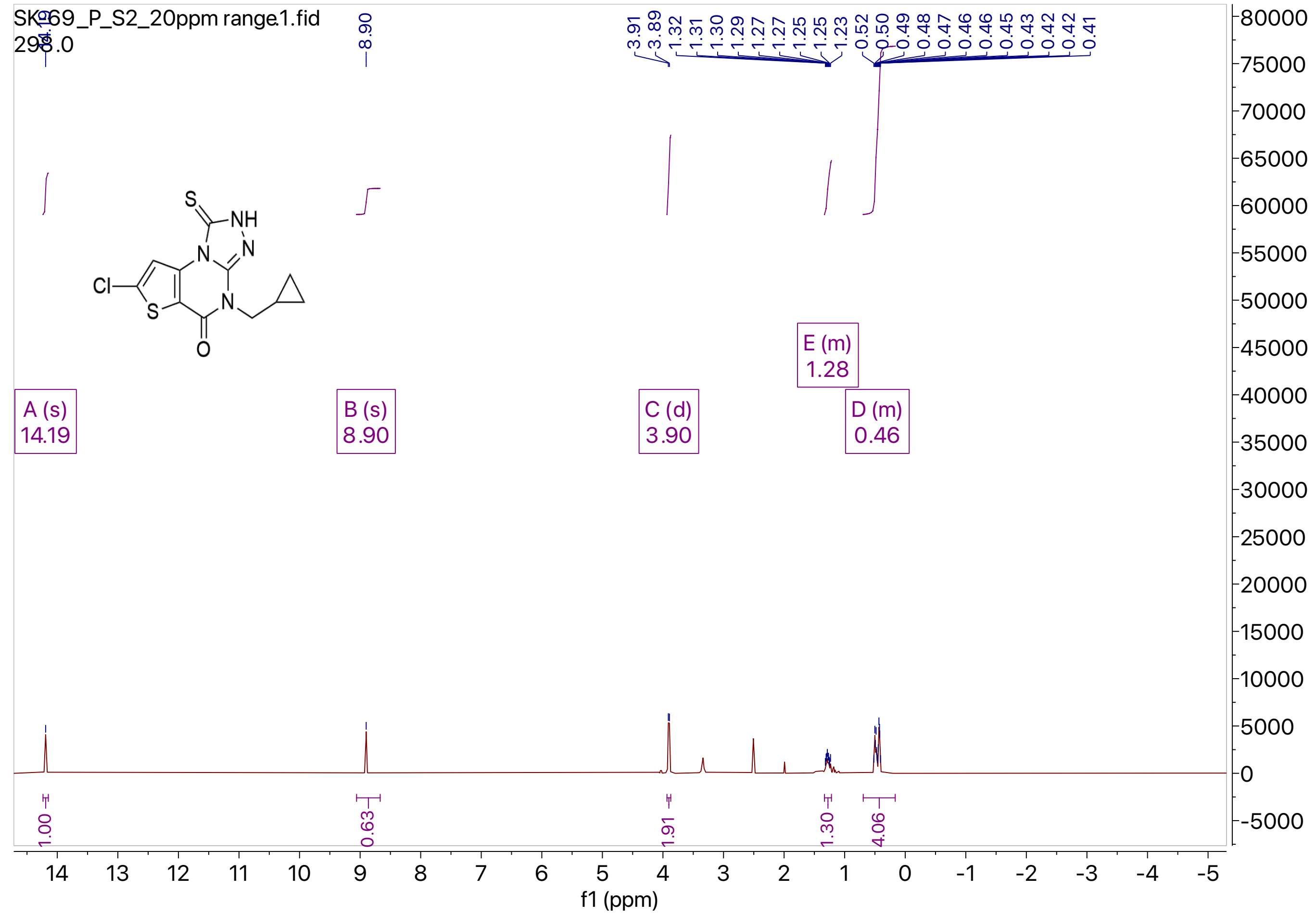

^{13}C NMR spectrum (Allopole-A)

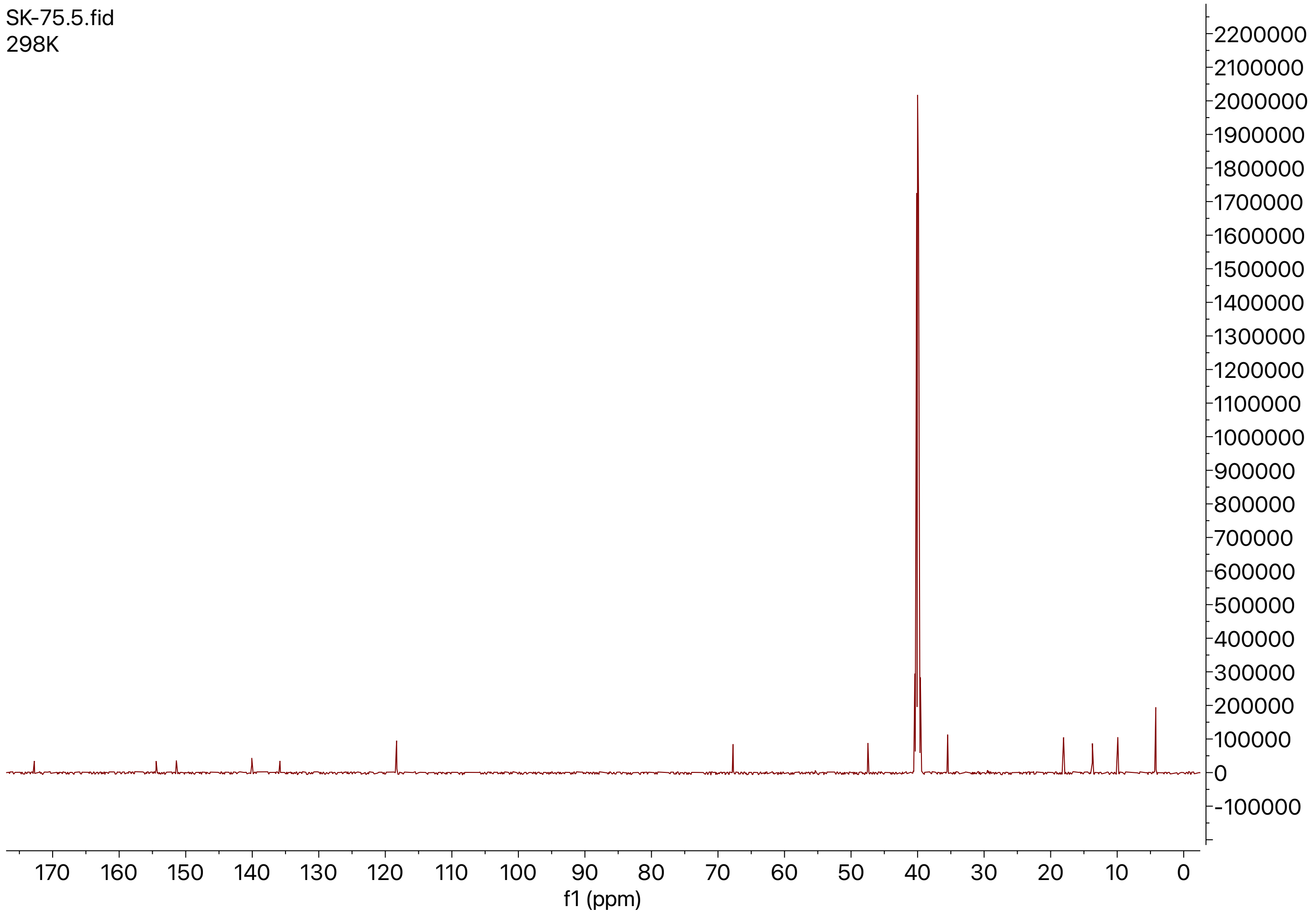

Mass spectrum (Allopole-A)

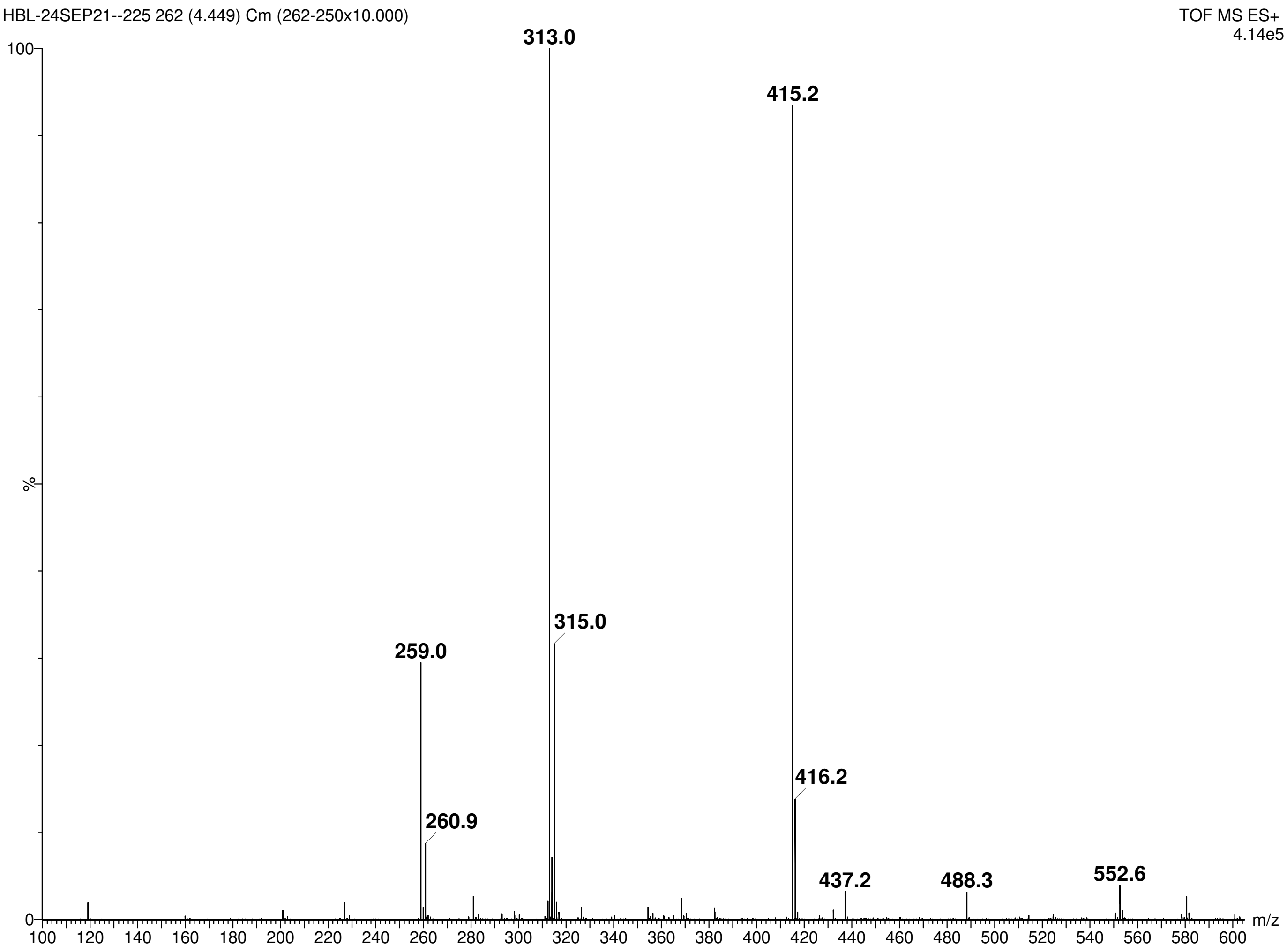

Elemental Composition Report

Single Mass Analysis

Tolerance = 5.0 mDa / DBE: min = -1.5, max = 100.0
Element prediction: Off
Number of isotope peaks used for i-FIT = 3

Monoisotopic Mass, Even Electron Ions
21 formula(e) evaluated with 1 results within limits (up to 50 closest results for each mass)
Elements Used:
C: 0-100 H: 0-250 N: 4-4 O: 0-20 32S: 2-2 35Cl: 1-1
HBL-24SEP21--225 259 (4.398) AM2 (Ar,25000.0,0.00,0.00); ABS
TOF MS ES+

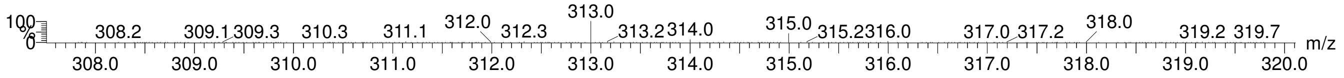

Mass	Calc. Mass	mDa	PPM	DBE	i-FIT	Norm	Conf(%)	Formula
Minimum:				-1.5				
Maximum:		5.0	3.0	100.0				
312.9982	312.9985	-0.3	-1.0	8.5	596.9	n/a	n/a	C11 H10 N4 O 32S2 35Cl

Data File C:\Chem32\1\Data\HOBIN\Hobin 2021-09-24 11-15-23\HBL2240000002.D
Sample Name: HBL225

HPLC (Allopole-A)

===

Acq. Operator : SYSTEM Seq. Line : 2
Acq. Instrument : hplc Location : 3
Injection Date : 9/24/2021 11:48:10 AM Inj : 1
Inj Volume : 100.000 µl
Different Inj Volume from Sample Entry! Actual Inj Volume : 50.000 µl
Method : C:\Chem32\1\Data\HOBIN\Hobin 2021-09-24 11-15-23\HBL_A05-95_B95-05_20MIN.M
(Sequence Method)
Last changed : 9/24/2021 11:15:23 AM by SYSTEM
Additional Info : Peak(s) manually integrated

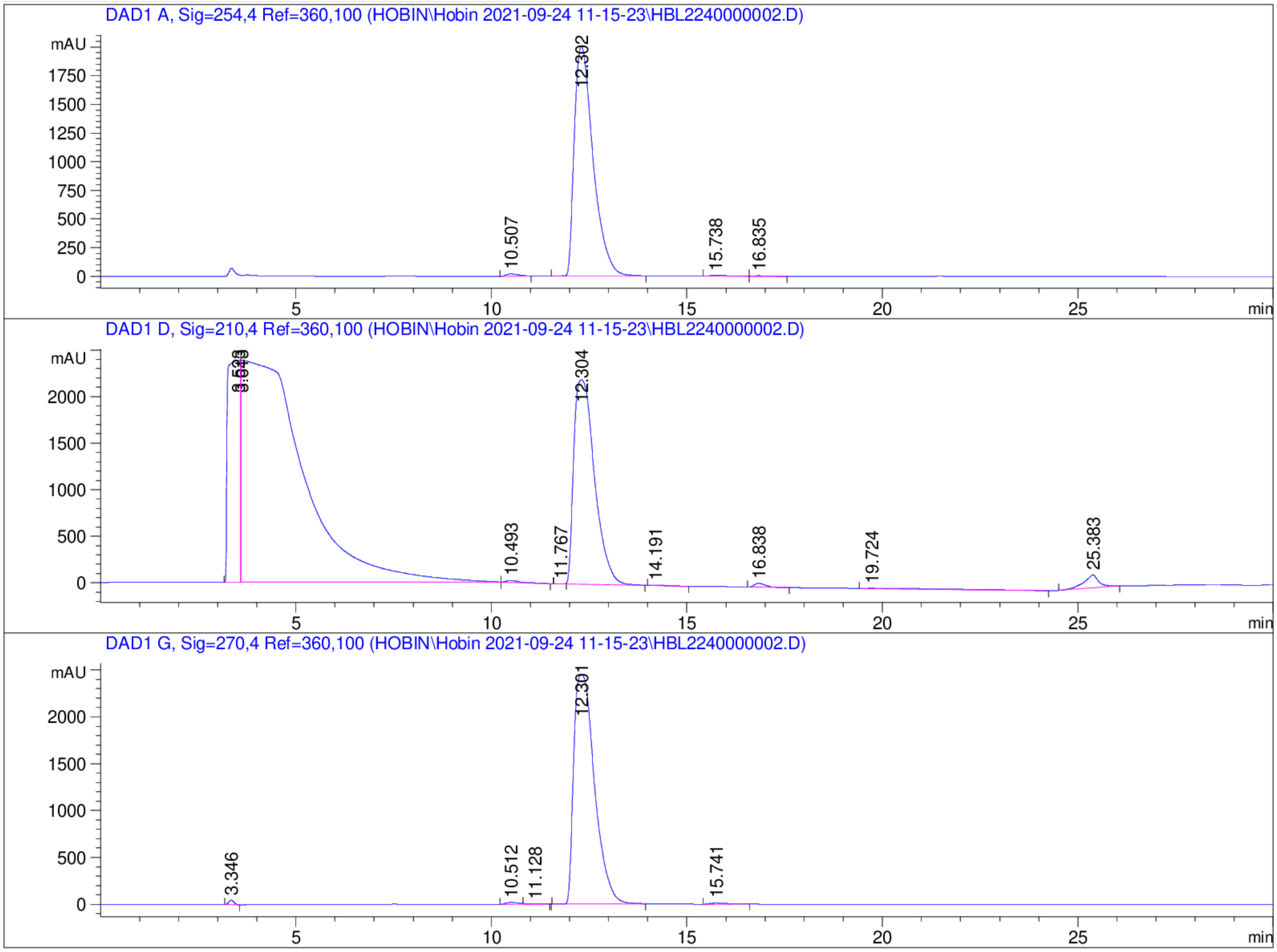

===
Fraction Information
===
Fraction collection using a timetable
===
No Fractions found.
===

===

Area Percent Report

===

Sorted By : Signal
Multiplier : 1.0000
Dilution : 1.0000
Use Multiplier & Dilution Factor with ISTDs

Signal 1: DAD1 A, Sig=254,4 Ref=360,100

Peak #	RetTime [min]	Type	Width [min]	Area [mAU*s]	Height [mAU]	Area %
1	10.507	BB	0.3181	391.36029	19.34163	0.5791
2	12.302	BB	0.5126	6.68004e4	2005.55029	98.8400
3	15.738	BV	0.4536	278.30396	8.78448	0.4118
4	16.835	VB	0.3245	114.32709	5.16062	0.1692

Totals : 6.75844e4 2038.83703

Signal 2: DAD1 D, Sig=210,4 Ref=360,100

Peak #	RetTime [min]	Type	Width [min]	Area [mAU*s]	Height [mAU]	Area %
1	3.523	BV	0.2604	4.99423e4	2383.86060	12.4470
2	3.613	VB	1.3073	2.64580e5	2383.00073	65.9404
3	10.493	BB	0.3474	441.10526	19.39086	0.1099
4	11.767	BV E	0.1855	28.42729	2.34338	7.085e-3
5	12.304	VB R	0.5718	8.08422e4	2194.57178	20.1480
6	14.191	BB	0.4168	90.24124	3.12588	0.0225
7	16.838	BB	0.2997	820.80725	42.05643	0.2046
8	19.724	BB	3.1138	773.59241	2.92291	0.1928
9	25.383	BB	0.3567	3722.21118	139.75487	0.9277

Totals : 4.01241e5 7171.02743

Signal 3: DAD1 G, Sig=270,4 Ref=360,100

Peak #	RetTime [min]	Type	Width [min]	Area [mAU*s]	Height [mAU]	Area %
1	3.346	BB	0.1364	416.45032	47.45233	0.4565
2	10.512	BV R	0.3634	481.81393	19.97523	0.5281
3	11.128	VB E	0.3131	47.34087	2.38975	0.0519
4	12.301	BB	0.5689	8.99455e4	2446.69189	98.5906
5	15.741	BB	0.4188	340.21893	12.07355	0.3729

Totals : 9.12314e4 2528.58276

Compound 20 (Allopole)

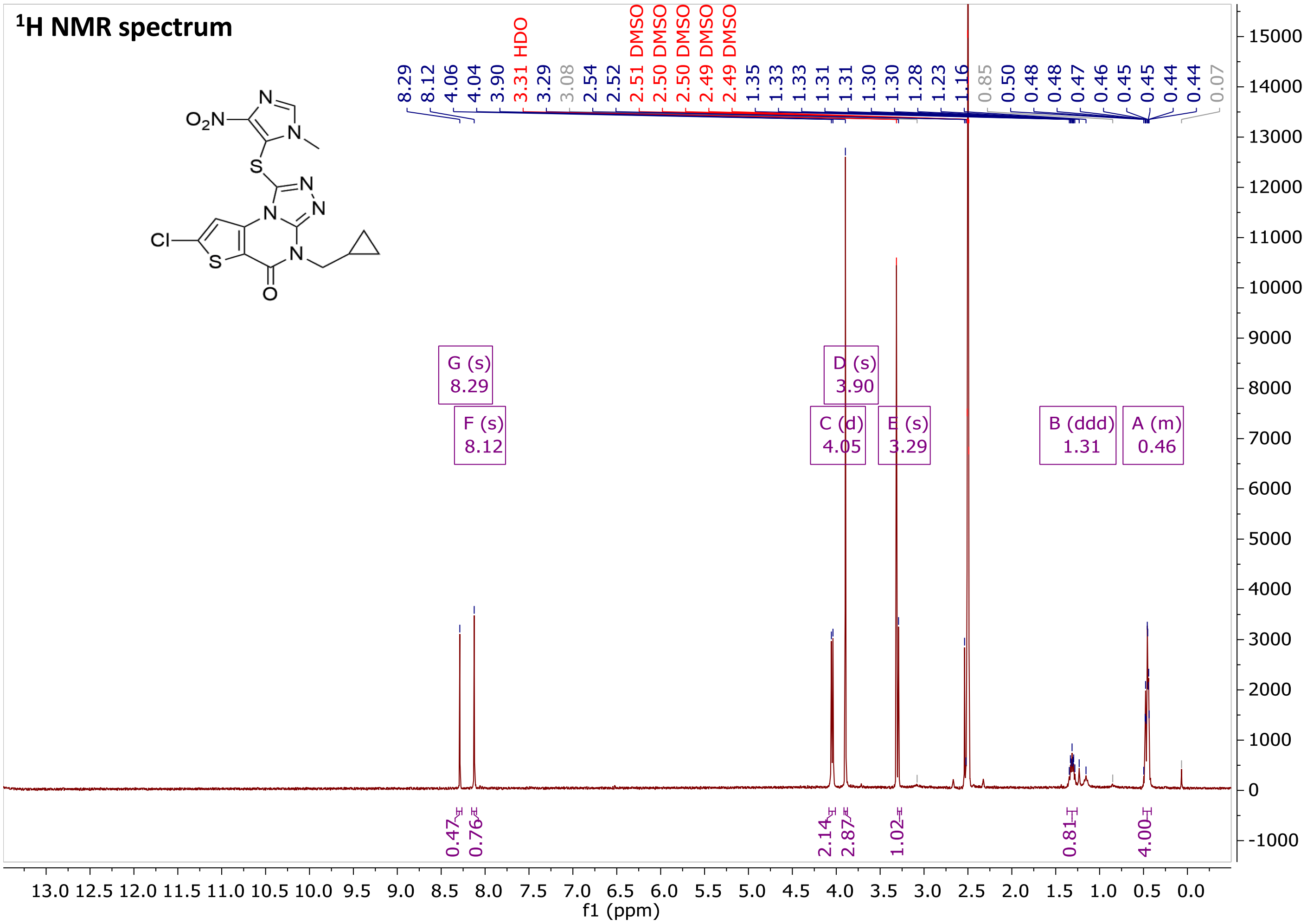

^{13}C NMR spectrum (Allopole)

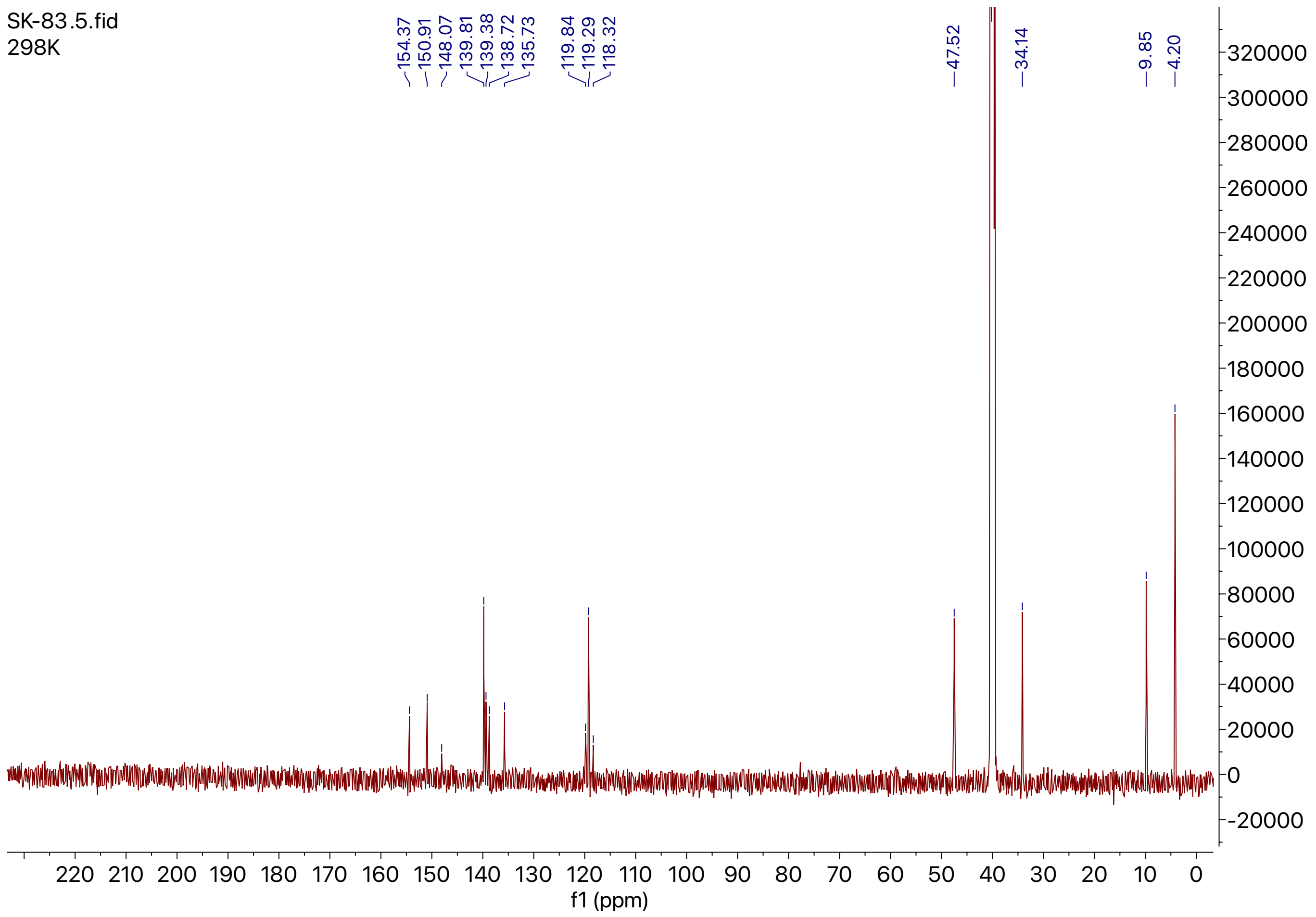

Mass spectrum (Allopole)

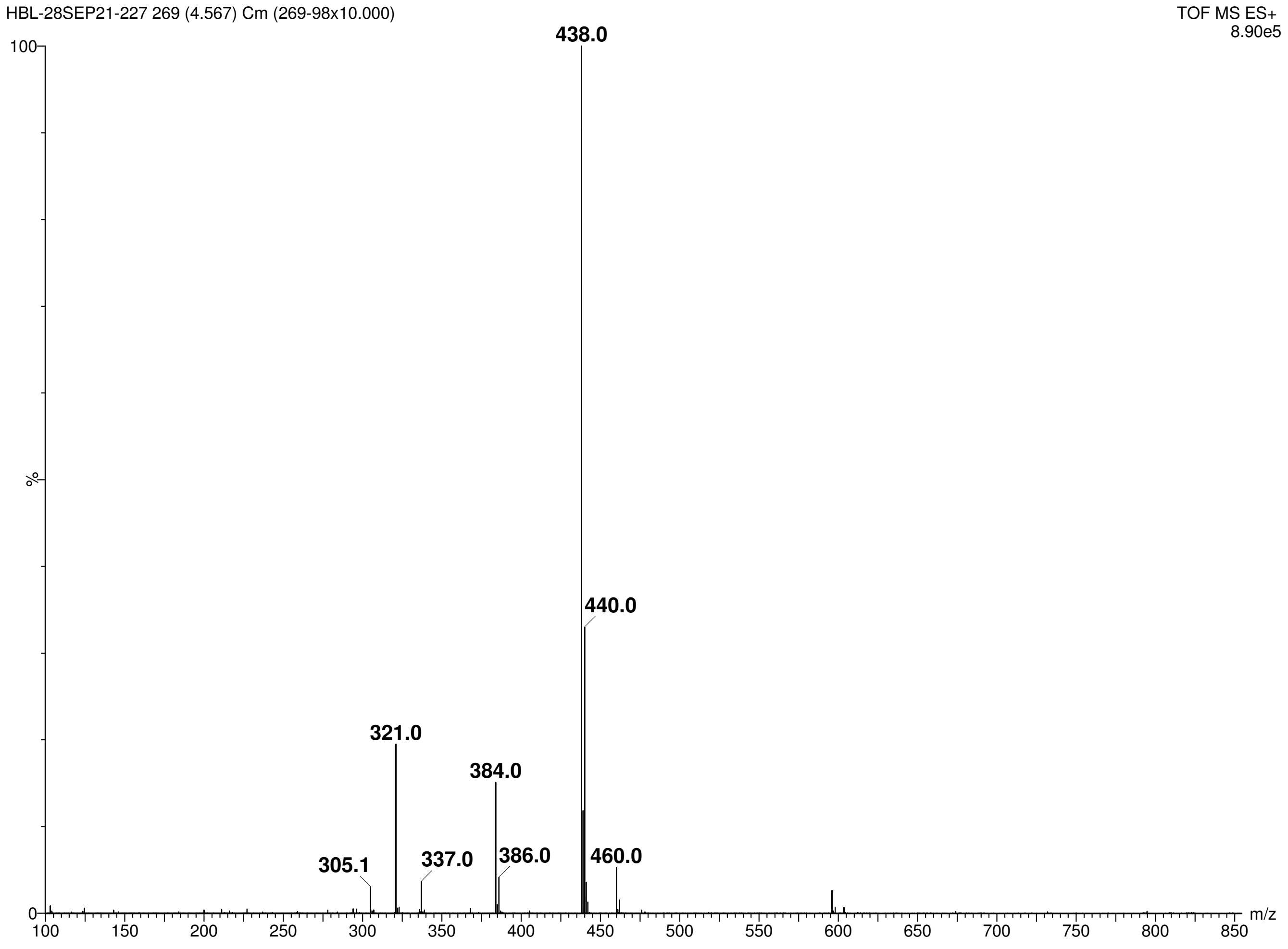

Elemental Composition Report

Page 1

Single Mass Analysis

Tolerance = 5.0 mDa / DBE: min = -1.5, max = 100.0
Element prediction: Off
Number of isotope peaks used for i-FIT = 3

Monoisotopic Mass, Even Electron Ions
45 formula(e) evaluated with 1 results within limits (up to 50 closest results for each mass)
Elements Used:
C: 0-100 H: 0-250 N: 7-7 O: 0-20 32S: 2-2 35Cl: 1-1
HBL-28SEP21-227 274 (4.652) AM2 (Ar,25000.0,0.00,0.00); ABS
TOF MS ES+

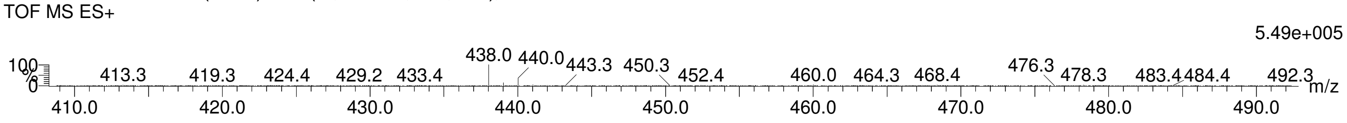

Minimum:				-1.5				
Maximum:		5.0	3.0	100.0				
Mass	Calc. Mass	mDa	PPM	DBE	i-FIT	Norm	Conf(%)	Formula
438.0217	438.0210	0.7	1.6	12.5	415.9	n/a	n/a	C15 H13 N7 O3 32S2 35Cl

Data File C:\Chem32\1\Data\HOBIN\HBL22701.D
Sample Name: HBL227

HPLC (Allopole)

===

Acq. Operator	: HOBIN	Seq. Line :	1
Acq. Instrument	: Instrument 1	Location :	12
Injection Date	: 9/28/2021 11:34:29 AM	Inj :	1
		Inj Volume :	100.000 µl

Different Inj Volume from Sample Entry! Actual Inj Volume : 10.000 µl
Acq. Method : C:\HPCHEM\1\METHODS\HBLA5T95.M
Last changed : 8/27/2021 9:11:28 AM by PACLA
Analysis Method : C:\Chem32\1\Methods\LBF_AN_A75_B25_20MIN.M
Last changed : 9/27/2021 3:23:42 PM by SYSTEM
Additional Info : Peak(s) manually integrated

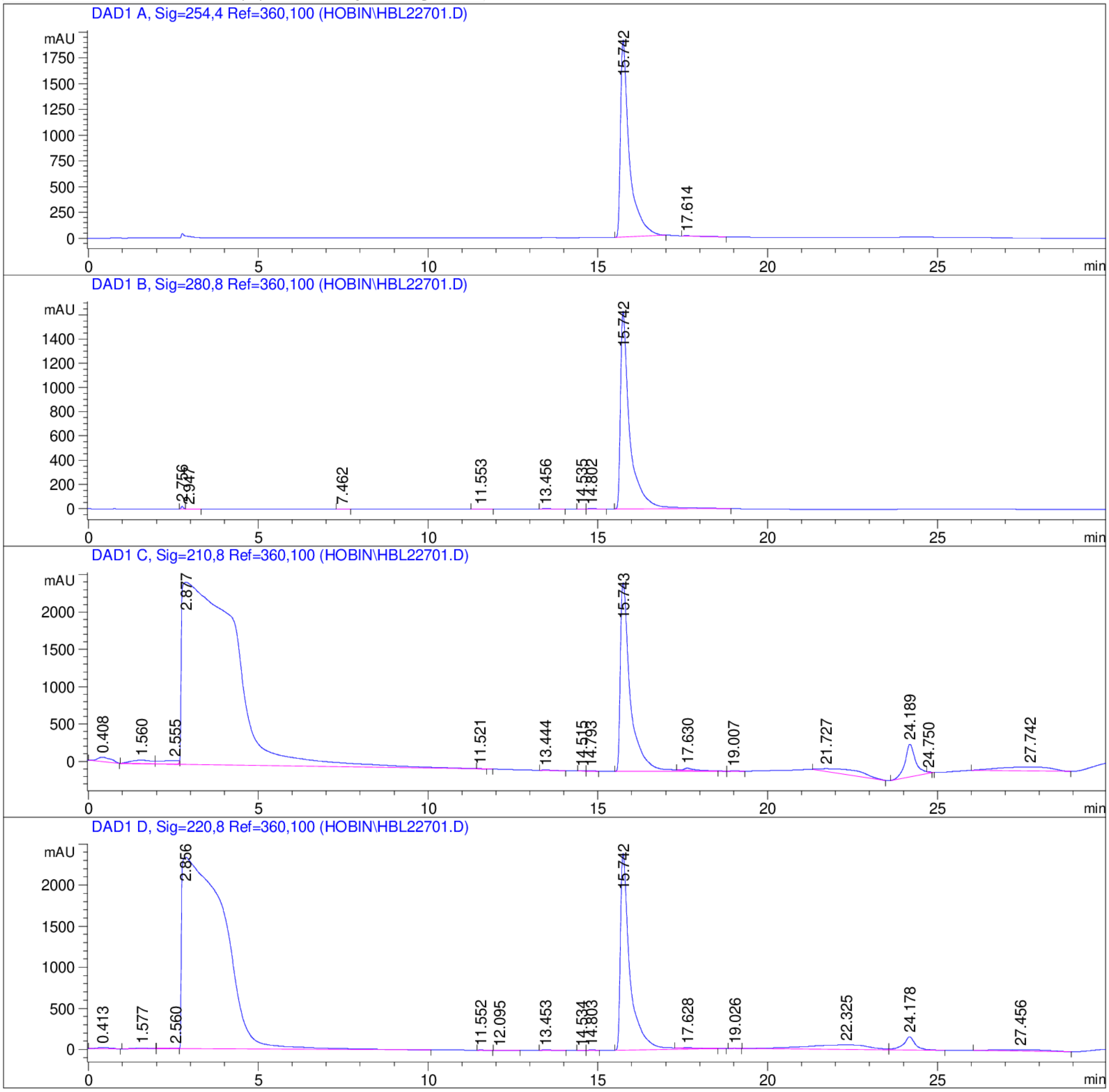

===

Area Percent Report

===

Sorted By : Signal
Multiplier : 1.0000
Dilution : 1.0000
Use Multiplier & Dilution Factor with ISTDs

Signal 1: DAD1 A, Sig=254,4 Ref=360,100

Peak #	RetTime [min]	Type	Width [min]	Area [mAU*s]	Height [mAU]	Area %
1	15.742	BB	0.2633	3.49486e4	1911.92249	99.8519
2	17.614	BB	0.2700	51.84555	2.82584	0.1481

Totals : 3.50005e4 1914.74832

Signal 2: DAD1 B, Sig=280,8 Ref=360,100

Peak #	RetTime [min]	Type	Width [min]	Area [mAU*s]	Height [mAU]	Area %
1	2.756	BV R	0.0605	87.16225	21.85631	0.2756
2	2.947	VB E	0.1590	14.12654	1.29799	0.0447
3	7.462	BB	0.1789	14.79594	1.31851	0.0468
4	11.553	BB	0.2218	25.71383	1.58085	0.0813
5	13.456	BB	0.2419	77.04787	4.83629	0.2436
6	14.535	BV	0.1495	12.76183	1.31234	0.0403
7	14.802	VB	0.2198	59.94395	4.06990	0.1895
8	15.742	BB	0.2737	3.13370e4	1634.47766	99.0782

Totals : 3.16285e4 1670.74986

Signal 3: DAD1 C, Sig=210,8 Ref=360,100

Peak #	RetTime [min]	Type	Width [min]	Area [mAU*s]	Height [mAU]	Area %
1	0.408	BB	0.3970	1674.22461	59.37255	0.4812
2	1.560	BV E	0.6680	1947.72388	45.26337	0.5598
3	2.555	VV E	0.4818	1795.09668	46.55812	0.5159
4	2.877	VV R	1.3596	2.69353e5	2436.81934	77.4168
5	11.521	VB E	0.1568	12.62747	1.19953	3.629e-3
6	13.444	BB	0.2334	128.80602	8.01682	0.0370
7	14.515	BV	0.1358	12.86808	1.50401	3.699e-3
8	14.793	VB	0.1755	51.97607	4.68232	0.0149
9	15.743	BV R	0.3001	5.19724e4	2525.65869	14.9378
10	17.630	VB E	0.3048	662.14319	30.31235	0.1903
11	19.007	BB	0.1835	35.88265	3.04360	0.0103
12	21.727	BB	1.7280	4923.54932	34.56213	1.4151

Compound 22

^{1}H NMR spectrum

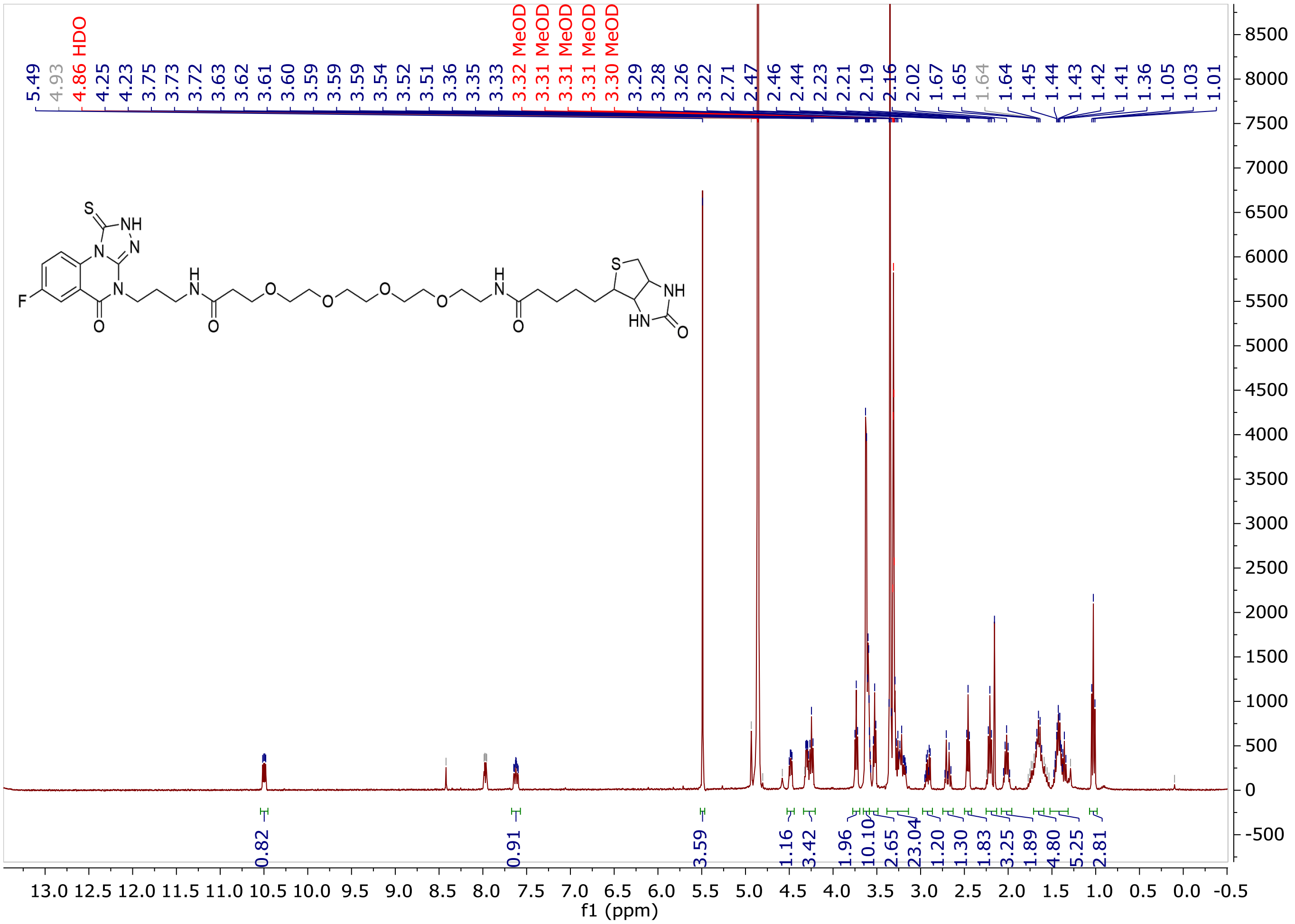

Mass spectrum

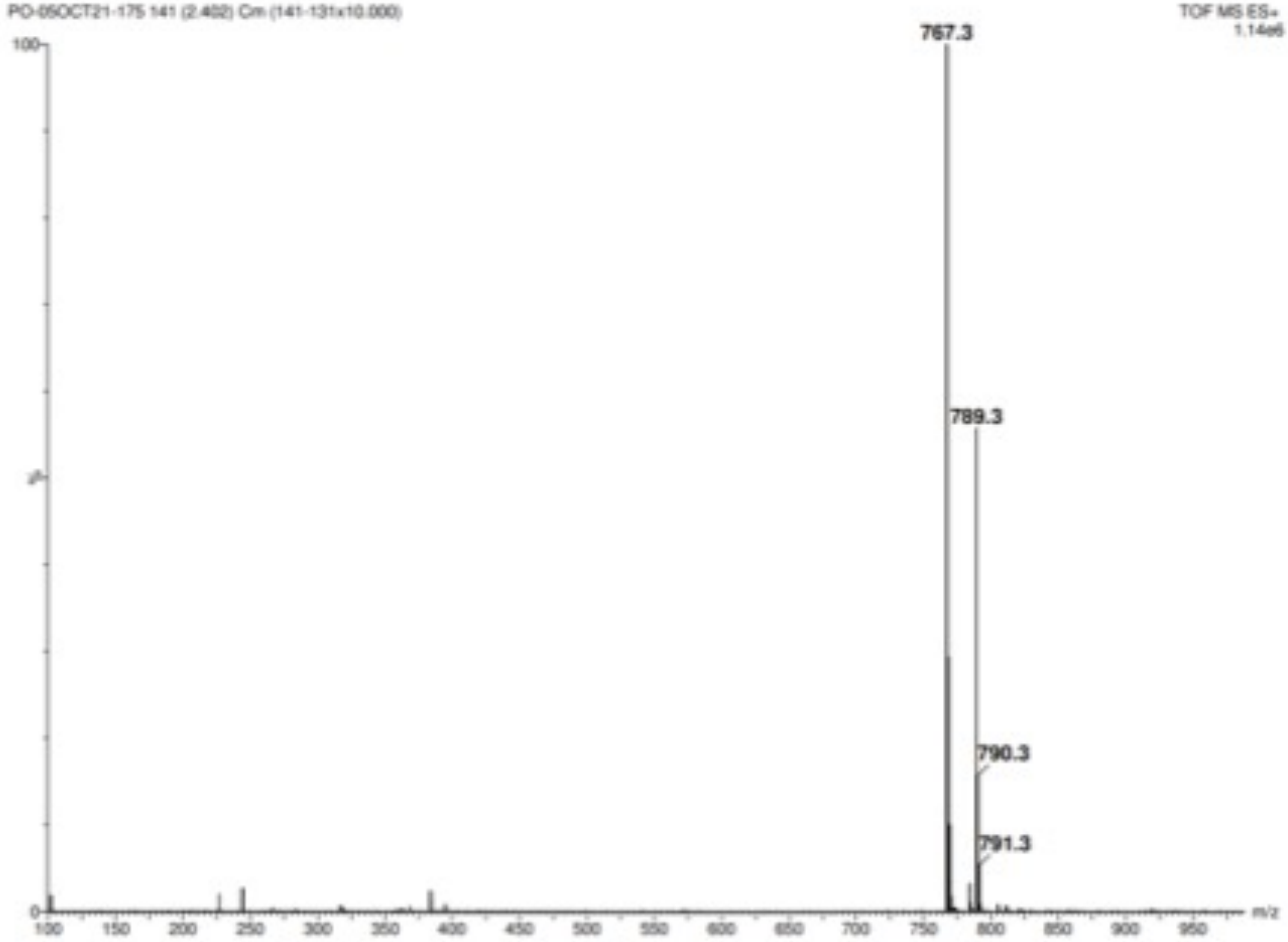

Elemental Composition Report

Page 1

Single Mass Analysis

Tolerance = 5.0 mDa / DBE: min = -1.5, max = 100.0
Element prediction: Off
Number of isotope peaks used for i-FIT = 3

Monoisotopic Mass, Even Electron Ions
135 formula(e) evaluated with 1 results within limits (up to 50 closest results for each mass)
Elements Used:
C: 0-100 H: 0-250 N: 8-8 O: 0-20 F: 1-1 32S: 2-2
PO-15OCT21-175-SPOT-W-MEOH 105 (1.793) AM2 (Ar,25000.0,0.00,0.00); ABS
TOF MS ES+

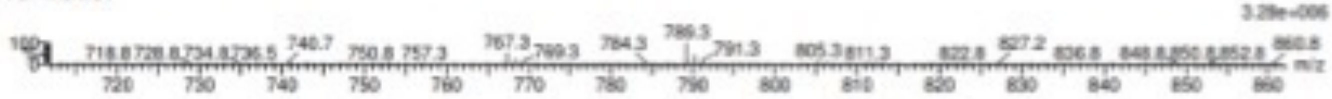

Minimum:				-1.5				
Maximum:		5.0	3.0	100.0				
Mass	Calc. Mass	mDa	PPM	DBE	i-FIT	Norm	Conf(%)	Formula
767.3028	767.3021	0.7	0.9	13.5	258.0	n/a	n/a	C33 H48 N8 O8 F 32S2

HPLC

Data File C:\Chem32\1\Data\PAOLA\Paola 2023-03-07 10-15-47\PO175-30000003.D
Sample Name: PO175 1M

```
=====================================================================
Acq. Operator   : SYSTEM                         Seq. Line :    1
Acq. Instrument : hplc                            Location :   11
Injection Date  : 3/7/2023 10:19:44 AM                 Inj :    1
                                                Inj Volume : 100.000 µl
Different Inj Volume from Sample Entry!  Actual Inj Volume : 5.000 µl
Method          : C:\Chem32\1\Data\PAOLA\Paola 2023-03-07 10-15-47\HBL1_A05-95_B95-05_20MIN.M
                  (Sequence Method)
Last changed    : 3/7/2023 10:15:47 AM by SYSTEM
Additional Info : Peak(s) manually integrated
```

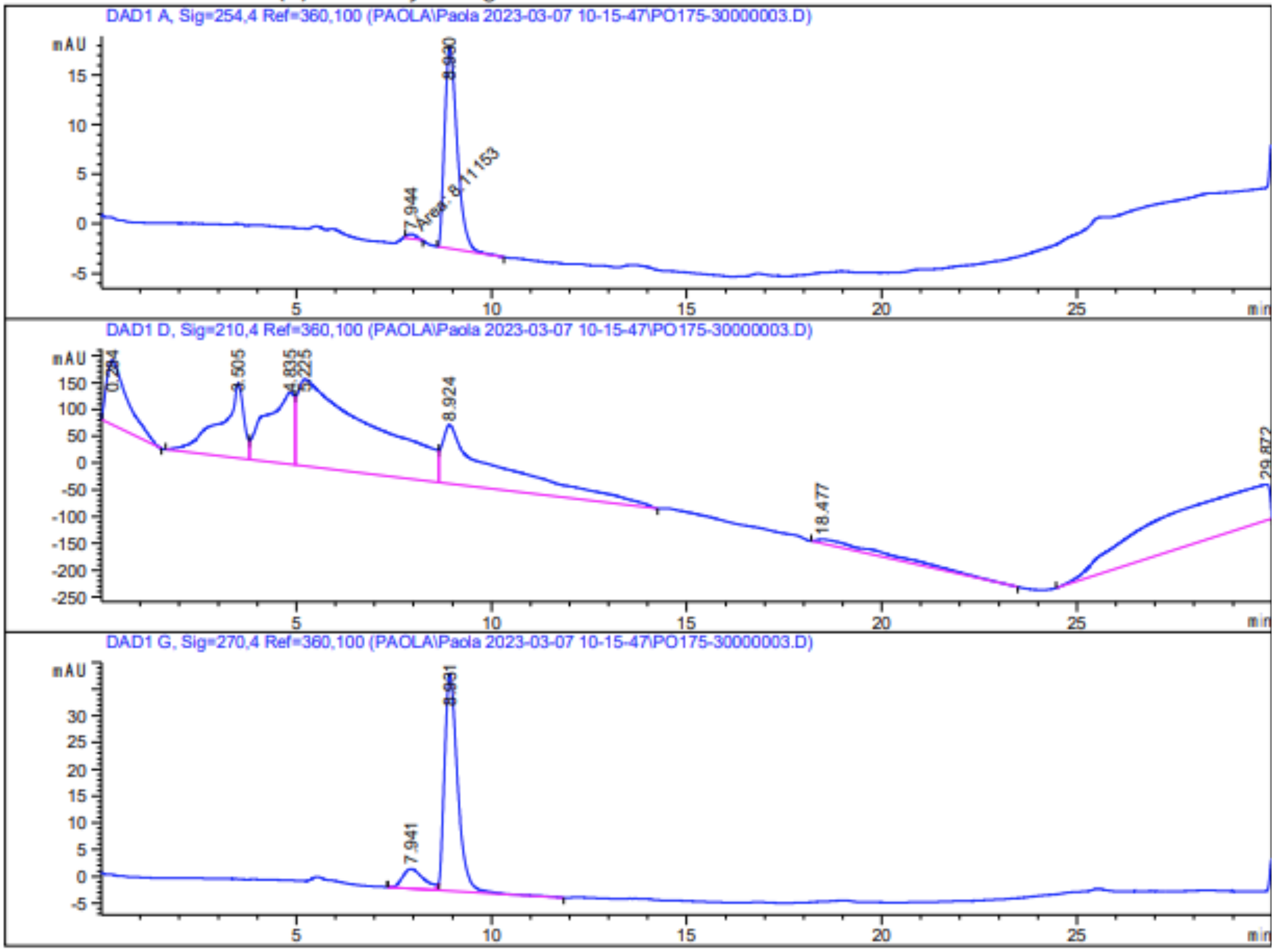


```
=====================================================================
                    Fraction Information
=====================================================================
Fraction collection using a timetable
=====================================================================
No Fractions found.
=====================================================================
```

===

Area Percent Report

===

Sorted By : Signal
Multiplier : 1.0000
Dilution : 1.0000
Sample Amount: : 1.00000 [ng/ul] (not used in calc.)
Use Multiplier & Dilution Factor with ISTDs

Signal 1: DAD1 A, Sig=254,4 Ref=360,100

Peak #	RetTime [min]	Type	Width [min]	Area [mAU*s]	Height [mAU]	Area %
1	7.944	MM	0.2853	8.11153	4.73866e-1	1.7432
2	8.930	BB	0.3507	457.20026	20.30611	98.2568
Totals :				465.31179	20.77997	

Signal 2: DAD1 D, Sig=210,4 Ref=360,100

Peak #	RetTime [min]	Type	Width [min]	Area [mAU*s]	Height [mAU]	Area %
1	0.284	BB	0.5443	4562.95166	120.36728	6.5770
2	3.505	BV	0.5593	5982.68262	139.24608	8.6234
3	4.835	VV	0.6122	6543.86475	134.84372	9.4323
4	5.225	VV	1.6796	2.22043e4	162.35585	32.0050
5	8.924	VB	1.2659	1.10729e4	109.48734	15.9603
6	18.477	BB	2.6296	1583.02612	7.24369	2.2818
7	29.872	BBA	4.2473	1.74278e4	68.38735	25.1202
Totals :				6.93775e4	741.93130	

Signal 3: DAD1 G, Sig=270,4 Ref=360,100

Peak #	RetTime [min]	Type	Width [min]	Area [mAU*s]	Height [mAU]	Area %
1	7.941	BV E	0.5270	136.89554	3.65441	12.6152
2	8.931	VB R	0.3585	948.26599	40.59351	87.3848
Totals :				1085.16153	44.24791	

===

*** End of Report ***

For Manuscript Review

Full wwPDB X-ray Structure Validation Report (i)

Mar 8, 2023 – 12:26 pm GMT

PDB ID	:	8CRC
Title	:	Structure of human Plk1-PBD in complex with Allopole-A
Deposited on	:	2023-03-08
Resolution	:	1.65 Å(reported)

This wwPDB validation report is for manuscript review

This is a Full wwPDB X-ray Structure Validation Report.

This report is produced by the wwPDB biocuration pipeline after annotation of the structure.

We welcome your comments at *validation@mail.wwpdb.org*
A user guide is available at
https://www.wwpdb.org/validation/2017/XrayValidationReportHelp
with specific help available everywhere you see the (i) symbol.

The types of validation reports are described at
http://www.wwpdb.org/validation/2017/FAQs#types.

The following versions of software and data (see references (i)) were used in the production of this report:

MolProbity	:	4.02b-467
Mogul	:	1.8.4, CSD as541be (2020)
Xtriage (Phenix)	:	1.13
EDS	:	2.32.1
buster-report	:	1.1.7 (2018)
Percentile statistics	:	20191225.v01 (using entries in the PDB archive December 25th 2019)
Refmac	:	5.8.0158
CCP4	:	7.0.044 (Gargrove)
Ideal geometry (proteins)	:	Engh & Huber (2001)
Ideal geometry (DNA, RNA)	:	Parkinson et al. (1996)

1 Overall quality at a glance (i)

The following experimental techniques were used to determine the structure:
X-RAY DIFFRACTION

The reported resolution of this entry is 1.65 Å.

Percentile scores (ranging between 0-100) for global validation metrics of the entry are shown in the following graphic. The table shows the number of entries on which the scores are based.

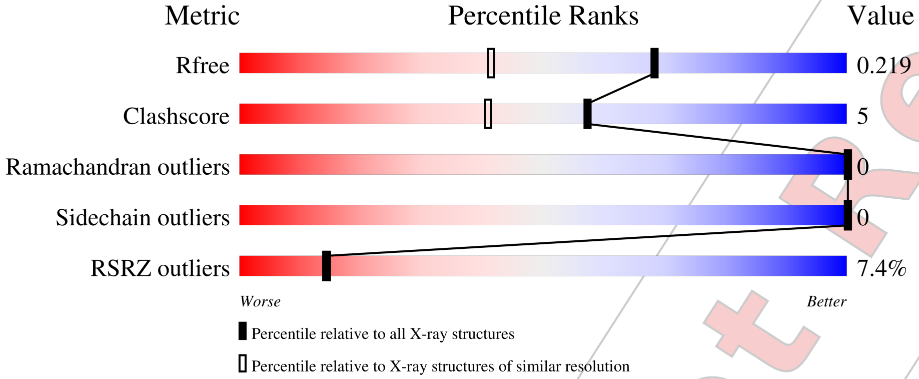

Metric	Whole archive (#Entries)	Similar resolution (#Entries, resolution range(Å))
R_{free}	130704	1827 (1.66-1.66)
Clashscore	141614	1931 (1.66-1.66)
Ramachandran outliers	138981	1891 (1.66-1.66)
Sidechain outliers	138945	1891 (1.66-1.66)
RSRZ outliers	127900	1791 (1.66-1.66)

The table below summarises the geometric issues observed across the polymeric chains and their fit to the electron density. The red, orange, yellow and green segments of the lower bar indicate the fraction of residues that contain outliers for >=3, 2, 1 and 0 types of geometric quality criteria respectively. A grey segment represents the fraction of residues that are not modelled. The numeric value for each fraction is indicated below the corresponding segment, with a dot representing fractions <=5% The upper red bar (where present) indicates the fraction of residues that have poor fit to the electron density. The numeric value is given above the bar.

Mol	Chain	Length	Quality of chain
1	A	228	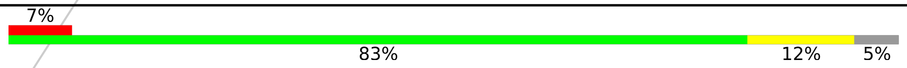

Validation Pipeline (wwPDB-VP) : 2.32.1

For Manuscript Review

2 Entry composition (i)

There are 4 unique types of molecules in this entry. The entry contains 1951 atoms, of which 0 are hydrogens and 0 are deuteriums.

In the tables below, the ZeroOcc column contains the number of atoms modelled with zero occupancy, the AltConf column contains the number of residues with at least one atom in alternate conformation and the Trace column contains the number of residues modelled with at most 2 atoms.

- Molecule 1 is a protein called Serine/threonine-protein kinase PLK1.

Mol	Chain	Residues	Atoms					ZeroOcc	AltConf	Trace
			Total	C	N	O	S			
1	A	217	1746	1119	294	321	12	0	8	0

There are 4 discrepancies between the modelled and reference sequences:

Chain	Residue	Modelled	Actual	Comment	Reference
A	367	GLY	-	expression tag	UNP P53350
A	368	ALA	-	expression tag	UNP P53350
A	369	HIS	-	expression tag	UNP P53350
A	370	MET	-	expression tag	UNP P53350

- Molecule 2 is GLYCEROL (three-letter code: GOL) (formula: $C_3H_8O_3$).

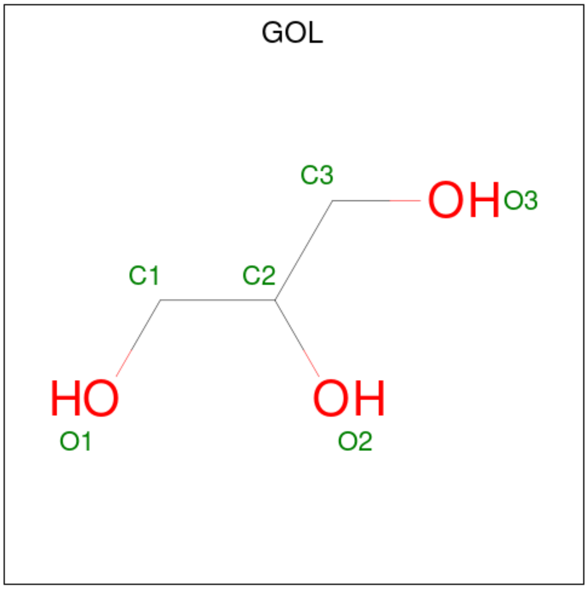

Mol	Chain	Residues	Atoms			ZeroOcc	AltConf
			Total	C	O		
2	A	1	6	3	3	0	0

For Manuscript Review

- Molecule 3 is 4-chloranyl-8-(cyclopropylmethyl)-12-sulfanylidene-5-thia-1,8,10,11-tetr azatricyclo[7.3.0.0^{2,6}]dodeca-2(6),3,9-trien-7-one (three-letter code: VIH) (formula: $C_{11}H_9ClN_4OS_2$) (labeled as "Ligand of Interest" by depositor).

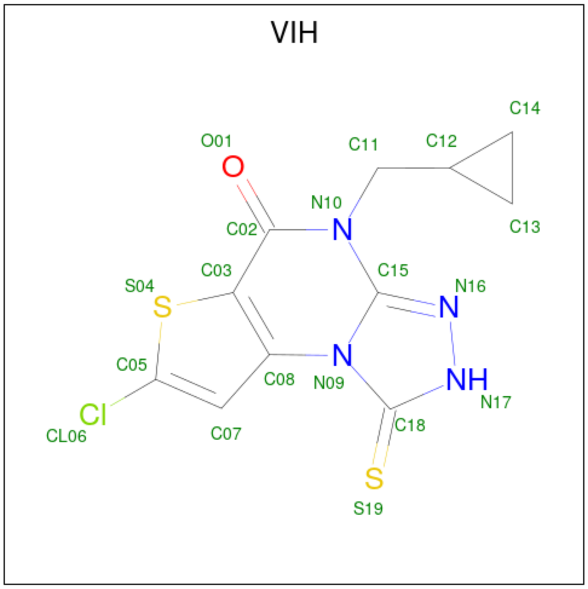

Mol	Chain	Residues	Atoms						ZeroOcc	AltConf
			Total	C	Cl	N	O	S		
3	A	1	19	11	1	4	1	2	0	0

- Molecule 4 is water.

Mol	Chain	Residues	Atoms		ZeroOcc	AltConf
			Total	O		
4	A	180	180	180	0	0

For Manuscript Review

3 Residue-property plots (i)

These plots are drawn for all protein, RNA, DNA and oligosaccharide chains in the entry. The first graphic for a chain summarises the proportions of the various outlier classes displayed in the second graphic. The second graphic shows the sequence view annotated by issues in geometry and electron density. Residues are color-coded according to the number of geometric quality criteria for which they contain at least one outlier: green = 0, yellow = 1, orange = 2 and red = 3 or more. A red dot above a residue indicates a poor fit to the electron density (RSRZ > 2). Stretches of 2 or more consecutive residues without any outlier are shown as a green connector. Residues present in the sample, but not in the model, are shown in grey.

- Molecule 1: Serine/threonine-protein kinase PLK1

Chain A:

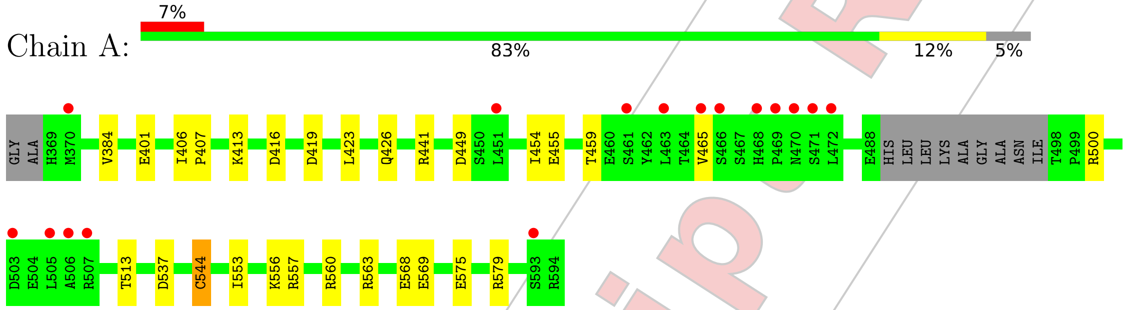

For Manuscript Review

4 Data and refinement statistics ⓘ

Property	Value	Source
Space group	P 1 21 1	Depositor
Cell constants<br>a, b, c, α, β, γ	39.36Å 51.91Å 51.45Å<br>90.00° 106.64° 90.00°	Depositor
Resolution (Å)	37.71 – 1.65<br>37.71 – 1.65	Depositor<br>EDS
% Data completeness<br>(in resolution range)	97.4 (37.71-1.65)<br>97.4 (37.71-1.65)	Depositor<br>EDS
R_{merge}	0.07	Depositor
R_{sym}	(Not available)	Depositor
$< I/\sigma(I) >$ [1]	2.19 (at 1.65Å)	Xtriage
Refinement program	PHENIX 1.18.2_3874	Depositor
R, R_{free}	0.177 , 0.219<br>0.177 , 0.219	Depositor<br>DCC
R_{free} test set	1170 reflections (5.00%)	wwPDB-VP
Wilson B-factor (Å^2)	22.9	Xtriage
Anisotropy	0.457	Xtriage
Bulk solvent k_{sol}(e/Å^3), B_{sol}(Å^2)	0.36 , 65.0	EDS
L-test for twinning[2]	$< \|L\| > = 0.51$, $< L^2 > = 0.34$	Xtriage
Estimated twinning fraction	No twinning to report.	Xtriage
F_o,F_c correlation	0.97	EDS
Total number of atoms	1951	wwPDB-VP
Average B, all atoms (Å^2)	37.0	wwPDB-VP

Xtriage's analysis on translational NCS is as follows: *The largest off-origin peak in the Patterson function is 9.59% of the height of the origin peak. No significant pseudotranslation is detected.*

[1]Intensities estimated from amplitudes.
[2]Theoretical values of $< |L| >$, $< L^2 >$ for acentric reflections are 0.5, 0.333 respectively for untwinned datasets, and 0.375, 0.2 for perfectly twinned datasets.

For Manuscript Review

5 Model quality (i)

5.1 Standard geometry (i)

Bond lengths and bond angles in the following residue types are not validated in this section: VIH, GOL

The Z score for a bond length (or angle) is the number of standard deviations the observed value is removed from the expected value. A bond length (or angle) with $|Z| > 5$ is considered an outlier worth inspection. RMSZ is the root-mean-square of all Z scores of the bond lengths (or angles).

Mol	Chain	Bond lengths		Bond angles	
		RMSZ	$\#|Z| > 5$	RMSZ	$\#|Z| > 5$
1	A	0.61	2/1806 (0.1%)	0.78	1/2447 (0.0%)

All (2) bond length outliers are listed below:

Mol	Chain	Res	Type	Atoms	Z	Observed(Å)	Ideal(Å)
1	A	544[A]	CYS	CB-SG	5.97	1.92	1.82
1	A	544[B]	CYS	CB-SG	5.97	1.92	1.82

All (1) bond angle outliers are listed below:

Mol	Chain	Res	Type	Atoms	Z	Observed(^{o})	Ideal(^{o})
1	A	500	ARG	CG-CD-NE	7.53	127.62	111.80

There are no chirality outliers.

There are no planarity outliers.

5.2 Too-close contacts (i)

In the following table, the Non-H and H(model) columns list the number of non-hydrogen atoms and hydrogen atoms in the chain respectively. The H(added) column lists the number of hydrogen atoms added and optimized by MolProbity. The Clashes column lists the number of clashes within the asymmetric unit, whereas Symm-Clashes lists symmetry-related clashes.

Mol	Chain	Non-H	H(model)	H(added)	Clashes	Symm-Clashes
1	A	1746	0	1706	17	0
2	A	6	0	8	0	0
3	A	19	0	0	1	0
4	A	180	0	0	4	0
All	All	1951	0	1714	17	0

For Manuscript Review

The all-atom clashscore is defined as the number of clashes found per 1000 atoms (including hydrogen atoms). The all-atom clashscore for this structure is 5.

All (17) close contacts within the same asymmetric unit are listed below, sorted by their clash magnitude.

Atom-1	Atom-2	Interatomic distance (Å)	Clash overlap (Å)
1:A:384:VAL:HA	1:A:568:GLU:HG2	1.78	0.65
1:A:569:GLU:OE2	4:A:701:HOH:O	2.18	0.56
1:A:416:ASP:HA	1:A:423:LEU:HD12	1.89	0.55
1:A:441:ARG:HB2	1:A:454:ILE:HB	1.91	0.52
1:A:544[B]:CYS:SG	4:A:714:HOH:O	2.58	0.51
1:A:419:ASP:OD2	1:A:419:ASP:N	2.45	0.50
1:A:449:ASP:OD1	1:A:465:VAL:HG12	2.16	0.46
1:A:455:GLU:HG3	1:A:459:THR:OG1	2.16	0.46
1:A:513:THR:OG1	4:A:702:HOH:O	2.21	0.44
1:A:563[A]:ARG:NH1	4:A:712:HOH:O	2.51	0.44
1:A:575:GLU:O	1:A:579:ARG:HG2	2.18	0.43
1:A:553:ILE:HG21	3:A:602:VIH:CL06	2.56	0.42
1:A:537:ASP:OD2	1:A:579:ARG:NH2	2.49	0.42
1:A:413:LYS:CB	1:A:426:GLN:HG2	2.50	0.41
1:A:401:GLU:OE2	1:A:560[A]:ARG:HD2	2.21	0.41
1:A:556:LYS:O	1:A:557:ARG:HB2	2.20	0.41
1:A:406[A]:ILE:HD12	1:A:407:PRO:HD2	2.03	0.40

There are no symmetry-related clashes.

5.3 Torsion angles (i)

5.3.1 Protein backbone (i)

In the following table, the Percentiles column shows the percent Ramachandran outliers of the chain as a percentile score with respect to all X-ray entries followed by that with respect to entries of similar resolution.

The Analysed column shows the number of residues for which the backbone conformation was analysed, and the total number of residues.

Mol	Chain	Analysed	Favoured	Allowed	Outliers	Percentiles	
1	A	221/228 (97%)	211 (96%)	10 (4%)	0	100	100

There are no Ramachandran outliers to report.

For Manuscript Review

5.3.2 Protein sidechains (i)

In the following table, the Percentiles column shows the percent sidechain outliers of the chain as a percentile score with respect to all X-ray entries followed by that with respect to entries of similar resolution.

The Analysed column shows the number of residues for which the sidechain conformation was analysed, and the total number of residues.

Mol	Chain	Analysed	Rotameric	Outliers	Percentiles	
1	A	187/205 (91%)	187 (100%)	0	100	100

There are no protein residues with a non-rotameric sidechain to report.

Sometimes sidechains can be flipped to improve hydrogen bonding and reduce clashes. All (1) such sidechains are listed below:

Mol	Chain	Res	Type
1	A	452	GLN

5.3.3 RNA (i)

There are no RNA molecules in this entry.

5.4 Non-standard residues in protein, DNA, RNA chains (i)

There are no non-standard protein/DNA/RNA residues in this entry.

5.5 Carbohydrates (i)

There are no monosaccharides in this entry.

5.6 Ligand geometry (i)

2 ligands are modelled in this entry.

In the following table, the Counts columns list the number of bonds (or angles) for which Mogul statistics could be retrieved, the number of bonds (or angles) that are observed in the model and the number of bonds (or angles) that are defined in the Chemical Component Dictionary. The Link column lists molecule types, if any, to which the group is linked. The Z score for a bond length (or angle) is the number of standard deviations the observed value is removed from the expected value. A bond length (or angle) with $|Z| > 2$ is considered an outlier worth inspection. RMSZ is the root-mean-square of all Z scores of the bond lengths (or angles).

For Manuscript Review

Mol	Type	Chain	Res	Link	Bond lengths			Bond angles		
					Counts	RMSZ	#|Z| > 2	Counts	RMSZ	#|Z| > 2
2	GOL	A	601	-	5,5,5	1.02	0	5,5,5	0.89	0
3	VIH	A	602	-	19,22,22	1.81	3 (15%)	15,34,34	1.74	2 (13%)

In the following table, the Chirals column lists the number of chiral outliers, the number of chiral centers analysed, the number of these observed in the model and the number defined in the Chemical Component Dictionary. Similar counts are reported in the Torsion and Rings columns. '-' means no outliers of that kind were identified.

Mol	Type	Chain	Res	Link	Chirals	Torsions	Rings
2	GOL	A	601	-	-	2/4/4/4	-
3	VIH	A	602	-	-	0/4/6/6	0/4/4/4

All (3) bond length outliers are listed below:

Mol	Chain	Res	Type	Atoms	Z	Observed(Å)	Ideal(Å)
3	A	602	VIH	C18-N17	5.01	1.40	1.34
3	A	602	VIH	C07-C08	4.28	1.43	1.39
3	A	602	VIH	C08-N09	-3.16	1.38	1.42

All (2) bond angle outliers are listed below:

Mol	Chain	Res	Type	Atoms	Z	Observed(^{o})	Ideal(^{o})
3	A	602	VIH	C18-N17-N16	-4.33	111.21	113.95
3	A	602	VIH	O01-C02-N10	2.57	123.77	120.13

There are no chirality outliers.

All (2) torsion outliers are listed below:

Mol	Chain	Res	Type	Atoms
2	A	601	GOL	O1-C1-C2-C3
2	A	601	GOL	O1-C1-C2-O2

There are no ring outliers.

1 monomer is involved in 1 short contact:

Mol	Chain	Res	Type	Clashes	Symm-Clashes
3	A	602	VIH	1	0

The following is a two-dimensional graphical depiction of Mogul quality analysis of bond lengths, bond angles, torsion angles, and ring geometry for all instances of the Ligand of Interest. In

For Manuscript Review

addition, ligands with molecular weight > 250 and outliers as shown on the validation Tables will also be included. For torsion angles, if less then 5% of the Mogul distribution of torsion angles is within 10 degrees of the torsion angle in question, then that torsion angle is considered an outlier. Any bond that is central to one or more torsion angles identified as an outlier by Mogul will be highlighted in the graph. For rings, the root-mean-square deviation (RMSD) between the ring in question and similar rings identified by Mogul is calculated over all ring torsion angles. If the average RMSD is greater than 60 degrees and the minimal RMSD between the ring in question and any Mogul-identified rings is also greater than 60 degrees, then that ring is considered an outlier. The outliers are highlighted in purple. The color gray indicates Mogul did not find sufficient equivalents in the CSD to analyse the geometry.

Ligand VIH A 602

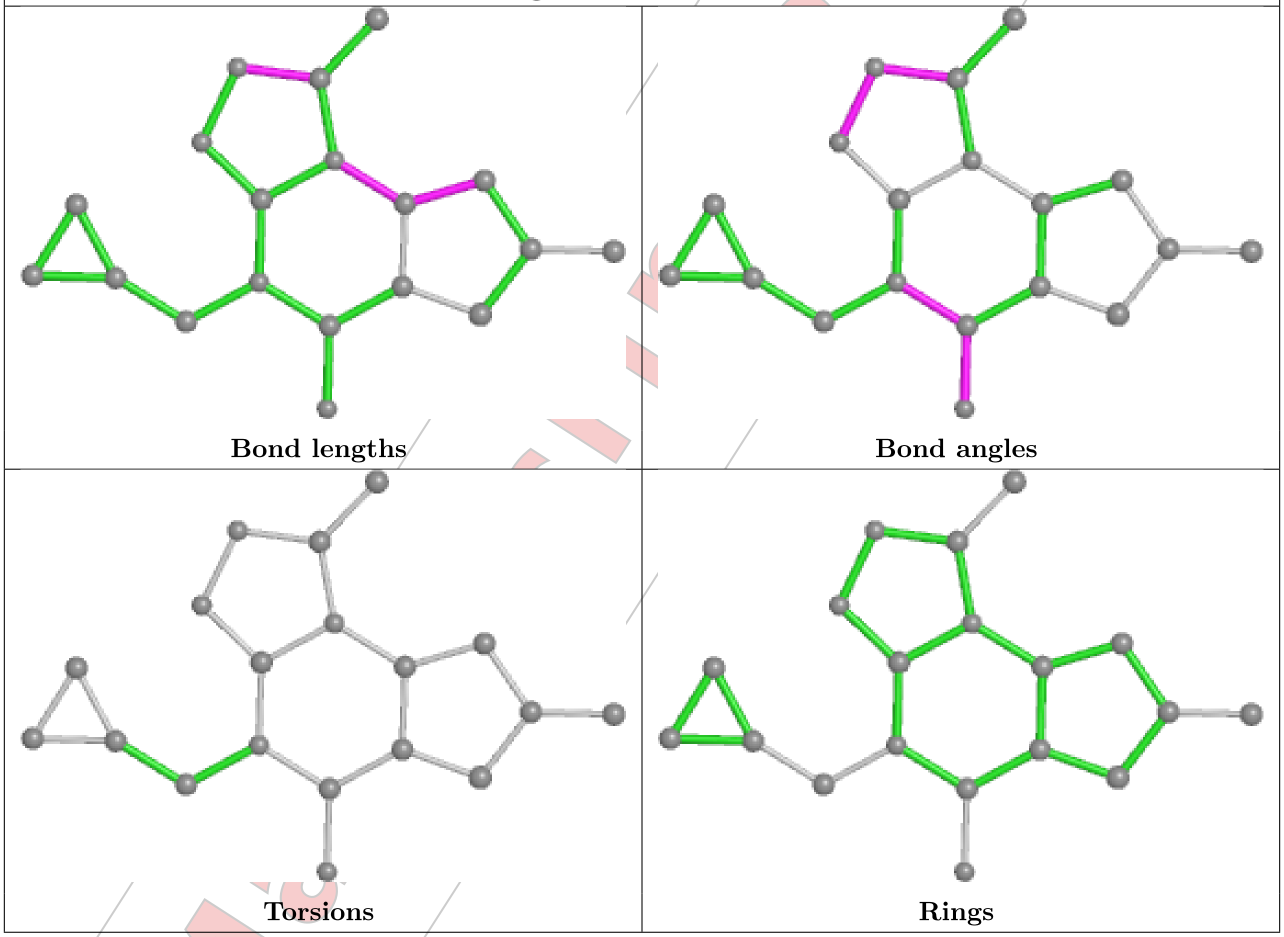

5.7 Other polymers (i)

There are no such residues in this entry.

5.8 Polymer linkage issues (i)

There are no chain breaks in this entry.

For Manuscript Review

6 Fit of model and data (i)

6.1 Protein, DNA and RNA chains (i)

In the following table, the column labelled '#RSRZ> 2' contains the number (and percentage) of RSRZ outliers, followed by percent RSRZ outliers for the chain as percentile scores relative to all X-ray entries and entries of similar resolution. The OWAB column contains the minimum, median, 95th percentile and maximum values of the occupancy-weighted average B-factor per residue. The column labelled 'Q< 0.9' lists the number of (and percentage) of residues with an average occupancy less than 0.9.

Mol	Chain	Analysed	<RSRZ>	#RSRZ>2			OWAB(Å^2)	Q<0.9
1	A	217/228 (95%)	0.01	16 (7%)	14	14	18, 31, 72, 122	0

All (16) RSRZ outliers are listed below:

Mol	Chain	Res	Type	RSRZ
1	A	506	ALA	8.5
1	A	505	LEU	4.1
1	A	472	LEU	4.0
1	A	503	ASP	3.8
1	A	468	HIS	3.6
1	A	469	PRO	3.3
1	A	370	MET	3.2
1	A	470	ASN	3.0
1	A	465	VAL	2.9
1	A	507	ARG	2.9
1	A	471	SER	2.8
1	A	593	SER	2.6
1	A	466	SER	2.1
1	A	461	SER	2.1
1	A	451	LEU	2.1
1	A	463	LEU	2.0

6.2 Non-standard residues in protein, DNA, RNA chains (i)

There are no non-standard protein/DNA/RNA residues in this entry.

6.3 Carbohydrates (i)

There are no monosaccharides in this entry.

For Manuscript Review

6.4 Ligands (i)

In the following table, the Atoms column lists the number of modelled atoms in the group and the number defined in the chemical component dictionary. The B-factors column lists the minimum, median, 95th percentile and maximum values of B factors of atoms in the group. The column labelled 'Q< 0.9' lists the number of atoms with occupancy less than 0.9.

Mol	Type	Chain	Res	Atoms	RSCC	RSR	B-factors($Å^2$)	Q<0.9
2	GOL	A	601	6/6	0.62	0.29	54,67,73,76	0
3	VIH	A	602	19/19	0.96	0.08	24,26,32,85	0

The following is a graphical depiction of the model fit to experimental electron density of all instances of the Ligand of Interest. In addition, ligands with molecular weight > 250 and outliers as shown on the geometry validation Tables will also be included. Each fit is shown from different orientation to approximate a three-dimensional view.

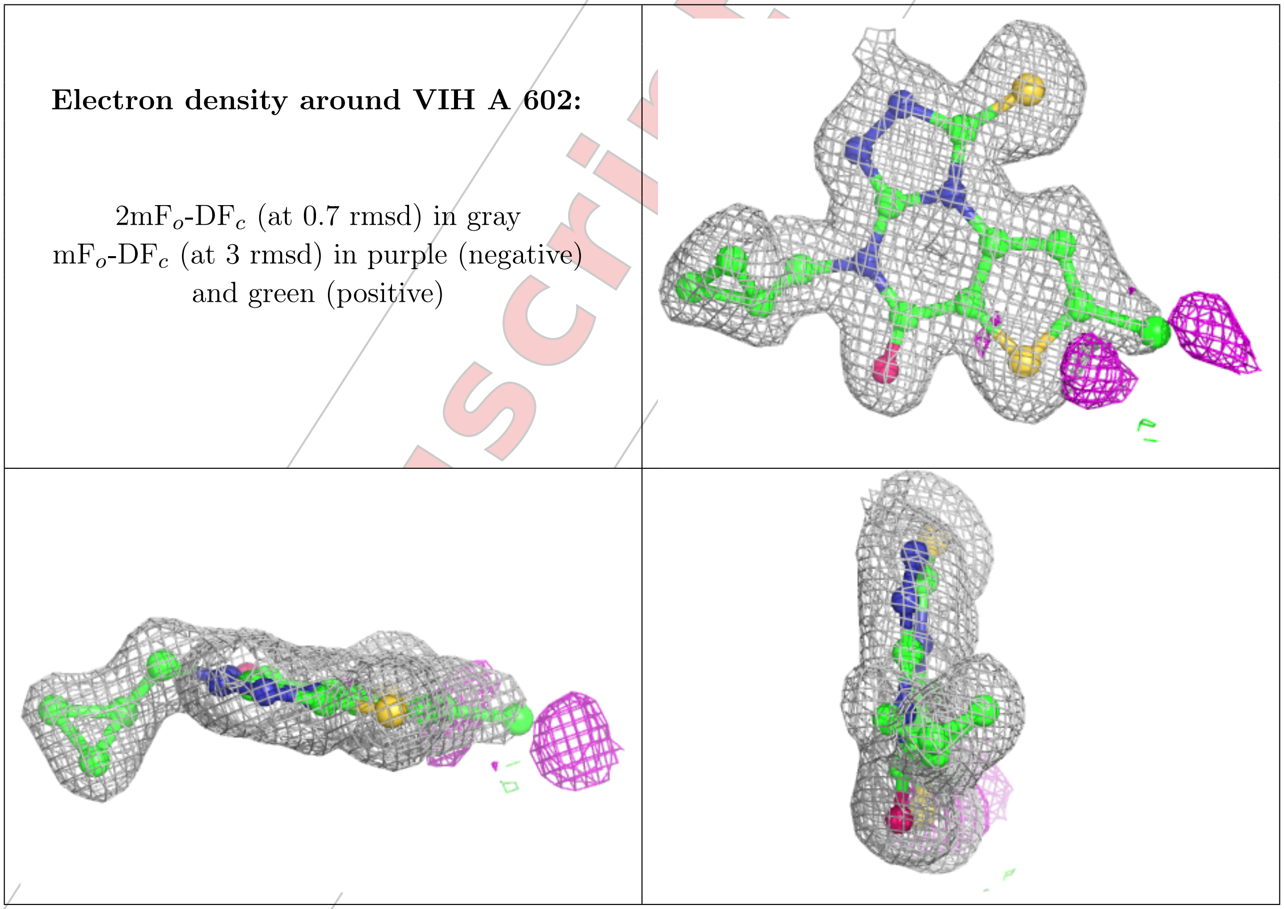

Electron density around VIH A 602:

$2mF_o$-DF_c (at 0.7 rmsd) in gray
mF_o-DF_c (at 3 rmsd) in purple (negative) and green (positive)

6.5 Other polymers (i)

There are no such residues in this entry.

For Manuscript Review
